# Dendritic nonlinearities mitigate communication costs

**DOI:** 10.1016/j.patter.2026.101520

**Published:** 2026-03-30

**Authors:** Xundong Wu, Pengfei Zhao, Zilin Yu, Lei Ma, Yifan Gao, Ka-Wa Yip, Huajin Tang, Gang Pan, Panayiota Poirazi, Tiejun Huang

**Affiliations:** 1 Zhejiang Lab, Hangzhou, Zhejiang, China; 2 Beijing Academy of Artificial Intelligence, Beijing, China; 3 Bytedance, Beijing, China; 4 National Key Laboratory for Multimedia Information Processing, School of Computer Science, Peking University, Beijing, China; 5 National Biomedical Imaging Center, Peking University, Beijing, China; 6 Zhejiang University, Hangzhou, China; 7 IMBB-FORTH, Heraklion, Crete, Greece

**Keywords:** dendritic nonlinearities, communication costs, localized feature aggregation, active dendrites, neural network, algorithm-hardware co-design, neuromorphic computing, brain-inspired computing, network scaling

## Abstract

Why have modern artificial neural networks not adopted the nonlinear dendritic structures found in biological brain cells, and what is the core advantage of such active dendritic units? While early studies suggested that dendritic nonlinearities can enhance learning capabilities by boosting capacity, we provide empirical evidence reassessing this. Using extensive machine learning experiments, we show that dendritic nonlinearities in neural networks offer comparable learning capacity to standard point-neuron models when controlled for parametric complexity. Instead, we believe that their key advantage lies in enabling network scaling while substantially reducing communication costs via localized feature aggregation. Our experiments and analysis suggest that incorporating nonlinear dendritic architectures can significantly lower memory access or data transfer overhead during neural network inference—the primary sources of energy consumption in modern AI systems—and potentially during training as well. We argue that these insights motivate further theoretical and architectural exploration of dendritic-like structures in artificial neural networks.

## Highlights

• Dendritic nonlinearities reduce communication costs in neural networks
• Active dendrites match point-neuron capacity when controlling for complexity
• Localized feature aggregation significantly lowers memory access overhead
• Dendritic structures enable efficient network scaling

## The bigger picture

Modern AI relies heavily on simplified “point neurons.” While there is active research on complex, bio-inspired dendritic units, their adoption remains limited. We argue that active dendritic units offer learning capacity comparable to point neurons, their real evolutionary and engineering advantage lies in minimizing communication costs. By mimicking the brain’s strategy of using active dendrites to nonlinearly aggregate features locally, we can design neural architectures that scale efficiently without a prohibitive increase in data traffic. Research in this direction could have important implications for the development of future AI hardware and software. By shifting architectural priorities from dense signal transmission to optimizing local feature aggregation, neural network hardware can be substantially more efficient. Achieving sustainable, large-scale AI requires us to re-imagine the neuron not just as a calculator but as a communication optimizer. By organizing these units in a way that respects the physical constraints of data movement—similar to how brains are optimized for wiring efficiency—we can overcome the connectivity bottlenecks that currently limit the scaling of artificial intelligence.

## Teaser

Artificial neural networks typically ignore the complex, active branches found in biological neurons. This study challenges the assumption that these “dendritic nonlinearities” primarily boost computational power. Instead, the authors show they function like efficient data filters. By processing signals locally before transmission, these nonlinear structures drastically reduce the “traffic” required between neurons. This reveals a biological blueprint for scaling AI by solving communication bottlenecks rather than just increasing calculation capacity.

## Introduction

**Figure 1:**
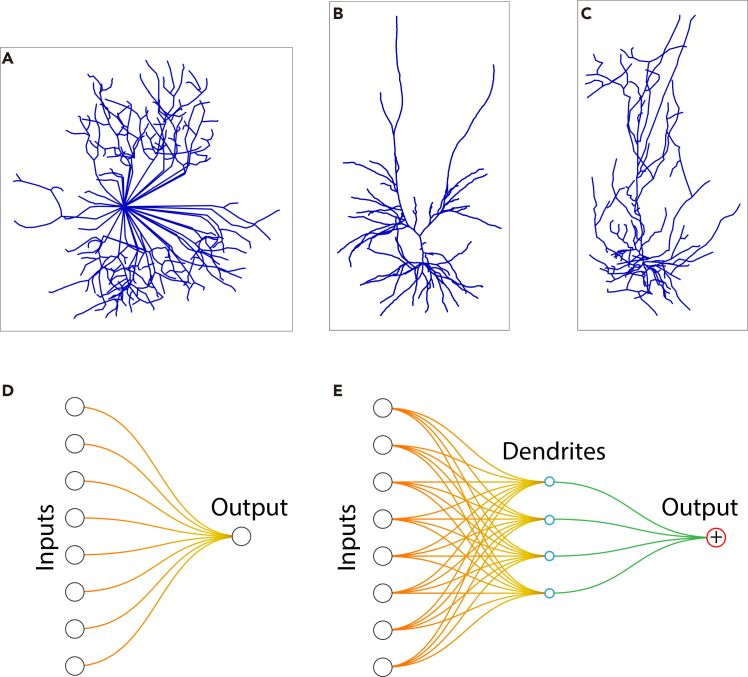
Representative biological neurons illustrating dendritic structures contrasted with mathematical point and dendritic neuron models (A–C) Illustration of three representative neurons showcasing distinct dendritic structures from left to right: a chicken bipolar neuron,^3^ a human hippocampal pyramidal neuron,^4^ and a ferret neocortical pyramidal neuron.^5^ All neuronal morphologies are from the Neuromorpho.org database.^6^ (D) Portrays a point neuron, as characterized by Equation 1. (E) Illustrates a dendritic neuron with 4 dendritic branches as detailed by Equations 2 and 3.

Over the past decade, artificial neural networks (ANNs) have significantly advanced across diverse domains, demonstrating impressive performance on complex tasks.^1^^,^^2^ Despite their inspiration from biological neuronal networks, modern ANNs use highly simplified “point neurons,” described mathematically as

(Equation 1) $$h=\sigma \left(\sum_{i=1}^{n}w_{i}x_{i}+b\right)\text{,}$$

where inputs (*x*_*i*_), weights (*w*_*i*_), bias (*b*), and nonlinear activation (*σ*) produce neuron output (*h*). These neurons differ drastically from biological neurons, which have elaborate dendritic structures (Figure 1).

Dendritic structures in biological neurons offer enhanced surface-area-to-volume ratios, essential for forming numerous synaptic connections within limited brain space.^7^^,^^8^^,^^9^ Unlike biological brains, ANNs executed on general-purpose hardware (CPUs and GPUs) do not have physical constraints, as in biological brains, raising questions about the necessity of dendrites in artificial systems. Here, we argue that dendrites are indeed valuable beyond biological contexts.

Dendrites are known to facilitate local nonlinear operations due to their anatomical compartmentalization and specialized voltage-gated ion channels.^7^^,^^10^^,^^11^^,^^12^ These localized nonlinearities may enable key computational functions such as coincidence detection, signal amplification, learning, context association, enhanced feature extraction, and temporal discrimination.^13^^,^^14^^,^^15^^,^^16^^,^^17^ Most importantly, it has long been believed that dendritic nonlinearities can endow neurons with greater model capacity than point-neuron-based models,^13^^,^^18^ among many others.

However, our findings challenge this assumption. Approaching the question from a machine learning perspective, we demonstrate that adopting neurons with active dendrites has little effect on model learning capacity. Instead, we show that adopting active dendrites can significantly reduce communication costs in neural network models. Specifically, dendritic architectures enable localized processing that reduces the number of transmitted features without degrading performance. This is particularly important because communication overhead—primarily from data movement—dominates energy consumption in ANNs,^19^ echoing biological evidence that highlights the high cost of axonal transmission relative to local computation.^20^

Our findings indicate that the dendritic-neuron-based model’s expanded learning capacity^13^^,^^18^ likely arises primarily from the sparse structure employed in their models and redundancy avoidance attributed to the smaller unit size of dendrites. Thus, we posit that the primary advantage of active dendrites lies in mitigating communication costs through effective local feature aggregation.

A recent publication^21^ also employs nonlinear dendritic components and demonstrates marked improvements in parameter efficiency. While their conclusions may seem to differ from ours at first sight, the two studies address complementary aspects of dendritic computation. The analysis in Chavlis and Poirazi^21^ emphasizes architectural and sampling-related benefits, without specifically separating the effects of weight sparsity. The present work aims to clarify the distinct sources of dendritic advantages reported in those earlier studies by systematically isolating the role of dendritic nonlinearities. Our findings suggest that these nonlinearities offer intrinsic benefits in reducing communication costs—an aspect particularly relevant for both modern neural network hardware and the evolutionary optimization of biological neural systems.

Our investigation also finds that adopting a dendritic structure can potentially significantly reduce memory access and occupancy during inference and training of ANN models. These results have important implications for the development of efficient ANN architectures and hardware for real-world applications.

In summary, this work makes the following contributions.

• We demonstrate that dendritic nonlinearities provide comparable learning capacity to point neurons when normalized for computational complexity, challenging the assumption that their primary benefit is enhanced expressivity.
• We identify reduced communication cost as the primary advantage of active dendrites, enabling efficient local feature aggregation that scales effectively with network size.
• We provide a theoretical framework and empirical evidence showing that dendritic architectures can significantly lower memory access overhead during ANN inference and training.

## Results

### Mathematical formulation of the dendritic neuron

In this study, we use a simplified dendritic neuron model,^10^^,^^22^ which is depicted in Figure 1E and mathematically described by Equations 2 and 3. In this architecture, incoming signals at each dendrite are integrated by computing the dendritic output *d*_*j*_, which is obtained from the weight vector ***w***_*j*_, input activation ***x***, and an optional bias *b*_*j*_. The dendritic output is transformed by an element-wise nonlinear function *σ* and subsequently summed to produce the somatic output *h*, conveyed to downstream recipients:

(Equation 2) $${\hat{d}}_{j}=w_{j}^{⊤}x+b_{j},d_{j}=\sigma \left({\hat{d}}_{j}\right)$$

(Equation 3) $$h=\sum_{j=1}^{K}d_{j}\;\text{.}$$

Each dendritic unit described here has the same information-processing capacity as a point neuron but differs in how its output is conveyed downstream. Unlike point neurons, whose outputs are independently transmitted to downstream neurons, dendrites share a common channel for output transmission, typically resulting in information loss. This property is detailed in Note S1.

### Communication versus computing in neural networks

In neural networks, the energy required for computation is substantial, but communication is the primary energy bottleneck in modern hardware.^19^ Communication can incur orders-of-magnitude higher costs than computation; for instance, as reviewed by Dally et al.,^19^ adding two 32-bit numbers may consume approximately 20 femtojoules (fJ), while fetching these numbers from memory can require about 1.3 nanojoules (nJ)—roughly 64,000 times more energy.

Biological brains also exhibit significant energy expenditure and structural investment related to communication, as indicated by extensive white matter volume^23^ and high metabolic demands.^20^^,^^24^^,^^25^ The evolutionary pressure on biological systems to minimize these costs suggests potential insights for artificial systems. Our study proposes that incorporating active dendrites into ANNs can significantly mitigate communication-related energy expenses.

### Evaluating the communication efficiency of the dendritic structure

#### Developing the dendritic neuron model

To investigate the role active dendrites can play in neural networks, we compare the performance of models that are constituted with point neurons or dendritic neurons, respectively. We substitute the point neurons in conventional neural network models with dendritic neurons. Each nonlinear summation unit—point neuron or active dendrite—receives at most one copy of a specific input from the previous network layer. Each dendrite within a neuron receives an equal number of dense connections; hence, a dendritic neuron with *K* branches receives *K* times more inputs than its point-neuron counterpart. Clearly, those two models are of very different computing and parametric complexity. We need to compare models on an equal footing.

**Figure 2:**
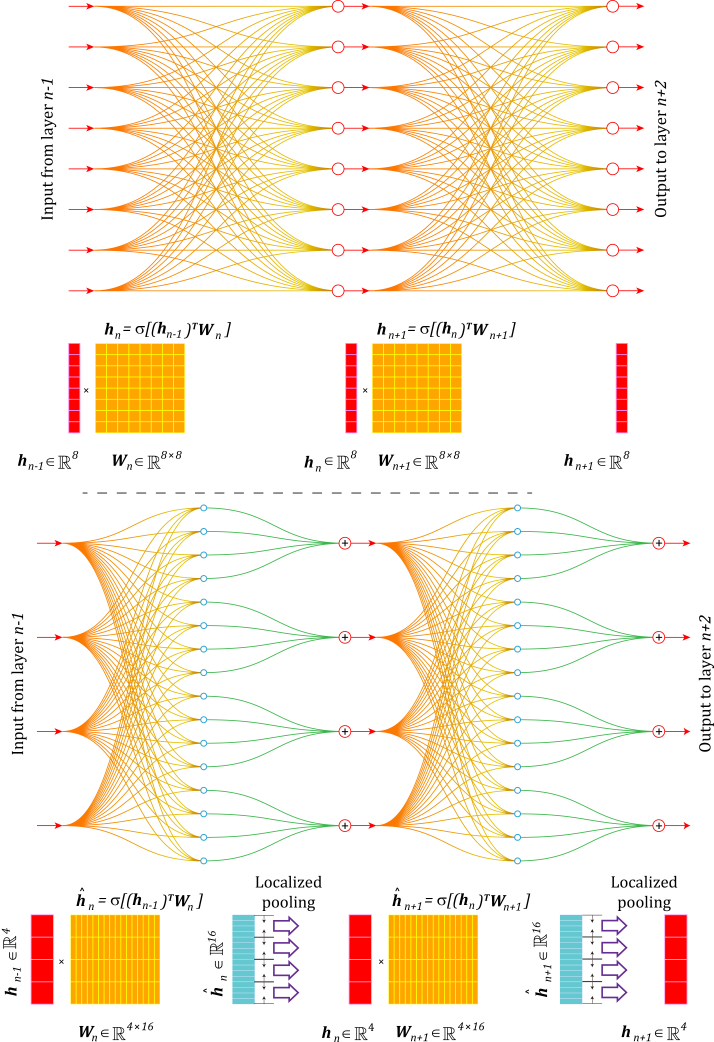
Structural comparison of neural network layers utilizing point neurons versus dendritic neurons Comparison of neural network layers using point neurons (top) and dendritic neurons (bottom). Only two layers from each model are depicted. The point-neuron model has *D* = 8 channels, whereas the dendritic neuron model features neurons with *K* = 4 dendritic branches each, leading to an effective $\hat{D}=\frac{D}{\sqrt{4}}=4$ channels. This ensures that both models have comparable parametric and computational complexities. Note: tensor dimensions are symbolized by a mesh of patches; however, patch sizes do not reflect actual scale. Bias terms have been excluded for simplicity.

Figure 2 visually compares how layers of point neurons (top) and dendritic neurons (bottom) are structured in a neural network. Consider, for instance, a point-neuron-based fully connected layer with *D*_*point*_ = 8 inputs and *D*_*point*_ = 8 output neurons (top); its parametric/computing complexity is 8^2^ = 64. Now, consider a dendritic neuron layer (bottom) with *D*_*dendritic*_ input/output neurons and *K* dendrites per neuron—its parametric/computing complexity is $KD_{dendritic}^{2}$. To make these layers comparable in complexity, we need to set $D_{dendritic}=D_{point}/\sqrt{K}$. So for our example with *D*_*point*_ = 8 and *K* = 4, we set *D*_*dendritc*_ = 4. Throughout this study, because parametric and computational complexities always scale identically, we will primarily refer to parametric complexity for simplicity.

To compare communication costs, let *D* be the total number of neurons in a layer or network and define Ψ = *D*_*dendritic*_/*D*_*point*_ as the ratio of *D* relative to a point-neuron baseline. In the example, the point-neuron layer has *D*_*point*_ = 8, while the dendritic layer has *D*_*dendritic*_ = 4, resulting in Ψ = 0.5.

#### Dense models on ImageNet

We begin by employing the ResNet-18 network^26^ as a baseline point-neuron-based model, a widely utilized computer vision model. For this set of experiments, we modify the ResNet-18 network architecture as described above to replace typical point neurons with dendritic neurons. Since the dimensionality of our network’s input and output remains fixed, the input and output layers are addressed differently, as detailed in the model architectures section.

**Figure 3:**
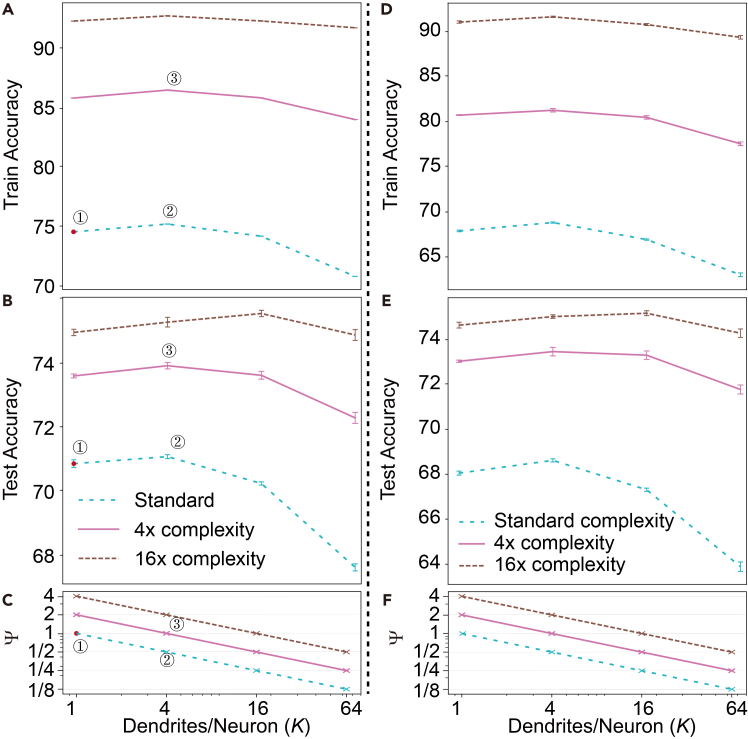
Performance analysis of dense and sparse ResNet-18 models on ImageNet using point versus dendritic neurons (A–C) Experiment on dense models (5 trials; SD shown). The red dot (①) marks the baseline ResNet-18 with standard point neurons (*K* = 1). The *x* axis indicates the number of dendrites per neuron (*K*). Three complexity levels are evaluated: standard complexity (light-blue dashed curves), models with the same complexity as baseline ResNet-18. 4× complexity (solid magenta curves), and 16× complexity (brown dashed curves). Within each curve, models share the same total parametric budget but differ in *K* (number of dendrites per neuron). For example, model ② is configured with a Ψ ratio of 0.5 to match the complexity of the baseline. Model ③, with Ψ = 1 and *K* = 4, has 4× the complexity of the baseline model ①. (A) Training set accuracy. (B) Test set accuracy. (C) Ψ ratio relative to the baseline ResNet-18. (D–F) Same layout and analysis as in (A)–(C) but for sparse models (3 trials; SD shown).

The outcomes of this experiment are shown in Figure 3. We compare models with three levels of complexity. The light-blue dashed curves represent experimental results obtained from various models with a complexity level equal to that of the standard ResNet-18 model. The leftmost data point (①) corresponds to the standard ResNet-18 model, which serves as a baseline. Subsequent data points to the right denote dendritic models with *K* values of 4, 16, and 64, respectively. Concomitantly, these models’ *D* values have been adjusted to 1/2, 1/4, and 1/8 of the original model’s values, respectively. Given that the models we study here are convolutional neural networks, we change *D* by scaling up/down the number of channels in network layers. By maintaining this configuration, four models on the same curve have Ψ values of 1, 0.5, 0.25, and 0.125 from left to right, as shown in Figure 3C, all while preserving equivalent parametric and computational complexities (see Note S2 for a detailed complexity comparison between models).

The solid magenta curves represent data from models where *D* is doubled compared to the experiments shown by the light-blue curve. Similarly, the brown dashed curves illustrate models where *D* values are quadrupled. For the brown dashed curve, the point-neuron-based model at the left end of the curve has 4 times the number of neurons (Ψ = 4 in Figure 3C) for each layer compared to the standard ResNet-18 model (①). At the right end of the brown curve, we can see the dendritic model, which is equipped with *K* = 64 dendrites per neuron, thus having just one-eighth of neurons (Ψ = 0.125).

Our analysis yields a particularly intriguing result concerning the communication cost of dendritic neuron models compared to point-neuron-based models. Specifically, we find that for models of equivalent computing complexity, a dendritic neuron model achieves comparable performance to a point-neuron-based model when Ψ is greater than or equal to 0.25.

This reduction in *D* offered by adding dendrites can significantly reduce the communication cost between neurons in ANNs.

To obtain a more complete picture, we also compare models with the same number of neurons. The results are illustrated in Figure S7.

#### Sparse models on ImageNet

Thus far, we developed dendritic-neuron-based models with significantly reduced communication costs, as measured by *D*. These dendritic-neuron-based models can also achieve similar (or slightly better) performance compared to corresponding point-neuron-based models of the same computing complexity, in terms of both model expressivity and generalization performance.

This may seem contradictory to earlier works,^13^^,^^18^ where dendritic-neuron-based models showed higher capacity/expressivity than point-neuron-based models of the same parametric complexity. One might argue that the models we evaluated thus far are non-sparse, which is not biological and differs from the models evaluated in earlier studies. As such, it is essential to also investigate the influence of sparsity on model behavior.

As illustrated on the right side of Figure 3, we study sparse models with 85% of parameters pruned. Although sparsity generally reduces performance, we observe the same trend as in the non-sparse case: models with different dendritic numbers *K* but equal computing complexity show little performance difference between dendritic and point-neuron architectures as long as Ψ is above 0.25. This reinforces our earlier findings and highlights communication efficiency as the key advantage of dendritic neuron models.

#### Additional empirical verification

To further substantiate our findings, we conducted additional experiments using a diverse array of model architectures and datasets. This analysis included an assortment of models, including those lacking residual connections and those that leverage transformer-based architectures and one-dimensional (1D) convolutional networks for speech recognition. For the sake of clarity, we have included these additional results in Note S5. Similar conclusions are drawn from these supplementary experiments.

### Local communication cost analysis

Our analysis demonstrates that incorporating dendritic structures into neural networks significantly reduces the required neuron count (*D*) without sacrificing performance, provided the *D* is sufficiently large. In biological brains, fewer neurons correspond to reduced volume and connectivity, potentially decreasing both neuronal soma volume and the white matter, which consists predominantly of long-range axons and constitutes approximately 60% of the brain’s total volume.^27^ For ANNs running on hardware such as GPUs, reducing *D* cuts costs by limiting data transfers to off-chip memory and decreasing memory usage for hidden-layer activations during inference. We define this neuron-count-related cost *D* as the inter-layer communication cost.

Although dendritic architectures lower *D*, Figure 2 shows that dendritic neurons require more synaptic connections per input neuron to match point-neuron model complexity/performance. Thus, communication costs must also account for connecting these additional synapses (weights).

**Figure 4:**
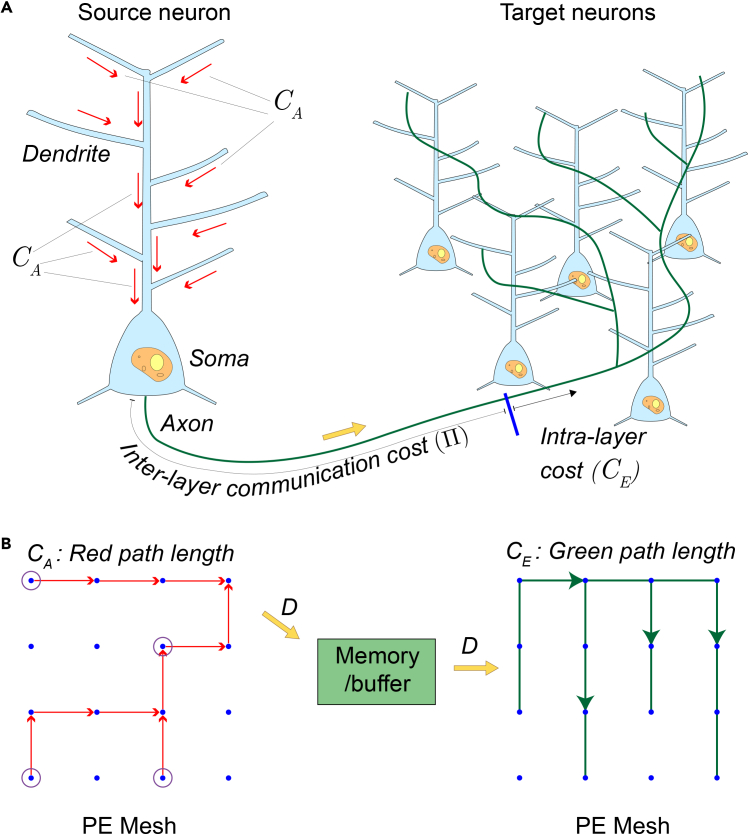
Illustration of communication cost metrics (*C*_*A*_, *D*, and *C*_*E*_) in biological and artificial neural networks (A) Biological neural networks. (B) ANNs. In this context, *C*_*A*_ represents the communication cost associated with aggregating synaptic inputs, *D* denotes the inter-layer communication cost, and *C*_*E*_ signifies the expense related to signal propagation to each synapse (weight). We measure *C*_*A*_ and *C*_*E*_ by the total path length over which the signal traverses. For the ANN models, we assume model inference is performed on a mesh of processing elements (PEs). Each blue dot represents one PE unit in the mesh. It is important to note that the division into these three metrics is not intended to be exact.

Figure 4 provides a breakdown of communication costs in both biological networks and ANNs, dividing costs into three parts: intra-neuron aggregation cost (*C*_*A*_), inter-layer communication cost (*D*), and intra-layer signal propagation cost (*C*_*E*_). Costs *C*_*A*_ and *C*_*E*_ are measured by the signal’s travel distance, while *D* reflects neuron count.

The top image of Figure 4 depicts the communication costs in biological neural networks. Here, *C*_*A*_ corresponds to the cost of aggregating outputs from the dendritic tree and sending them to the cell body. Due to the challenge of separating *C*_*A*_ from computation costs, this is not intended to be accurately defined. *D* represents the long-range communication cost, while *C*_*E*_ denotes the cost of axonal activation reaching the target synapses.

The bottom image in Figure 4 illustrates the same metrics for ANNs, assuming inference is performed on a mesh of processing elements (PEs). The left section shows aggregation costs (*C*_*A*_), the middle represents inter-layer costs (*D*, including memory storage considerations), and the right indicates intra-layer costs (*C*_*E*_), measured by path length between PEs.

Returning to Figure 2, we emphasize the importance of considering communication costs beyond inter-layer interactions. The point-neuron model receives and outputs data of dimension *D*. In contrast, the dendritic neuron model preserves complexity by using fewer neurons ($\hat{D}=D/\sqrt{K}$) but distributing inputs across more dendrites ($D\sqrt{K}$). In this case, *D* and $\hat{D}$ correspond to the number of neurons for the network layer. Although both model architectures process the same total number of inputs (*D*^2^), differences in neuron and dendrite configurations significantly influence communication costs—captured by *C*_*A*_, *C*_*E*_, and *D*—as detailed in the following analysis.

#### Cost estimation for a biological neuronal network

**Figure 5:**
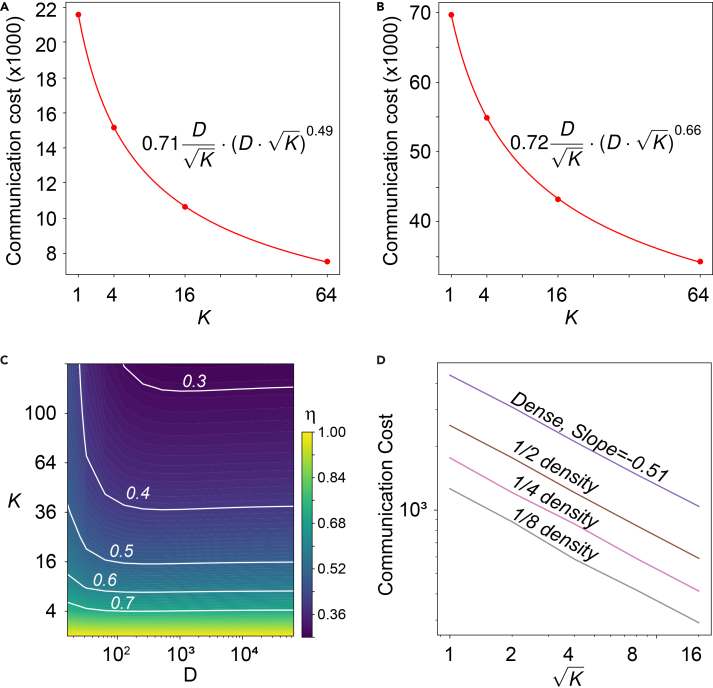
Estimation of signal propagation costs in biological and artificial neural network layers (A and B) Estimation of signal propagation costs *C*_*E*_ for a biological network layer with a varying number of dendrites per neuron (*K*) and a baseline network of dimension *D* = 1,024. Post-synaptic targets were sampled from (A) a unit square and (B) a unit cube. In each image, the curve and its corresponding equation are fitted to the data points. In the biological model (A/B), *C*_*E*_ is estimated via Euclidean minimal spanning trees (green path in A), while *C*_*A*_ (red path) is not estimated. (C and D) Estimation of signal propagation costs for an ANN layer. (C) Topographic representation of the ratio $\eta =\left({\hat{C}}_{A}+{\hat{C}}_{E}\right)/\left(C_{A}+C_{E}\right)$: the visualization highlights the influence of the variations in *D* and *K* on the *η*. (D) demonstrates the variations in ${\hat{C}}_{E}$ as a function of $\sqrt{K}$ and levels of connection sparsity. The axes are depicted on a logarithmic scale. When *K* = 1, the models are based on point neurons. For this experiment, a *D* value of 256 was utilized. The slope is obtained from fitting a line to the logarithm of *C*_*E*_ against the logarithm of $\sqrt{K}$.

We quantified the wiring (communication) costs, *C*_*E*_, required to connect $\hat{D}=D/\sqrt{K}$ input neurons to $D\cdot \sqrt{K}$ synapses each, resulting in a total of *D*^2^ synapses in biological neuronal networks. These costs were evaluated across various dendritic counts per neuron, *K* = {1,4,16,64}, assuming synapses are spatially distributed either in a two-dimensional (2D) plane or a three-dimensional (3D) volume. Both empirical measurements and fitting with theoretical predictions are presented in Figures 5A (2D case) and 5B (3D case). The special case *K* = 1 corresponds to the point-neuron model. Clearly, the results demonstrate a significant advantage in adopting dendritic neurons. Further methodological details are provided in the communication cost analysis portion of the methods section.

We do not attempt to estimate the impact of having dendritic neurons on *C*_*A*_ for biological neuronal networks due to the scarcity of biological data and difficulty in separating the computing cost from the aggregating communication cost. However, we speculate that the cost of signal aggregation in biological neurons will be mostly dependent on the number of inputs, and thus adopting a nonlinear or linear dendrite would not significantly affect this type of cost.

#### Cost estimation for an ANN

We analyzed the communication cost of ANNs using a simplified parallel architecture model (see Note S3 for the detailed mathematical derivation, including the PE grid definition and minimal rectilinear spanning tree [MRST] calculations). Our results show that incorporating dendritic neuron structures in ANN models can significantly reduce on-chip communication cost compared to traditional point-neuron models. Specifically, for fixed computational complexity, dendritic models consistently exhibit lower communication costs as the number of dendrites per neuron *K* increases.

Figure 5C shows that the ratio of communication costs *η* between dendritic-neuron- and point-neuron-based models decreases with increasing *K*. Moreover, as shown in Note S3, in most configurations, the communication cost for dendritic models is dominated by ${\hat{C}}_{E}$, especially for large input dimensions *D*, which is common in practice.

Further analysis reveals that ${\hat{C}}_{E}$ decreases with increasing model sparsity and increasing *K*, following a negative power-law relationship with $\sqrt{K}$, specifically ${\hat{C}}_{E}\propto K^{-0.51}$, as shown in Figure 5D. This closely matches the simplified theoretical form of ${\hat{C}}_{E}$ derived in Equation S9, which includes a *K*^1/4^ scaling factor. These findings suggest that dendritic neurons can effectively reduce communication costs in sparse ANNs.

### Reducing memory access cost during training and inference on a GPU

#### Model inference

We also extended our analysis to modern GPU-based architectures. Theoretical estimates indicate that dendritic neurons can reduce global memory access and improve efficiency by a factor of $\sqrt{K}$, highlighting their potential for improving performance in realistic parallel hardware settings. We also verify the theoretical result with empirical experiments. Further details are provided in Note S4.

#### Model training

We have demonstrated that dendritic architectures can reduce communication costs during model inference. Can they similarly decrease memory communication costs during training?

At first glance, dendritic models require storing more intermediate activations during training compared to standard models. For example, in Figure 2, a dendritic-neuron-based layer stores 16 + 4 = 20 intermediate activations, while a point-neuron-based layer only stores 8 activations. Counting each activation value once (as obtaining post-activation function values from pre-activation function values incurs minimal cost), this translates to 320 (dendritic neuron) versus 128 (point neuron) bits per layer using 16-bit floats.

However, memory costs can be significantly reduced by leveraging gradient properties of commonly used activation functions (rectified linear unit [ReLU], leaky ReLU, GELU [Gaussian error linear unit], etc.). Naively, backpropagation through dendritic neurons requires storing intermediate activations *h* and dendritic pre-activations ${\hat{d}}_{j}$ (see Equations 2 and 3). Examining the gradient computations for a dendritic network layer with input **x**, dendritic pre-activation $\hat{d}$, and neuron-layer output *h*,

(Equation 4) $$\frac{\partial h}{\partial w_{j}}=\sigma^{\prime }\left({\hat{d}}_{j}\right)\cdot x,\frac{\partial h}{\partial x}=\sum_{j=1}^{K}\sigma^{\prime }\left({\hat{d}}_{j}\right)\cdot w_{j}\text{.}$$

We observe that the gradient calculation here does not depend on $\hat{d}$ or *d* but on $\sigma^{\prime }\left({\hat{d}}_{j}\right)$. For ReLU, this derivative is binary (0 or 1), allowing representation with a single bit per dendrite. Since only *h* (not ${\hat{d}}_{j}$) is needed for gradient computations of the subsequent layer (in the forward direction), it suffices to store just one bit per dendrite. In the example shown in Figure 2, this reduces memory from 320 bits (16-bit floats) to only 16 + 4 × 16 = 80 bits, even less than the point-neuron-based model (128 bits). Similar reductions are achievable for related piecewise activation functions (e.g., Leaky ReLU, parametric ReLU, and ReLU6).

Non-piecewise activation functions (e.g., GELU, ELU [exponential linear unit], and SELU [scaled exponential linear unit]) may require slightly higher precision. However, their gradient values typically span limited ranges, possibly enabling efficient storage using only a few bits per dendrite (e.g., two bits). Given the robustness of neural network training to gradient noise,^28^ this approximation may be acceptable in practice. A detailed investigation is left for future work.

## Discussion

This work was inspired by biological neurons, which aggregate signals from dendrites into a single output at the soma. Dendrites integrate inputs nonlinearly through voltage-gated channels and receptors, so we designed neural network units that mimic these characteristics.

Treating dendrites as individual point neurons (as previously proposed^10^), the idea of aggregating neuronal outputs is common in deep learning, such as spatial pooling in convolutional networks^29^ and Maxout networks,^30^ to enhance performance and reduce computational complexity. Prior studies employing dendrite-inspired pooling strategies reported superior computational and discriminatory capabilities compared to linear integration methods.^13^^,^^18^^,^^21^

Also related is the mixture of expert architecture, which is widely used in current mainstream large language models,^31^ where information from multiple experts is combined. However, in such systems, the experts are typically not located in close proximity—sometimes they are even distributed across different machines. Unlike the inherently local nature of dendritic architectures, this setup requires costly long-distance communication for information integration. Could a dendritic architecture serve as a more efficient alternative? Further exploration is warranted.

Several related studies include Naud’s sparse neuron ensembles,^32^ Sezener’s dendritic gated network (DGN),^33^ and Iyer et al.’s incorporation of active dendrites into ANNs.^34^ Naud showed neuron ensembles efficiently communicate combined signals from multiple sources, albeit through a different mechanism. Sezener emphasized performance without exploring how dendrite count affects efficacy. Unlike Sezener, our study evaluates dendritic efficiency, communication costs, and complexity. Iyer et al. focused on shallow ANNs and dendrites’ role in continual learning, contrasting with our emphasis on dendritic efficiency in deeper networks.

We demonstrate that dendritic neurons substantially improve communication efficiency as network size scales. Typically, increasing network width scales neuron counts (*D*) with the square root of parameters, while depth requires linear scaling. Our results demonstrate dendritic architectures can significantly increase parametric complexity without increasing *D*. This substantially reduces inter-layer communication, lowering data-transfer costs within computing chips, and may have analogous biological benefits,^20^ although further exploration of biological wiring costs remains necessary.

Our analysis of dendritic architecture’s communication advantages considered only wire length, excluding wiring volume. Earlier research suggests dendrites confer significant volume savings.^9^ Accounting for wiring volume would likely enhance our architecture’s benefits, but due to limited biological data, this aspect is left for future research.

Also relevant to our study is the concept of small-world networks, which achieve communication efficiency through specific connectivity patterns.^35^^,^^36^ We instead focus on local dendritic nonlinearities to minimize communication costs.

Our findings carry theoretical and practical implications. Theoretically, dendritic architectures suggest widening networks by enhancing feature complexity rather than solely increasing inter-layer communication. Practically, dendritic models outperform point-neuron models under equal communication budgets, substantially reducing memory access, especially beneficial for large-batch inference. Our results predict that dendritic designs can reduce on-chip communication costs, potentially informing neural accelerator design.

## Conclusion and future works

Finally, our findings parallel evolutionary patterns in biological brains, where complex dendritic structures emerge in larger neural systems due to increased computational demands.^7^ Integrating dendritic neurons into artificial networks may thus reflect fundamental biological principles, offering insights for efficient and scalable neural network design.

Several avenues remain for future research. Our analysis of reduced memory costs from dendritic neural network training is limited to ANNs. Whether this benefit translates to biological dendritic neurons remains open, given the limited understanding of biological learning mechanisms, is deferred to future research.

We also lack a complete understanding of why dendritic channel sharing matches or exceeds conventional model performance. One plausible explanation involves interpreting dendritic pooling as low-rank approximations of large weight matrices. Further investigation is necessary.

Notably, our dendritic models apply a single nonlinear layer solely at dendrites. Preliminary results indicated minor performance gains when adding additional somatic nonlinearities, particularly with a large number of dendrites. However, this is not included here, as the primary focus of this study is efficiency rather than incremental performance improvements. Exploring diverse nonlinearities and advanced architectures in dendritic neurons remains an intriguing future work.

This study’s analysis of active dendrites relied on machine learning experiments employing a rate-based model. It is crucial to acknowledge that this methodological choice may introduce limitations, particularly when comparing the findings to those derived from spike-based models.

## Methods

### Datasets for machine learning experiments

The present study leverages three commonly used datasets, ImageNet, CIFAR-100, and LibriSpeech, for model training and evaluation. These datasets are commonly served as benchmarks in deep learning research.

#### ImageNet dataset

For this study, we use the ILSVRC 2012 subset of the ImageNet dataset, which comprises 1.2 million training images and 50,000 validation images from 1,000 categories.^37^ The images vary in size and are resized to a fixed resolution of 224 × 224 pixels for uniformity, per the standard ResNet procedure.^26^ Typical data augmentation techniques, such as random cropping, random horizontal flipping, and color jittering, were applied during training to enhance the model’s generalization ability.

#### CIFAR-100 dataset

The dataset comprises 60,000 32 × 32 color images in 100 classes, with 600 images in each class. There are 50,000 training images and 10,000 test images.^38^ Like the ImageNet data processing, we followed the typical data augmentation procedure.^26^

#### LibriSpeech dataset

The dataset is a publicly available English speech corpus for automatic speech recognition (ASR) training and evaluation from the LibriVox project’s audiobooks. It consists of 1,000 h of transcribed speech, divided into training, development, and testing subsets.^39^ The experiment utilizing this dataset can be found in Note S5.

#### Model architectures

In this study, we primarily used the ResNet-18 architecture as the baseline model. ResNet-18 is an 18-layer deep residual neural network, a seminal model proposed by He et al.^26^ The baseline configuration of ResNet-18 encapsulates an initial convolutional layer, followed by four residual blocks, each of which consists of two convolutional layers. This pattern constitutes the primary structure of our working model; in contrast to the original ResNet-18 model, our adapted architecture positions the shortcut connection after the ReLU activation function. This modification is imperative to ensure the compatibility of the dendritic structure with the model architecture.

For experiments on scaling up networks, we scaled up each network layer by the same designated factor, except for the input and output of the model. For models with dendritic neurons, we replaced neurons in the standard model with dendritic neurons with *K* dendrites as specified by the experiment setting, except for the input and output layers of the model. To maintain the uniform model complexity scaling throughout the model, we equip the input layer and the penultimate layer of the model with neurons of $\sqrt{K}$ instead of *K* dendrites. The same setting is also employed in experiments designed to compare models that share identical inter-layer communication costs.

For models trained on CIFAR-100, we observed training instability. Therefore, we clipped the gradient norm to 1.0 during model training. We also added an extra batch norm to each dendrite to improve model stability. This additional batch norm can be fused with the previous layer and thus will not add extra computation burden at the inference stage.

In addition to models based on the ResNet-18 architecture, we have corroborated our findings using a model devoid of shortcut connections. This strategy ensures that the benefits observed are not strictly confined to a particular architecture. The configuration of this model is delineated in Note S5, where the corresponding experimental outcomes can also be found.

Moreover, our experimentation extended to the transformer-based model. Within this model, the standard feedforward layers are substituted with network layers based on dendritic neurons. Comprehensive details pertaining to this modification can be found in Note S5.

### Model training

We trained all models with a cosine learning rate decay schedule and the SGD optimizer with a momentum of 0.9.

For ImageNet with dense ResNet models, the learning rate was initialized at 0.4 (instead of 0.1, to compensate for the batch size used for training), and models were trained for 120 epochs, including two warm-up epochs with a learning rate of 0.04. Weight decay was set to 1 × 10^−4^. A batch size of 1,024 was employed, and the training was distributed across 8 GPUs.

For ImageNet with sparse ResNet models, the models were trained for 200 epochs with an initial learning rate of 0.1 and 2 warm-up epochs at a learning rate of 0.01. The weight decay parameter was set to 1 × 10^−4^. To achieve a sparse ratio of 85%, we applied L1-unstructured global pruning in 5 rounds, conducted between epochs 40 and 140. Subsequently, the models were trained for an additional 60 epochs.

Finally, for CIFAR-100 models, we trained them for 200 epochs with a learning rate of 0.05, including two warm-up epochs at a learning rate of 0.005. A batch size of 64 was utilized, and the weight decay parameter was set to 5 × 10^−4^.

Our investigation emphasizes the comparative analysis of the performance of various models under identical training conditions, facilitating an equitable assessment of the distinct capabilities of each model. Consequently, all models within the comparison group undergo training with the same hyperparameters, barring the requisite architecture adjustments. Further details concerning the experiments can be found in the accompanying source code.

### Communication cost analysis

#### Biological neural network

We modeled a baseline network layer with *D* input and *D* output neurons, resulting in *D*^2^ synapses. For each input neuron, $D\sqrt{K}$ synaptic targets were randomly distributed within either a unit square (2D) or a unit cube (3D), where *K* represents the number of dendrites per neuron and is varied across *K* = 1, 4, 16, 64.

To estimate the wiring length (represented as the green path in Figure 4A), we computed the Euclidean minimal spanning tree over each synapse set using the method described by Steele et al.^40^ This was repeated 10 times to obtain an average total path length. The communication cost *C*_*E*_ was then calculated as the product of $D/\sqrt{K}$ and the mean path length. We note that we did not attempt to estimate the aggregation cost (*C*_*A*_, red path) for biological networks due to the complexity of separating computation from aggregation in wetware. Finally, we fitted the results to a function of the form $\alpha \cdot D/\sqrt{K}\cdot {\left(D\sqrt{K}\right)}^{\beta }$, allowing us to extract the scaling exponent *β* and compare it to theoretical expectations.

#### Artificial neural network

We adopted a simplified parallel explicit communication model (PECM), inspired by Dally et al.,^19^ to estimate data movement costs in ANN inference hardware. The model assumes that computation occurs on a 2D grid of processing elements (PEs), interconnected via an on-chip network (NoC) within a unit square. This abstraction captures essential features of neuromorphic and parallel architectures used in real-world ANN hardware.^41^^,^^42^^,^^43^

Communication costs were analyzed for both point-neuron- and dendritic-neuron-based models, with derivations provided in the Note S3. Briefly, we utilized a MRST algorithm on a 2D grid to determine the optimal path for data dissemination. The focus was on two key components: aggregation cost *C*_*A*_ and external communication cost *C*_*E*_, as well as their dendritic counterparts ${\hat{C}}_{A}$ and ${\hat{C}}_{E}$.

We evaluated the communication cost ratio *η* between dendritic neuron and point-neuron models across a range of parameters: different values of input dimensionality *D* for point neurons and varying numbers of dendrites per neuron *K* for dendritic neurons. The impact of sparsity was also assessed by analyzing ${\hat{C}}_{E}$ under varying sparsity levels and dendrite counts.

## Resource availability

### Lead contact

Requests for further information and resources should be directed to and will be fulfilled by the lead contact, Xundong Wu (wuxundong@gmail.com).

### Materials availability

This study did not generate new materials.

### Data and code availability

• Our source code is available at GitHub (https://github.com/motifMachine/Dendrite_communication/) and has been archived at Figshare.^44^

## Acknowledgments

This work was supported by the National Natural Science Foundation of China under grant no. 6207608, the NIH under grant no. 1R01MH124867-02, and the Stavros Niarchos Foundation (SNF) jointly with the Hellenic Foundation for Research and Innovation (HFRI) under the 5th Call of “Science and Society” Action “Always Strive for Excellence – Theodoros Papazoglou,” grant no. 28056. The authors thank De Ma, Shuicheng Yan, Qianbo Yin, and Mingyang Zhao for their insightful discussions and for sharing their expertise during the development of this work.

## Author contributions

Conceptualization, X.W., L.M., T.H., G.P., and H.T.; methodology, X.W., P.Z., Z.Y., and Y.G.; investigation, X.W., P.Z., Z.Y., L.M., K.-W.Y., P.P., T.H., G.P., and H.T.; visualization, X.W., K.-W.Y., and L.M.; writing—original draft, X.W.; writing—review & editing, X.W. and P.P.; resources, X.W., L.M., T.H., G.P., and H.T.

## Declaration of interests

X.W. and Y.G. are inventors on a patent related to this work issued to Zhejiang Lab. X.W., L.M., and T.H. are inventors on a patent related to this work issued to the Beijing Academy of Artificial Intelligence.

## Declaration of generative AI and AI-assisted technologies in the writing process

During the preparation of this work, the authors used ChatGPT in order to improve readability and language. After using this tool/service, the authors reviewed and edited the content as needed and take full responsibility for the content of the publication.

## Supplemental information

Document S1. Figures S1–S9, Tables S1–S3, and Notes S1–S5

Document S2. Article plus supplemental information
