# Supplementary material for: Dendritic nonlinearities mitigate communication costs
Source: Patterns (N Y). 2026 Mar 30;7(6):101520. doi: 10.1016/j.patter.2026.101520 (PMC13280677; doi:10.1016/j.patter.2026.101520)
Supplement: Document S1. Figures S1–S9, Tables S1–S3, and Notes S1–S5 [file mmc1.pdf]

Patterns, Volume *7*

Supplemental information

Dendritic nonlinearities mitigate communication costs

Xundong Wu, Pengfei Zhao, Zilin Yu, Lei Ma, Yifan Gao, Ka-Wa Yip, Huajin Tang, Gang Pan, Panayiota Poirazi, and Tiejun Huang

Supplemental Notes

S1 Proof of Theorem S1

As illustrated in Eq. S1, the left-hand term—representing the information from the pooled neuron output—is bounded by the information present in the dendrites being pooled. Following shows a comprehensive proof of this theorem.

Theorem S1. *The entropy of the sum (neuron output) of random variables (dendritic outputs)* $\mathsf{d}_1, \mathsf{d}_2, \ldots, \mathsf{d}_K$ *is less than or equal to the joint entropy of these random variables. The relation between the two is given as:*

$$H\left(\sum_{j=1}^{K} \mathsf{d}_j\right) = H(\mathsf{d}_1, \ldots, \mathsf{d}_K) - H\left(\mathsf{d}_1, \ldots, \mathsf{d}_K \mid \sum_{j=1}^{K} \mathsf{d}_j\right) . \tag{S1}$$

In order to prove Theorem S1, we first prove the following lemma.

Lemma S1. *The conditional entropy of* $\sum_{j=1}^{K} \mathsf{d}_j$ *given* $\mathsf{d}_1, \mathsf{d}_2, \ldots, \mathsf{d}_K$ *is zero, i.e.,*

$$H\left(\sum_{j=1}^{K} \mathsf{d}_j \mid \mathsf{d}_1, \mathsf{d}_2, \ldots, \mathsf{d}_K\right) = 0 . \tag{S2}$$

Proof. If the values of $\mathsf{d}_1, \mathsf{d}_2, \ldots, \mathsf{d}_K$ are known, then the value of $\sum_{j=1}^{K} \mathsf{d}_j$ is also known. Therefore, the statement is intuitively true. For discrete random variables d_i, a formal proof can be presented as follows.

$$\begin{aligned} & H\left(\sum_{j=1}^{K} \mathsf{d}_j \mid \mathsf{d}_1, \mathsf{d}_2, \ldots, \mathsf{d}_K\right) \\ &= \sum_{d_1, \ldots, d_K} p(d_1, \ldots, d_K) H\left(\sum_{j=1}^{K} \mathsf{d}_j \mid \mathsf{d}_1 = d_1, \ldots, \mathsf{d}_K = d_K\right) \\ &= \sum_{d} 0 = 0 . \end{aligned} \tag{S3}$$

□

We can now prove Theorem S1, by first making use of a relationship between joint entropy and conditional entropy[S1].

Proof of Theorem S1: The joint entropy of $\mathsf{d}_1, \mathsf{d}_2, \ldots, \mathsf{d}_K$ and $\sum_{j=1}^{K} \mathsf{d}_j$ is:

$$\begin{aligned} & H\left(\mathsf{d}_1, \ldots, \mathsf{d}_K, \sum_{j=1}^{K} \mathsf{d}_j\right) \\ &= H\left(\sum_{j=1}^{K} \mathsf{d}_j\right) + H\left(\mathsf{d}_1, \ldots, \mathsf{d}_K \mid \sum_{j=1}^{K} \mathsf{d}_j\right) \\ &= H(\mathsf{d}_1, \ldots, \mathsf{d}_K) + H\left(\sum_{j=1}^{K} \mathsf{d}_j \mid \mathsf{d}_1, \ldots, \mathsf{d}_K\right) . \end{aligned} \tag{S4}$$

Therefore,

$$
\begin{aligned}
&H\left(\sum_{j=1}^{K}\mathsf{d}_j\right)\\
&= H(\mathsf{d}_1,\ldots,\mathsf{d}_K) + H\left(\sum_{j=1}^{K}\mathsf{d}_j \mid \mathsf{d}_1,\ldots,\mathsf{d}_K\right) - H\left(\mathsf{d}_1,\ldots,\mathsf{d}_K \mid \sum_{j=1}^{K}\mathsf{d}_j\right)\\
&= H(\mathsf{d}_1,\ldots,\mathsf{d}_K) - H\left(\mathsf{d}_1,\ldots,\mathsf{d}_K \mid \sum_{j=1}^{K}\mathsf{d}_j\right) \qquad (\textit{From Lemma S1.}) \qquad \text{(S5)}
\end{aligned}
$$

□

Since the conditional entropy $H\left(\mathsf{d}_1,\ldots,\mathsf{d}_K \mid \sum_{j=1}^{K}\mathsf{d}_j\right)$ is non-negative, the upper bound of $H\left(\sum_{j=1}^{K}\mathsf{d}_j\right)$ is $H(\mathsf{d}_1,\mathsf{d}_2,\ldots,\mathsf{d}_K)$.

S2 Computing and parametric complexity of models

Table S1: Complexity data on typical models

Dendrites/neuron (K)	$\Psi = 1/\sqrt{K}$	Computing complexity (MMACs)	# of Parameters
1 (Resnet-18)	1	1,821.63	11,689,512
4	1/2	1,804.34	11,556,200
16	1/4	1,799.65	11,521,800
64	1/16	1,799.37	11,512,664

Table S1 shows the computing and parametric complexity comparison of models from the light-blue dashed curve of Fig. 3. We use customized THOP package to calculate the model complexity data where we count two sum operations as one MAC operation.

S3 Derivation of Communication Costs for PE Mesh Architecture

S3.1 Point neurons based model

Our analysis initiates with a model composed of point neurons. As previously mentioned, our investigation focuses on two network layers. We assume that the first layer sends an output of D dimensions to the second layer. For convenience and without loss of generality, we assume that each of the D dimensions originates from one PE on the chip.

In order to arrange D PEs on die area of size 1×1, each PE must have a height and width of $l = 1/\sqrt{D}$, resulting in an area size of $1/D$. Similarly, the second layer is also composed of D PEs of the same size. Consequently, we obtain a grid of N by N PEs with $N = \sqrt{D}$, with a distance of l between the center of each pair of neighboring PEs. See Fig. S1-A for a visual illustration.

For this arrangement we have

$$C_A = D(\sqrt{D}-1)l = D - \sqrt{D}\,, \qquad \text{(S6)}$$

as measured with Manhattan distance. Furthermore, an illustrative example of signal propagation within this context is provided in Fig. S1-B. Derivation of Eq. S6 can be found in Supplemental S3.3.

We assess C_E with minimal rectilinear spanning tree (MRST) algorithm[S2]. Given a grid of $N\times N$ PEs, the objective is to deliver every dimension of the data to each PE. The MRST algorithm enables us to determine the minimal path length required to connect all PEs, which is $(N^2-1)\cdot l$. An example path is illustrated in Fig. S1-C.

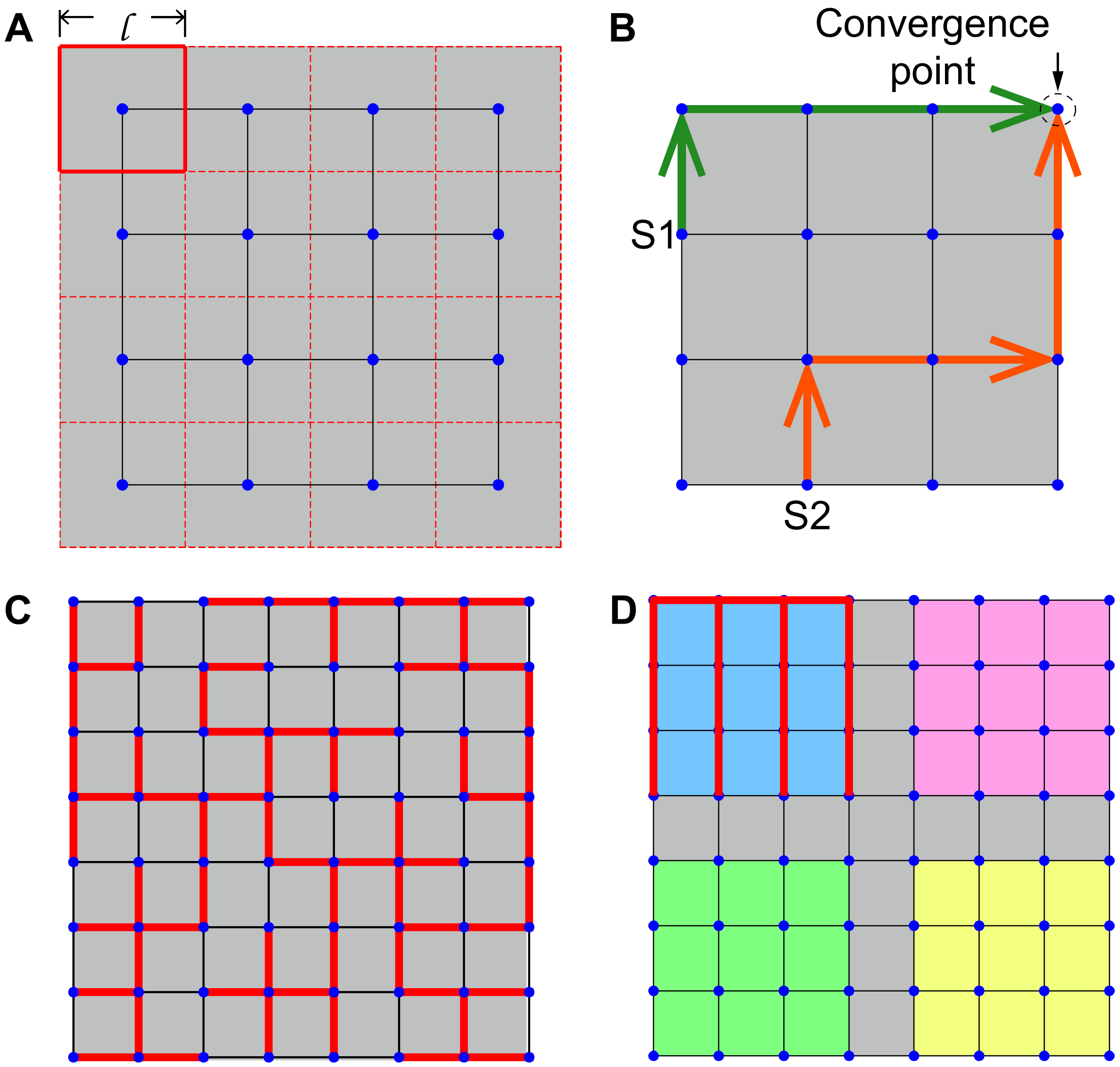

Figure S1: Illustration of the Processing Element (PE) Grid and Communication Path Models for Point and Dendritic Neurons.
(A) Showcases a 16-unit grid of processing elements (PEs), where each PE has a side length, l, computed as $l = 1/\sqrt{D}$ or $1/4$ in this example. The boundary of top-left PE is emphasized with solid red lines. For improved clarity, only the grid of central points will be displayed henceforth.(B) Depicts two city-walk paths originating from S1 (green path) and S2 (orange path) leading to a convergence point. The green path has a total length of $4l$, while the red path spans $5l$ in length. (C) Demonstrates a city-walk path with red lines, connecting all points on an 8×8 grid. This route enables data dissemination across the target set with minimal cost. (D) Illustrates four groups of PEs, each color-coded to represent a dendritic neuron with 16 dendrites. Within each group, dendritic outputs are combined to generate a single output. The aggregation path can be assessed using the MRST algorithm, with an example path displayed in the top-left block.

Consequently, we obtain the cost of delivering data as

$$C_E = (N^2 - 1) \cdot l \cdot D = (D - 1)\sqrt{D}\,. \tag{S7}$$

S3.2 Dendritic neuron based model

As earlier we maintain the number of parameters and floating-point operations (FLOPs) consistent with those in the point neuron model scenario. That is, given that each neuron has K dendrites, one layer of the model under examination will have a total of $M = D\sqrt{K}$ dendrites. As illustrated in Fig. S1-D, every group of K dendrites aggregates to form a single output dimension. Consequently, the first layer will produce an output with a dimensionality of $\hat{D} = D/\sqrt{K}$, which serves to maintain an equivalent computational complexity as the point neuron-based model previously described. We reiterate our assumption that those $\hat{D}$ neurons are arranged in a grid format, specifically of size $\hat{N} \times \hat{N}$, with $\hat{N} = \sqrt{\hat{D}}$.

We postulate that the computation of each dendrite is processed by one PE. In this scenario, the die area is divided into M units, with each unit occupying a specific area. The height and width of this area, denoted by $\hat{l}$, can be calculated as $\hat{l} = 1/\sqrt{M}$. Through this, we arrive at the size of a PE for processing each dendrite being $\frac{1}{D\sqrt{K}}$, which is $1/\sqrt{K}$ of the point neuron-based model PE die size. This corresponds to the assumption that a dendrite in this analysis receives a proportion of $1/\sqrt{K}$ of the inputs that a point neuron receives.

In light of the aforementioned derivation, we note that the signal transfer cost, denoted as $\hat{C}_A$, consists of two components. The first component, $\hat{C}_{AG}$, refers to the cost of aggregating dendritic outputs for each neuron. The second component, $\hat{C}_{AA}$, represents the cost of transmitting the aggregated data of all neurons off the die. Their expressions are as follows.

$$\hat{C}_A = \hat{C}_{AG} + \hat{C}_{AA} < \sqrt{D}K^{1/4} + \frac{D}{\sqrt{K}} \,. \tag{S8}$$

Please see S3.4 for the derivation.

In congruence with the approach adopted for dense models, we also employ the MRST algorithm to estimate the communication cost when dealing with sparse models. Considering the variability in the communication cost due to different sparse connection patterns, we sample a set of 100 random connection patterns for each setting to provide a robust estimate of the average cost. Akin to the point neuron models, we will not attempt to derive $\hat{C}_I$, although we have the relationship of $D = \sqrt{K} \cdot \hat{C}_I$ under the assumptions of the equivalent parameter/FLOPs count setting.

As for the $\hat{C}_E$ component, note that the second layer receives $\frac{D}{\sqrt{K}}$ inputs and consists of M units. Utilizing the MRST method, the cost associated with one-dimensional input connecting to M units can be computed as $(M-1)\cdot\hat{l}$. We arrive at

$$\hat{C}_E = \frac{D}{\sqrt{K}}(D\sqrt{K} - 1) \cdot \hat{l} \approx D^{\frac{3}{2}}/K^{\frac{1}{4}} \,. \tag{S9}$$

S3.3 Derivation of Eq. S6

For simplicity, we place the inter-chip communication junction point at the top-right corner, in the 0-th row and column. It starts with ID 0, counting from right to left and top to bottom. Therefore, the total cost of propagating outputs from every PE to the junction point is:

$$\begin{aligned}
C_A &= \left(\sum_{x,y=0}^{N-1}(x+y)\right) l \\
&= \left(\sum_{x=0}^{N-1} x \sum_{y=0}^{N-1} 1 + \sum_{x=0}^{N-1} 1 \sum_{y=0}^{N-1} y\right) l \\
&= \left(\frac{(N-1)N}{2}N + N\frac{(N-1)N}{2}\right) l \\
&= N^2(N-1)l \\
&= D(\sqrt{D}-1)\frac{1}{\sqrt{D}} \\
&= D - \sqrt{D} \,.
\end{aligned} \tag{S10}$$

S3.4 Derivation of Eq. S8

$$\begin{aligned}\hat{C}_{AG} &= (K-1)\cdot\hat{D}\cdot\hat{l}\\ &= \sqrt{D}(K^{1/4}-K^{-3/4}) < \sqrt{D}K^{1/4}\,, \end{aligned} \tag{S11}$$

$$\hat{C}_{AA} = \hat{N}\hat{N}(\hat{N}-1)\hat{l}(\sqrt{K}) < \frac{D}{\sqrt{K}}\,, \tag{S12}$$

$$\hat{C}_{A} = \hat{C}_{AG} + \hat{C}_{AA} < \sqrt{D}K^{1/4} + \frac{D}{\sqrt{K}}\,. \tag{S13}$$

S4 Communication cost Analysis for block-wise GEMM computation on GPU

S4.1 Theoretical analysis

In this section, we analyze how the adoption of the proposed dendritic structure affects communication costs during neural network inference when compared to a point neuron-based structure on typical GPU-like architectures.

For this part of analysis we follow the notation used by the GPU community as in CUTLASS[S3], which differs from the notation used in the rest of the manuscript.

First, we delineate our setting, assuming a feed-forward network layer. For the standard model, the computation of the layer can be expressed as

$$\boldsymbol{C}^f = \sigma(\boldsymbol{A}\cdot\boldsymbol{B}),$$

where σ represents the element-wise nonlinear output function. For clarity, we omit the bias term.

The computational complexity of the nonlinear function σ is relatively small compared to that of the matrix multiplication. Therefore, our focus will be on the matrix multiplication

$$\boldsymbol{C} = \boldsymbol{A}\cdot\boldsymbol{B}.$$

And we have $\boldsymbol{A}\in\mathbb{R}^{M\times L}$, $\boldsymbol{B}\in\mathbb{R}^{L\times N}$, $\boldsymbol{C}\in\mathbb{R}^{M\times N}$.

Similarly, for the dendritic model, we have

$$\hat{\boldsymbol{C}}^f = \sigma(\hat{\boldsymbol{A}}\cdot\hat{\boldsymbol{B}})$$

before the dendritic aggregation process. The dendritic layer output $\hat{\boldsymbol{C}}^o$ is then computed as

$$\hat{C}^o_{i,j} = \sum_{s=1}^{K}\hat{C}_{i,(j-1)K+s}$$

, where K is the number of dendrites per neuron. We have $\hat{\boldsymbol{A}}\in\mathbb{R}^{M\times L/\sqrt{K}}$, $\hat{\boldsymbol{B}}\in\mathbb{R}^{L\times N\sqrt{K}}$, $\hat{\boldsymbol{C}}\in\mathbb{R}^{M\times N\sqrt{K}}$. As described above, we reduce every K neighboring elements along N dimensions in $\hat{\boldsymbol{C}}^f$ into one element, namely the output of the dendritic layer $\hat{\boldsymbol{C}}^o$. Given that dendritic aggregation can be performed locally with low cost and the computing complexity of nonlinear functions is small. We again focus on the core matrix multiplication part described as $\hat{\boldsymbol{C}} = \hat{\boldsymbol{A}}\cdot\hat{\boldsymbol{B}}$. Though the aggregation process is important for reducing output communication cost.

To understand the communication cost we need to get some idea about the memory hierarchy of a GPU. The architecture depicted in the Fig. S2 represents a simplified illustration of a typical GPU processor such as Nvidia A100/H100. In this architecture, the global memory is a central storage resource accessible to all processing elements (PEs) within the processor. The PE units here correspond to Streaming Multiprocessors (SMs) of GPUs. To reduce the communication cost of accessing the high latency global memory, there is also an on-chip L2 cache that accelerates data I/O of PEs. Each PE is equipped with its own private shared memory, which is utilized by the tensor cores housed within that PE. Each tensor core within a PE is further equipped with its own private registers for localized computing, ensuring rapid access to frequently used data and further optimizing computational efficiency.

To avoid diving too deep into GEMM optimization, we refer readers to the CUTLASS documentation for introduction on block-wise general matrix multiply (GEMM)[S3].

The pseudo code for the baseline GEMM is shown in Alg. 1. We first calculate communication complexity of the baseline models without considering L2 cache.

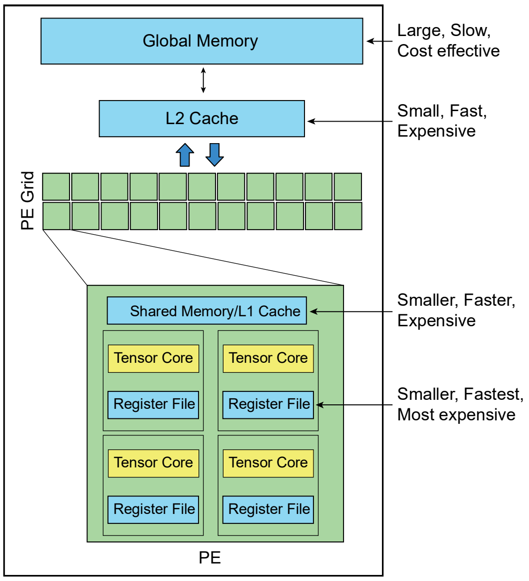

Figure S2: Simplified architecture of a GPU processor. The global memory, characterized by being large, slower, and cost-effective, is accessible by all processing elements (PEs) in the processor[S3,S4]. On chip L2 cache enable much faster access to data accessed by PE units. Each PE contains its own private shared memory, which is smaller, faster, and more costly, shared by the tensor cores within that PE. Each tensor core has its own private register file, which is the smallest, fastest, and associated with low communication cost.

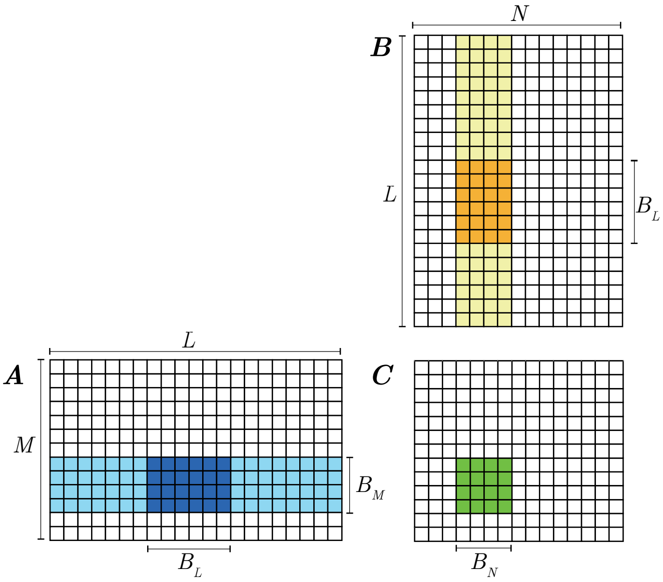

Figure S3: Block-wise General Matrix-Matrix Multiplication (GEMM) illustration.
This figure demonstrates the multiplication of matrices $\boldsymbol{C} = \boldsymbol{A} \cdot \boldsymbol{B}$, where $\boldsymbol{A} \in \mathbb{R}^{M \times L}$, $\boldsymbol{B} \in \mathbb{R}^{L \times N}$, and $\boldsymbol{C} \in \mathbb{R}^{M \times N}$. The computation is divided into blocks to optimize performance and efficiency.

From Fig. S3, for each block in $\boldsymbol{C}$ of size B_M by B_N, we need to read $L(B_M + B_N)$ units of data from global memory. Therefore for computation of the whole $\boldsymbol{C}$ we need to read

$$(B_M + B_N) \cdot L \cdot \frac{M}{B_M} \cdot \frac{N}{B_N}$$

units of data. To write the result matrix $\boldsymbol{C}$ to the global memory, the write cost is

$$M \cdot N.$$

For a dendritic model with K dendrites per neuron, we have $\hat{\boldsymbol{A}} \in \mathbb{R}^{M\times L/\sqrt{K}}$, $\hat{\boldsymbol{B}} \in \mathbb{R}^{L\times N\sqrt{K}}$, $\hat{\boldsymbol{C}} \in \mathbb{R}^{M\times N\sqrt{K}}$. With this new matrix dimensionality we have the following memory read cost for the dendritic model:

$$(B_M + B_N) \cdot \frac{L}{\sqrt{K}} \cdot \frac{M}{B_M} \cdot \frac{N \cdot \sqrt{K}}{B_N} = (B_M + B_N) \cdot L \cdot \frac{M}{B_M} \cdot \frac{N}{B_N}.$$

This is the same as the point neuron based model. As for the write cost, because we reduce elements in $\hat{\boldsymbol{C}}$ by group of K, we have a writing cost of :

$$\frac{M \cdot N}{\sqrt{K}}.$$

From the above analysis, it is evident that with the same B_M, B_N combinations for equivalent point neuron and dendritic neuron based network layers, there is no difference in the total memory read cost. The memory write cost can be reduced by $\sqrt{K}$. This analysis may suggest that adopting dendritic structure can only lead to minor reduction in communication cost given memory read is much larger than memory write.

The key is to coordinate block processing properly to take advantage of the L2 cache. For this part we refer the reader to the "L2 cache optimization" section of the Triton GEMM tutorial[S5] and CUTLASS documentation[S3] for background information.

To improve computational efficiency, it is beneficial to take advantage of the sharing of data among neighboring blocks of the matrix $\boldsymbol{C}$. This is achieved by computing several adjacent rows of $\boldsymbol{C}$, which correspond to the same rows in the matrix $\boldsymbol{A}$, as a group. This grouped computation strategy allows for the reuse of input blocks from matrices $\boldsymbol{A}$ and $\boldsymbol{B}$, minimizing data reloading and maximizing cache utilization. After completing the computation for one group, the process then transitions to another group. This approach not only streamlines data access patterns but also significantly reduces memory overhead and improves overall performance of GEMM computation.

Here we provide a simplified analysis on how dendritic architecture can help improve L2 cache hit rate therefore reduce communication cost on global memory access. Due to the complexity of the hierarchy cache mechanism, this analysis is not intended to be precise, but rather to help provide a theoretical understanding.

Assume that we form a block group of G rows of blocks from $\boldsymbol{A}$ according to the standard approach to improve the efficiency of L2 cache[S3,S5]. For this to work, we need to fit the block of $G \cdot B_M$ rows and a single B_N column into the L2 cache. We denote the capacity of the L2 cache as Q. That is, we have

$$(G \cdot B_M + B_N) \cdot L = Q.$$

It is possible to put multiple columns from the $\boldsymbol{B}$ matrix in the cache, but we can consider that it is absorbed in B_N. To utilize the cache efficiently, $G \cdot B_M$ need to stay in the cache while computations are performed along the N axis[S5] where each step of computation requires read B_N columns from the memory. In this way we can calculate that the memory read cost on matrix $\boldsymbol{B}$ part is

$$\frac{N \cdot L \cdot M}{B_M \cdot G}.$$

And the read cost on $\boldsymbol{A}$ part is $M \cdot L$, the total read cost would be

$$\frac{N \cdot L \cdot M}{Q/L - B_N} + M \cdot L.$$

It is desirable to set B_N to a small value. Therefore the read cost will roughly be equal to

$$\frac{N \cdot L^2 \cdot M}{Q} + M \cdot L.$$

For models with dendritic neurons of K dendrites, we will have a read cost of

$$\frac{N \cdot L^2 \cdot M}{(Q\sqrt{K})} + M \cdot L/\sqrt{K} = (\frac{N \cdot L^2 \cdot M}{Q} + M \cdot L)/\sqrt{K}.$$

Therefore, we can significantly reduce global memory read access through adopting dendritic structure.

Algorithm 1 Block-wise GEMM (Modified from the original algorithm from[S5])

Input: Matrices $\boldsymbol{A} \in \mathbb{R}^{M\times L}$, $\boldsymbol{B} \in \mathbb{R}^{L\times N}$, $\boldsymbol{C} \in \mathbb{R}^{M\times N}$
Output: Matrix $\boldsymbol{C}$ containing the result of $\boldsymbol{C} = \boldsymbol{A} \times \boldsymbol{B}$
Define: Block sizes B_M, B_N, B_L
for each m in 0 to M by B_M **do** ▷ Parallel execution over blocks of C
  for each n in 0 to N by B_N **do** ▷ Parallel execution over blocks of C
    Initialize $acc \leftarrow$ zeros(B_M, B_N)
    for each l in 0 to L by B_L **do** ▷ Iterate over blocks of A and B
      $a\_block \leftarrow \boldsymbol{A}[m : m + B_M, l : l + B_L]$
      $b\_block \leftarrow \boldsymbol{B}[l : l + B_L, n : n + B_N]$
      $acc \leftarrow acc + (a\_block \times b\_block)$
    end for
    $\boldsymbol{C}[m : m + B_M, n : n + B_N] \leftarrow acc$
  end for
end for

S4.2 Empirical analysis

The theoretical analysis presented above incorporates certain assumptions, such as a two-layer memory structure and explicit cache control, that do not fully align with the architecture of real-world hardware. To validate and extend this analysis, we conducted an empirical study of memory access costs during the inference process of typical neural network layers on an Nvidia A40 GPU. The results demonstrate that adopting a dendritic structure can significantly reduce communication costs, in alignment with the predictions of our theoretical framework. Due to the infeasibility of exploring the entire configuration space, this study does not aim to identify the optimal configuration for a specific setting. Instead, it aims to demonstrate clear advantages over well-established baselines that achieve the theoretical lower bound[S5–S7].

We measured the global memory access costs of dendritic neural network layers at three levels of computational complexity. Each baseline configuration used $K = 1$ (one dendrite per neuron), corresponding to a standard feedforward neural network with ReLU nonlinearity. The baseline implementation was built using the Triton standard framework[S3,S5], designed to optimize memory access costs while achieving performance comparable to CuBLAS. In this scenario, computation is represented as $C = \sigma(A \times B)$, where $A \in \mathbb{R}^{M\times L}$, $B \in \mathbb{R}^{L\times N}$, and $C \in \mathbb{R}^{M\times N}$, with σ denoting an element-wise nonlinearity. For all baseline experiments, $M = N = L$ was set to values from $\{1024, 2048, 4096, 8192\}$, representing the four complexity levels. We compared configurations with $K = 1, 4, 16, 64$ dendrites per neuron at each complexity level, ensuring that computational complexity remained consistent across baselines. For instance, at a complexity level of $M = 1024$ and $K = 4$, we set $M = 1024$, $L = 512$, and $N = 2048$. In this case, the final output matrix would have dimensions $\mathbb{R}^{1024\times 512}$ after aggregating the outputs from every four dendrites.

We analyze global memory access patterns using Nvidia Nsight Compute and report the optimal results for each tested configuration. The best results were obtained by searching over $B_M, B_N, B_L \in 16, 32, 64, 128$ (values compatible with the Tensor Core architecture) and $G \in 1, 2, 4, 8, 16, 32, 64, 128, 256$.

Our findings indicate that the dendritic structure offers notable advantages. For smaller layers ($M = 1024$, Fig. S4A), the operator matrices can fit within the L2 cache, leading to lower performance gains from the dendritic structure compared to theoretical predictions (red dashed lines). However, as the matrix size surpasses the L2 cache capacity ($M = 4096$, Fig. S4B), memory access patterns begin to align with the expected $1/\sqrt{K}$ scaling. For reference, the A40 GPU used in these experiments has an L2 cache size of 6,144 KB, as documented in Nvidia Ampere GA102 GPU Architecture white paper. For the largest matrices ($M = 8192$, Fig. S4D), cache evictions lead to memory reads exceeding theoretical predictions.

Similar trends are observed for the write costs, as shown in the middle row of Fig. S4. Note that, in the case of small output matrices, the result matrices may remain in the L2 cache without being transferred to global memory. This can lead to observed write costs smaller than the size of the output matrix. To show a complete picture we also show the optimal total global memory access cost (read+write) measured across different settings shown at the bottom row of Fig. S4.

The observations outlined above highlight a pathway to significantly reduce model inference energy costs by mitigating global memory access complexity. This approach could also facilitate GPU designs with lower memory bandwidth requirements. While reducing communication overhead lowers energy consumption, it does not always

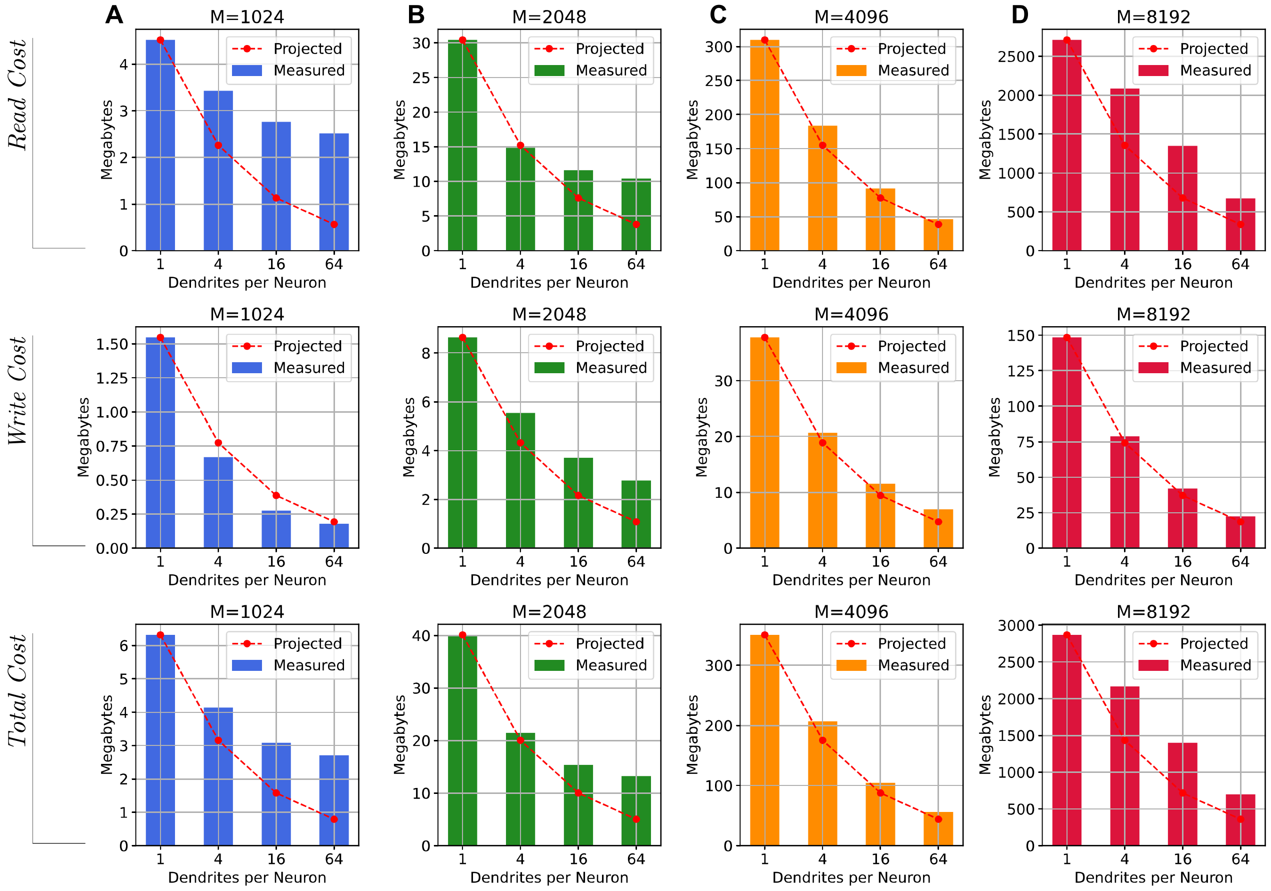

Figure S4: Communication cost analysis across neural network configurations. **Top row:** Optimal read costs for networks with varying dendrites per neuron ($K = 1, 4, 16, 64$) at three complexity levels: $M = 1024$ (A), $M = 2048$ (B), $M = 4096$ (C), and $M = 8192$ (D). Bar plots show measured costs, while dotted red lines indicate theoretical scaling projections. **Middle row:** Optimal write costs for the same configurations, showing measured write costs and their theoretical scaling predictions. **Bottom row:** Optimal total communication costs, combining both read and write operations for each configuration.

translate to accelerated model inference due to potential bottlenecks elsewhere in the pipeline. Notably, however, a significant relative performance advantage in network layer inference is observed when the GPU memory is underclocked. In this scenario, where memory I/O emerges as the primary bottleneck, the dendritic model exhibits substantial runtime reductions relative to the baseline, as shown in Fig. S5.

S5 Additional machine learning experimental analysis

S5.1 CIFAR-100 dataset with ResNet-18-style models

We also applied our models to the CIFAR-100 dataset[S8], which comprises 100 distinct object categories and is commonly employed in machine learning studies. Results are shown in Fig. S6 and resemble our findings using the ImageNet dataset, where incorporating dendrites into a model with a fixed inter-layer communication budget consistently yields improved performance. Furthermore, dendritic-based models surpass point-neuron models with the same computational budget, provided that the inter-layer communication budget is above a certain threshold. As in the ImageNet dataset, we used the ResNet-18 model as the baseline architecture.

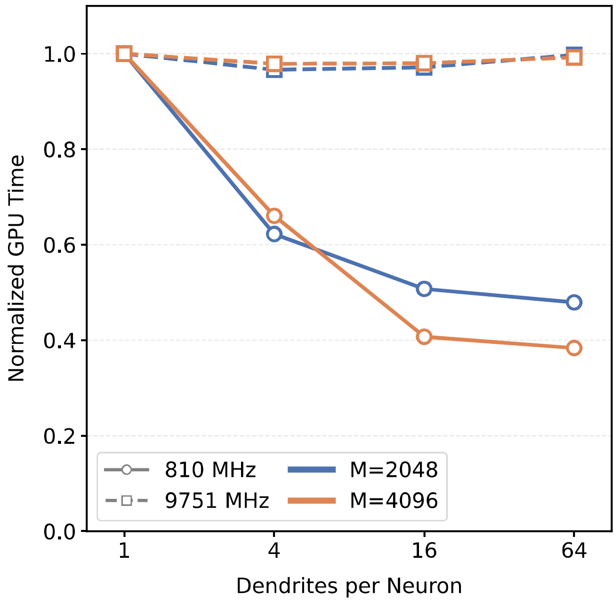

Figure S5: Normalized GPU runtime across varying computational complexities and GPU memory clock speeds. We set baseline $M = N = L$ to values of 2048 and 4096 for these experiments, with the number of dendrites per neuron K varying across $\{1, 4, 16, 64\}$. The GPU runtime is normalized relative to the baseline case for each computational complexity level and clock setting. These experiments were performed on an NVIDIA RTX 3090 GPU, which permits manual adjustment of the memory clock. For this set of experiments, we set the GPU core clock at 1935 MHz and tested memory clock speeds of 810 MHz (memory-constrained) and 9751 MHz (standard), respectively.

S5.2 Additional results on Imagenet dataset experiment

In the results section, we compare the performance of models when they are set to be of same computational complexity level. To obtain a full picture, we also compare models of same number of neurons D. The result is illustrated in Fig. S7. We can observe consistent performance improvement when more dendrites are added to the neurons.

S5.3 Non-Residual Convolutional Neural Network Performance on the ImageNet Dataset

To ensure the robustness of our findings, we also used a convolutional neural network (CNN) model devoid of residual connections[S9]. The base model for this experiment was a modified version of the original ResNet-18 network, from which we eliminated the residual connections. The original ResNet-18 model consists of four stages, each featuring two residual blocks. We removed one residual block from both the second and third stages to reduce computing costs. Fig. S8 illustrates the results from this modified, non-residual network, which are consistent with our original findings shown in Fig. 3.

S5.4 Transformer model

This section investigates the impact of replacing the feedforward block within transformer-based neural networks. The specific feedforward block in question comprises a classic bottleneck architecture, as illustrated in Fig. S9.

A bottleneck structure enhances the expressive capacity of a network module by expanding the number of channels in the middle layer. Conventionally, if the module input consists of L channels, the middle layer is expanded to comprise sL channels. Small integer values are commonly employed for s in typical transformer-based models, with common choices including 2, 3, or 4. Subsequently, the module's output is reduced back to the original L channels.

This bottleneck module confers greater expressivity power to the model than a standard two-layer network of L channels while maintaining a modest input/output channel number for the module. This is similar to what dendritic

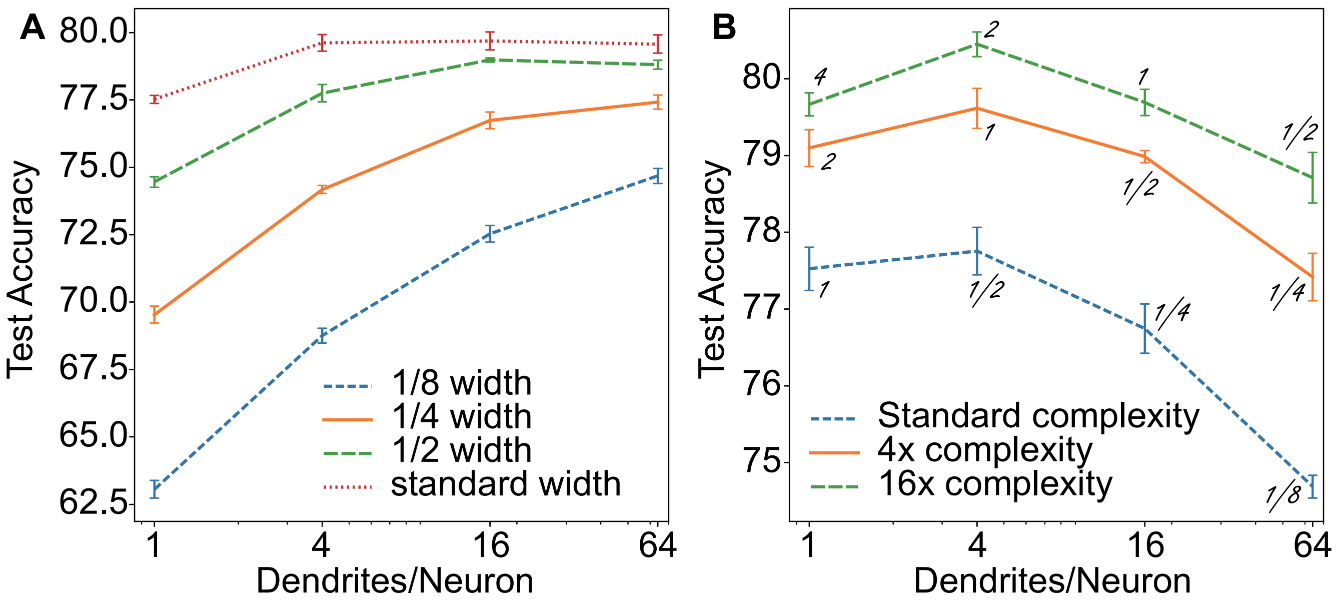

Figure S6: Results on the CIFAR-100 dataset.
Each experiment was performed 5 times, with standard deviations displayed. (A) Test accuracy for models with varying numbers of dendrites per neuron at four distinct levels of network width. (B) Comparison of models with equivalent computational complexities at three different levels. The blue dashed curves represent the baseline ResNet-18 model and subsequent dendritic models with K values of 4, 16, and 64. The orange curve corresponds to models with twice the number of neurons (channels), and the green dashed curve represents models with four times the number of channels. The channel scale factors relative to the standard model are labeled on the curves in (B).

structures try to achieve.

However, the bottleneck structure has an expanded middle layer, necessitating high communication bandwidth. Thus, the question arises: can a dendritic structure supplant the bottleneck structure while conferring additional benefits?

The naive substitution of a bottleneck structure with two dendritic layers is ineffective because the second layer comprises linear neurons. The pooling of linear neuron outputs does not confer inherent advantages to a nonlinear dendritic structure. Consequently, our design only employs a dendritic structure exclusively for the first layer of the block while retaining a linear layer for the second.

More precisely, for a bottleneck structure accepting an input dimension of L and an expansion ratio of s, the corresponding first layer is assigned the dendritic branches equal to $2s - 1$. This configuration maintains the input channel number for both layers at L, preserving the computational and parametric complexity at levels comparable to the original model.

An empirical examination involving a compact transformer model, as proposed by Hassani et al.[S10], demonstrates that this modification incurs only a marginal performance decline. Specifically, test accuracy on the ImageNet dataset decreased from 80.9% to 80.6%, a negligible reduction considering the substantial decrease in peak activation output I/O within the block threefold less than before.

Considering the highly tuned nature of the transformer architecture, we posit that additional refinements to the model—particularly adjustments favoring the dendritic structure may unlock further potential for performance enhancement.

S5.5 Speech recognition task

In addition, we substantiate our theory with a speech recognition task. We employ models trained on the LibriSpeech dataset, which consists of approximately 1,000 hours of spoken English[S11]. Owing to computing resource constraints, we utilize the train-clean-100 and train-clean-360 subsets for model training and the dev-clean subset for model evaluation. The models used in this portion of the experiment are derived from the Jasper model[S12], a 1D convolutional neural network. To lessen the computational burden during model training, we modified the original

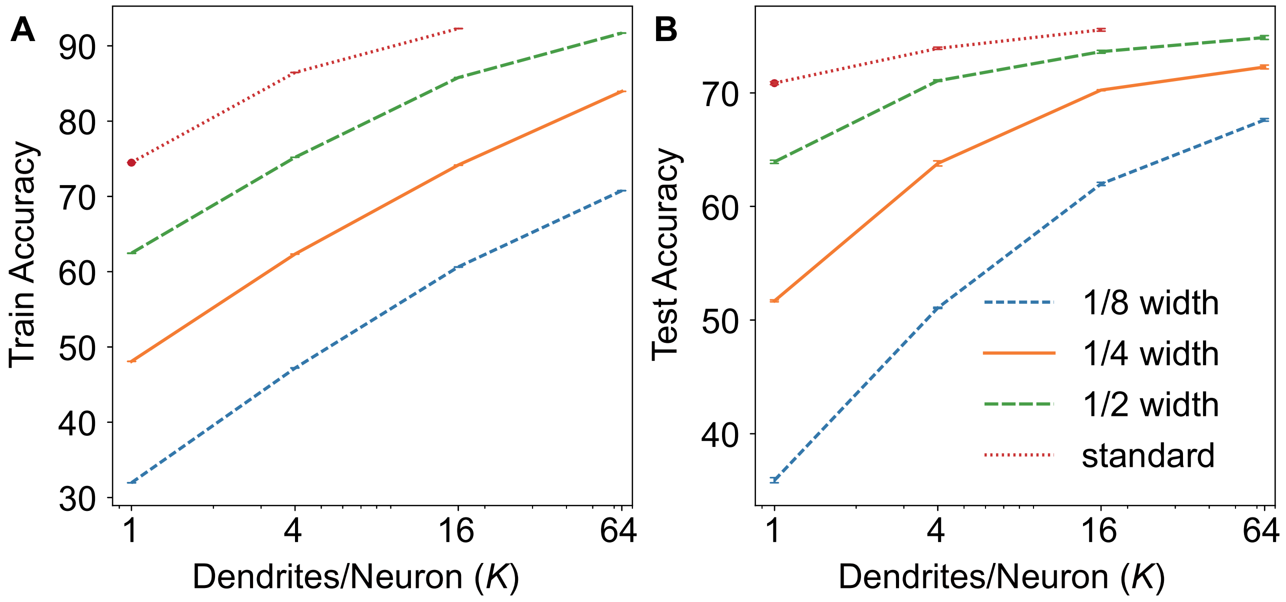

Figure S7: Comparison of Resnet-18-style models composed of point and dendritic neurons trained on the ImageNet dataset.
Each experiment was performed 5 times, with standard deviations displayed. (A) Training accuracy, and (B) Test accuracy for models with varying numbers of dendrites per neuron at four distinct levels of network width. x-axis indicates the number of dendrites per neuron; models with one dendrite per neuron are point neuron-based models.

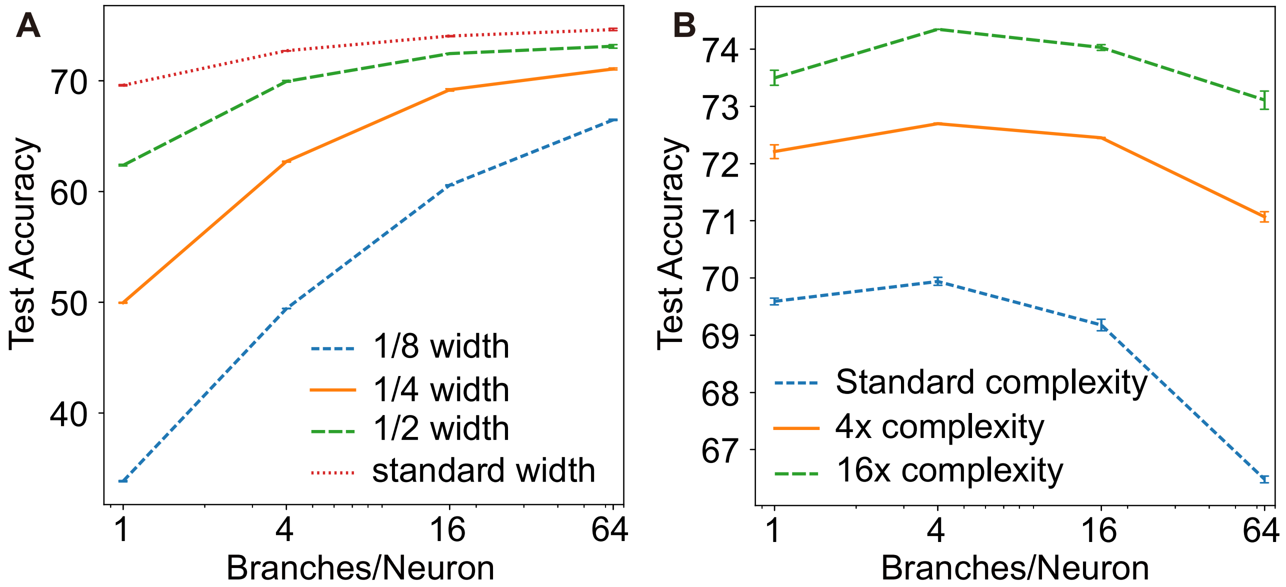

Figure S8: Results on ImageNet dataset using neural network models without residual connections.
Each experiment are performed 3 times, with standard deviations displayed. (A) Test accuracy for models with varying numbers of dendrites per neuron at four distinct levels of network width. (B) Comparison of models with equivalent computational complexities at three different levels. The blue dashed curve represents the baseline and subsequent dendritic models with K values of 4, 16, and 64. The orange curve corresponds to models with twice the number of channels, and the green dashed curve represents four times the number of channels.

model by eliminating the dense residual connections and significantly reducing the number of blocks in the model to arrive at a baseline point neuron based model. Further details regarding the modifications to the models can be found in the accompanying code.

For this part, we carry out two distinct sets of experiments. The first set focuses on models of equivalent computational complexity, and the second emphasizes models sharing the same inter-layer communication cost.

In the first set of experiments, we evaluated models of two distinct computational complexity levels, varying the neuron configurations. Specifically, the configurations encompassed point neurons and dendritic neurons with varying numbers of dendrites. The results for this segment of experiments are displayed in Table S2. Analogous to previous experiments, we observed that models utilizing dendritic neurons were able to achieve comparable performance relative to the point neuron-based models with equivalent computational complexity if they are equipped with efficient inter-layer communication bandwidth.

The second set of experiments is conducted employing models that retain the same inter-layer communication cost. Our experimental procedure begins with a point neuron-based model, which possesses one-fourth of the inter-layer communication complexity compared to the baseline model. This point neuron model is subsequently replaced with dendritic neuron models that contain 4 and 16 dendrites respectively. The corresponding results are systematically presented in Table S3. Upon analyzing these results, it becomes apparent that the performance of the model progressively enhances as we incorporate neurons with an increased number of dendrites.

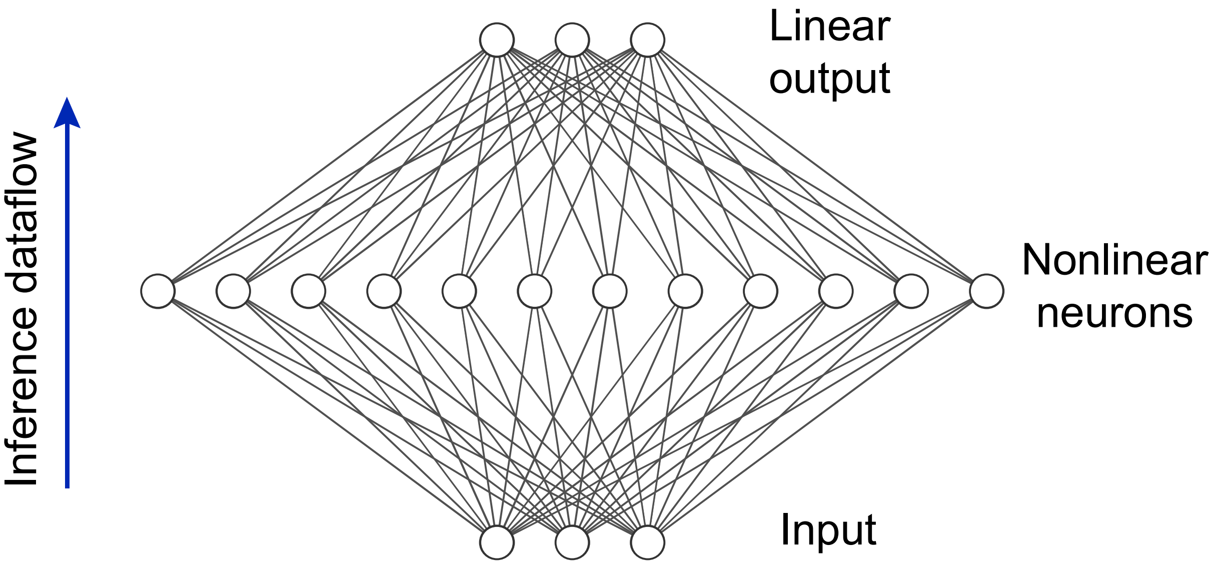

Figure S9: Schematic representation of a bottleneck neural network module comprising two interconnected layers.

Table S2: Comparison of the performance of dendritic models with varying numbers of dendrites per neuron on the LibriSpeech dataset.
The table presents models with two levels of computational complexity. To maintain equivalent computational complexity when increasing the number of dendrites in a neuron, the number of inter-layer channels is proportionally reduced, as indicated in the table.

**# of Dendrites**	**Channel scaling factor**	**Test error**
1 st complexity level(baseline)		
1 (baseline)	1	7.72
4	1/2	7.92
16	1/4	8.28
2nd Complexity level		
1	2	6.69
4	1	6.69
16	1/2	6.89

Table S3: Performance Evaluation of Dendritic Models with varying dendritic counts per neuron evaluated on the LibriSpeech Dataset. The models in this comparison have the same inter-layer communication cost.

# of Dendrites	Channel scaling factor	Test error
1	1/4	15.39
4	1/4	10.49
16	1/4	8.23

References

S1. Cover, T. M., and Thomas, J. A. Elements of Information Theory (Wiley Series in Telecommunications and Signal Processing). Wiley-Interscience (2006).

S2. Murty, U., and Bondy, A. Graph Theory (graduate texts in mathematics 244). Springer (2008).

S3. Kerr, A., Merrill, D., Demouth, J., and Tran, J. Cutlass: Fast linear algebra in cuda c++ (2017). https://developer.nvidia.com/blog/cutlass-linear-algebra-cuda/.

S4. Choquette, J., Gandhi, W., Giroux, O., Stam, N., and Krashinsky, R. (2021). Nvidia a100 tensor core gpu: Performance and innovation. IEEE Micro *41*, 29–35.

S5. Tillet, P. Matrix multiplication; triton documentation (2020). https://triton-lang.org/main/index.html.

S6. Smith, T. M. Theory and practice of classical matrix-matrix multiplication for hierarchical memory architectures. Ph.D. thesis The University of Texas at Austin (2018).

S7. Olivry, A. Automatic derivation of i/o complexity bounds for affine programs. Ph.D. thesis Université Grenoble Alpes (2022).

S8. Krizhevsky, A., Hinton, G. et al. Learning multiple layers of features from tiny images. Tech. Rep. Toronto, ON, Canada (2009).

S9. He, K., Zhang, X., Ren, S., and Sun, J. (2016). Deep residual learning for image recognition. In: Proceedings of the IEEE conference on computer vision and pattern recognition (CVPR). IEEE (770–778).

S10. Hassani, A., Walton, S., Shah, N., Abuduweili, A., Li, J., and Shi, H. (2021). Escaping the big data paradigm with compact transformers. Preprint at arXiv. https://doi.org/10.48550/arXiv.2104.05704.

S11. Panayotov, V., Chen, G., Povey, D., and Khudanpur, S. (2015). Librispeech: an asr corpus based on public domain audio books. In: 2015 IEEE international conference on acoustics, speech and signal processing (ICASSP). IEEE (5206–5210).

S12. Li, J., Lavrukhin, V., Ginsburg, B., Leary, R., Kuchaiev, O., Cohen, J. M., Nguyen, H., and Gadde, R. T. (2019). Jasper: An end-to-end convolutional neural acoustic model. Preprint at arXiv. https://doi.org/10.48550/arXiv.1904.03288.
