# Supplementary material for: Dendritic nonlinearities mitigate communication costs
Source: Patterns (N Y). 2026 Mar 30;7(6):101520. doi: 10.1016/j.patter.2026.101520 (PMC13280677; doi:10.1016/j.patter.2026.101520)
Supplement: Document S2. Article plus supplemental information [file mmc2.pdf]

Article

Patterns

Dendritic nonlinearities mitigate communication costs

Highlights

- Dendritic nonlinearities reduce communication costs in neural networks
- Active dendrites match point-neuron capacity when controlling for complexity
- Localized feature aggregation significantly lowers memory access overhead
- Dendritic structures enable efficient network scaling

Authors

Xundong Wu, Pengfei Zhao, Zilin Yu, ..., Gang Pan, Panayiota Poirazi, Tiejun Huang

Correspondence

wuxundong@gmail.com (X.W.), poirazi@imbb.forth.gr (P.P.)

In brief

Artificial neural networks typically ignore the complex, active branches found in biological neurons. This study challenges the assumption that these “dendritic nonlinearities” primarily boost computational power. Instead, the authors show they function like efficient data filters. By processing signals locally before transmission, these nonlinear structures drastically reduce the “traffic” required between neurons. This reveals a biological blueprint for scaling AI by solving communication bottlenecks rather than just increasing calculation capacity.

Wu et al., 2026, Patterns 7, 101520
June 12, 2026 © 2026 The Author(s). Published by Elsevier Inc.
https://doi.org/10.1016/j.patter.2026.101520

CellPress

Patterns

CellPress
OPEN ACCESS

Article

Dendritic nonlinearities mitigate communication costs

Xundong Wu,[1,2,6,8,9,*] Pengfei Zhao,[2,8] Zilin Yu,[3,8] Lei Ma,[2,5,8] Yifan Gao,[1] Ka-Wa Yip,[1] Huajin Tang,[6] Gang Pan,[6] Panayiota Poirazi,[7,*] and Tiejun Huang[2,4]
[1]Zhejiang Lab, Hangzhou, Zhejiang, China
[2]Beijing Academy of Artificial Intelligence, Beijing, China
[3]Bytedance, Beijing, China
[4]National Key Laboratory for Multimedia Information Processing, School of Computer Science, Peking University, Beijing, China
[5]National Biomedical Imaging Center, Peking University, Beijing, China
[6]Zhejiang University, Hangzhou, China
[7]IMBB-FORTH, Heraklion, Crete, Greece
[8]These authors contributed equally
[9]Lead contact
*Correspondence: wuxundong@gmail.com (X.W.), poirazi@imbb.forth.gr (P.P.)
https://doi.org/10.1016/j.patter.2026.101520

THE BIGGER PICTURE Modern AI relies heavily on simplified “point neurons.” While there is active research on complex, bio-inspired dendritic units, their adoption remains limited. We argue that active dendritic units offer learning capacity comparable to point neurons, their real evolutionary and engineering advantage lies in minimizing communication costs. By mimicking the brain’s strategy of using active dendrites to nonlinearly aggregate features locally, we can design neural architectures that scale efficiently without a prohibitive increase in data traffic. Research in this direction could have important implications for the development of future AI hardware and software. By shifting architectural priorities from dense signal transmission to optimizing local feature aggregation, neural network hardware can be substantially more efficient. Achieving sustainable, large-scale AI requires us to re-imagine the neuron not just as a calculator but as a communication optimizer. By organizing these units in a way that respects the physical constraints of data movement—similar to how brains are optimized for wiring efficiency—we can overcome the connectivity bottlenecks that currently limit the scaling of artificial intelligence.

SUMMARY

Why have modern artificial neural networks not adopted the nonlinear dendritic structures found in biological brain cells, and what is the core advantage of such active dendritic units? While early studies suggested that dendritic nonlinearities can enhance learning capabilities by boosting capacity, we provide empirical evidence reassessing this. Using extensive machine learning experiments, we show that dendritic nonlinearities in neural networks offer comparable learning capacity to standard point-neuron models when controlled for parametric complexity. Instead, we believe that their key advantage lies in enabling network scaling while substantially reducing communication costs via localized feature aggregation. Our experiments and analysis suggest that incorporating nonlinear dendritic architectures can significantly lower memory access or data transfer overhead during neural network inference—the primary sources of energy consumption in modern AI systems—and potentially during training as well. We argue that these insights motivate further theoretical and architectural exploration of dendritic-like structures in artificial neural networks.

INTRODUCTION

Over the past decade, artificial neural networks (ANNs) have significantly advanced across diverse domains, demonstrating impressive performance on complex tasks.[1,2] Despite their inspiration from biological neuronal networks, modern ANNs use highly simplified “point neurons,” described mathematically as

$$h = \sigma\left(\sum_{i=1}^{n} w_i x_i + b\right), \qquad \text{(Equation 1)}$$

© 2026 The Author(s). Published by Elsevier Inc.
This is an open access article under the CC BY license (http://creativecommons.org/licenses/by/4.0/).

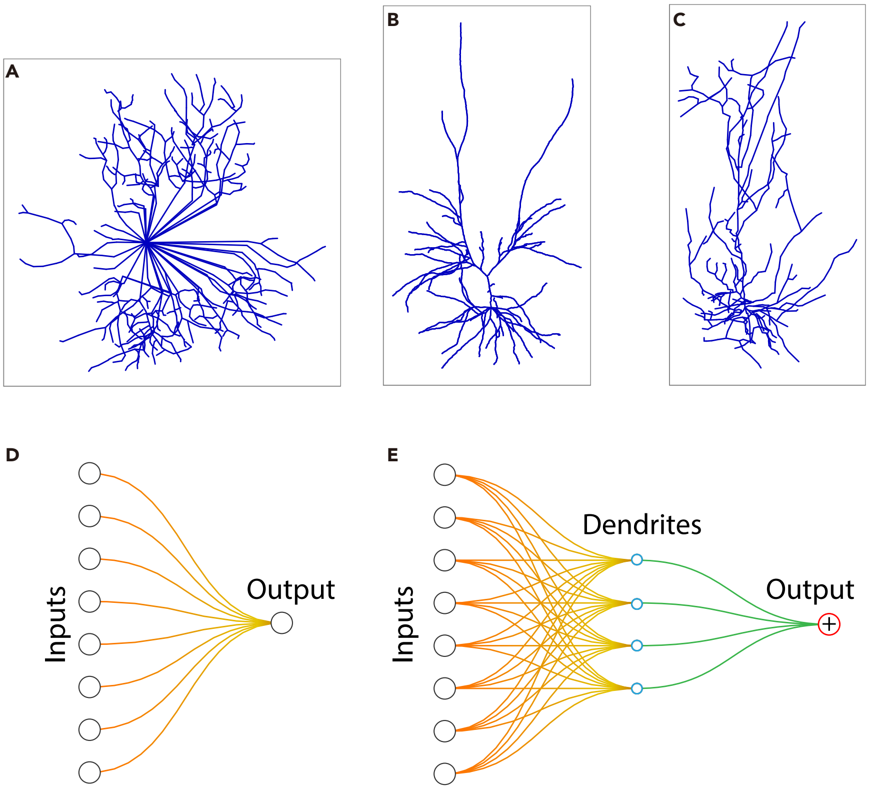

Figure 1. Representative biological neurons illustrating dendritic structures contrasted with mathematical point and dendritic neuron models

(A–C) Illustration of three representative neurons showcasing distinct dendritic structures from left to right: a chicken bipolar neuron,[3] a human hippocampal pyramidal neuron,[4] and a ferret neocortical pyramidal neuron.[5] All neuronal morphologies are from the Neuromorpho.org database.[6]

(D) Portrays a point neuron, as characterized by Equation 1.

(E) Illustrates a dendritic neuron with 4 dendritic branches as detailed by Equations 2 and 3.

where inputs (x_i), weights (w_i), bias (b), and nonlinear activation (σ) produce neuron output (h). These neurons differ drastically from biological neurons, which have elaborate dendritic structures (Figure 1).

Dendritic structures in biological neurons offer enhanced surface-area-to-volume ratios, essential for forming numerous synaptic connections within limited brain space.[7–9] Unlike biological brains, ANNs executed on general-purpose hardware (CPUs and GPUs) do not have physical constraints, as in biological brains, raising questions about the necessity of dendrites in artificial systems. Here, we argue that dendrites are indeed valuable beyond biological contexts.

Dendrites are known to facilitate local nonlinear operations due to their anatomical compartmentalization and specialized voltage-gated ion channels.[7,10–12] These localized nonlinearities may enable key computational functions such as coincidence detection, signal amplification, learning, context association, enhanced feature extraction, and temporal discrimination.[13–17] Most importantly, it has long been believed that dendritic nonlinearities can endow neurons with greater model capacity than point-neuron-based models,[13,18] among many others.

However, our findings challenge this assumption. Approaching the question from a machine learning perspective, we demonstrate that adopting neurons with active dendrites has little effect on model learning capacity. Instead, we show that adopting active dendrites can significantly reduce communication costs in neural network models. Specifically, dendritic architectures enable localized processing that reduces the number of transmitted features without degrading performance. This is particularly important because communication overhead—primarily from data movement—dominates energy consumption in ANNs,[19] echoing biological evidence that highlights the high cost of axonal transmission relative to local computation.[20]

Our findings indicate that the dendritic-neuron-based model's expanded learning capacity[13,18] likely arises primarily from the sparse structure employed in their models and redundancy avoidance attributed to the smaller unit size of dendrites. Thus, we posit that the primary advantage of active dendrites lies in mitigating communication costs through effective local feature aggregation.

A recent publication[21] also employs nonlinear dendritic components and demonstrates marked improvements in parameter efficiency. While their conclusions may seem to differ from ours at first sight, the two studies address complementary aspects of dendritic computation. The analysis in Chavlis and Poirazi[21] emphasizes architectural and sampling-related benefits, without specifically separating the effects of weight sparsity. The present work aims to clarify the distinct sources of dendritic advantages reported in those earlier studies by systematically isolating the role of dendritic nonlinearities. Our findings suggest that these nonlinearities offer intrinsic benefits in reducing communication costs—an aspect particularly relevant for both modern neural network hardware and the evolutionary optimization of biological neural systems.

Our investigation also finds that adopting a dendritic structure can potentially significantly reduce memory access and occupancy during inference and training of ANN models. These results have important implications for the development of efficient ANN architectures and hardware for real-world applications.

In summary, this work makes the following contributions.

- We demonstrate that dendritic nonlinearities provide comparable learning capacity to point neurons when normalized for computational complexity, challenging the assumption that their primary benefit is enhanced expressivity.

CellPress
OPEN ACCESS

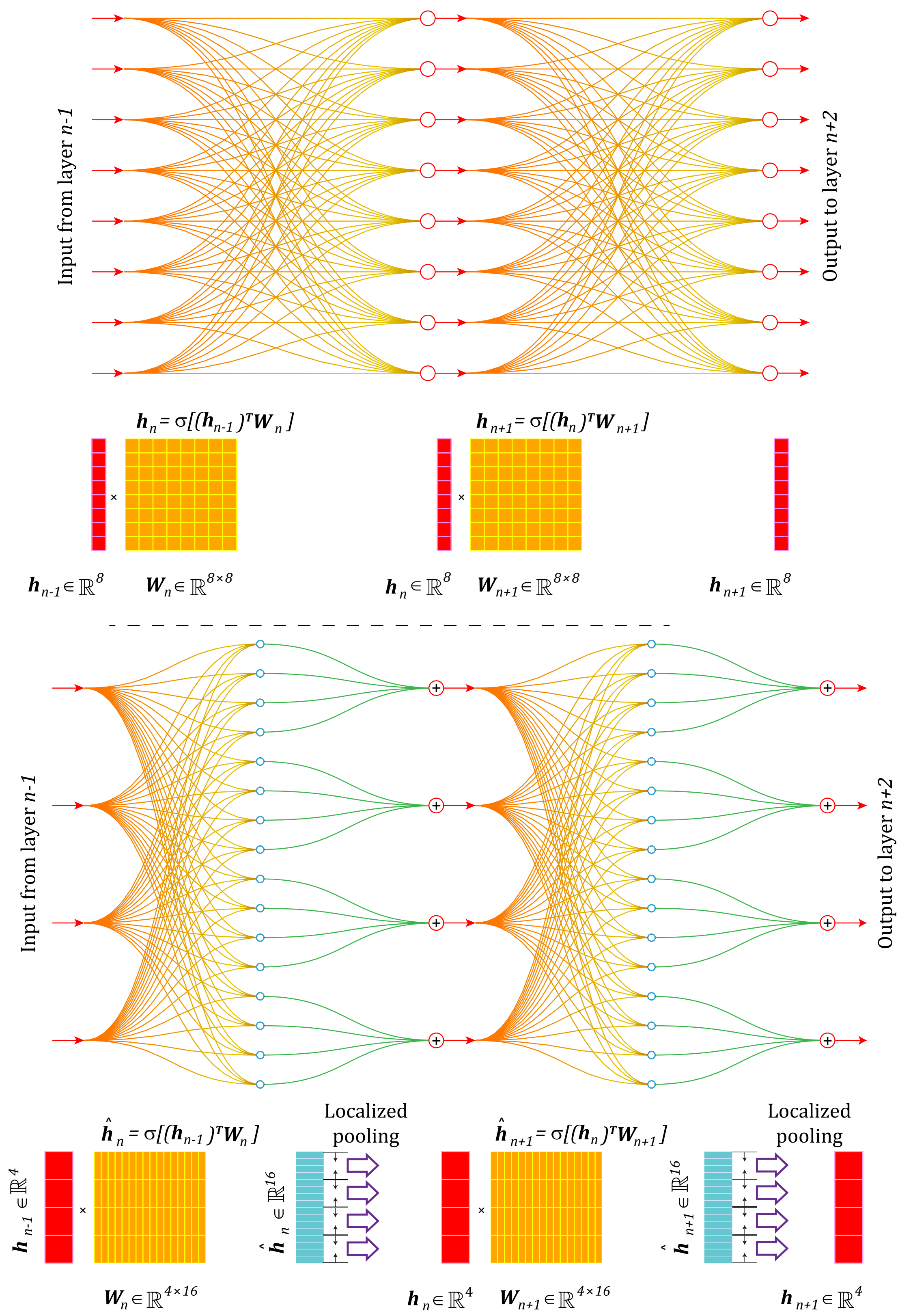

(legend on next page)

- We identify reduced communication cost as the primary advantage of active dendrites, enabling efficient local feature aggregation that scales effectively with network size.
- We provide a theoretical framework and empirical evidence showing that dendritic architectures can significantly lower memory access overhead during ANN inference and training.

RESULTS

Mathematical formulation of the dendritic neuron

In this study, we use a simplified dendritic neuron model,[10,22] which is depicted in Figure 1E and mathematically described by Equations 2 and 3. In this architecture, incoming signals at each dendrite are integrated by computing the dendritic output d_j, which is obtained from the weight vector $\boldsymbol{w}_j$, input activation $\boldsymbol{x}$, and an optional bias b_j. The dendritic output is transformed by an element-wise nonlinear function σ and subsequently summed to produce the somatic output h, conveyed to downstream recipients:

$$\widehat{d}_j = \boldsymbol{w}_j^\top \boldsymbol{x} + b_j, d_j = \sigma\left(\widehat{d}_j\right) \quad \text{(Equation 2)}$$

$$h = \sum_{j=1}^{K} d_j . \quad \text{(Equation 3)}$$

Each dendritic unit described here has the same information-processing capacity as a point neuron but differs in how its output is conveyed downstream. Unlike point neurons, whose outputs are independently transmitted to downstream neurons, dendrites share a common channel for output transmission, typically resulting in information loss. This property is detailed in Note S1.

Communication versus computing in neural networks

In neural networks, the energy required for computation is substantial, but communication is the primary energy bottleneck in modern hardware.[19] Communication can incur orders-of-magnitude higher costs than computation; for instance, as reviewed by Dally et al.,[19] adding two 32-bit numbers may consume approximately 20 femtojoules (fJ), while fetching these numbers from memory can require about 1.3 nanojoules (nJ)—roughly 64,000 times more energy.

Biological brains also exhibit significant energy expenditure and structural investment related to communication, as indicated by extensive white matter volume[23] and high metabolic demands.[20,24,25] The evolutionary pressure on biological systems to minimize these costs suggests potential insights for artificial systems. Our study proposes that incorporating active dendrites into ANNs can significantly mitigate communication-related energy expenses.

Evaluating the communication efficiency of the dendritic structure

Developing the dendritic neuron model

To investigate the role active dendrites can play in neural networks, we compare the performance of models that are constituted with point neurons or dendritic neurons, respectively. We substitute the point neurons in conventional neural network models with dendritic neurons. Each nonlinear summation unit—point neuron or active dendrite—receives at most one copy of a specific input from the previous network layer. Each dendrite within a neuron receives an equal number of dense connections; hence, a dendritic neuron with K branches receives K times more inputs than its point-neuron counterpart. Clearly, those two models are of very different computing and parametric complexity. We need to compare models on an equal footing.

Figure 2 visually compares how layers of point neurons (top) and dendritic neurons (bottom) are structured in a neural network. Consider, for instance, a point-neuron-based fully connected layer with $D_{point} = 8$ inputs and $D_{point} = 8$ output neurons (top); its parametric/computing complexity is $8^2 = 64$. Now, consider a dendritic neuron layer (bottom) with $D_{dendritic}$ input/output neurons and K dendrites per neuron—its parametric/computing complexity is $KD^2_{dendritic}$. To make these layers comparable in complexity, we need to set $D_{dendritic} = D_{point}/\sqrt{K}$. So for our example with $D_{point} = 8$ and $K = 4$, we set $D_{dendritic} = 4$. Throughout this study, because parametric and computational complexities always scale identically, we will primarily refer to parametric complexity for simplicity.

To compare communication costs, let D be the total number of neurons in a layer or network and define $\Psi = D_{dendritic}/D_{point}$ as the ratio of D relative to a point-neuron baseline. In the example, the point-neuron layer has $D_{point} = 8$, while the dendritic layer has $D_{dendritic} = 4$, resulting in $\Psi = 0.5$.

Dense models on ImageNet

We begin by employing the ResNet-18 network[26] as a baseline point-neuron-based model, a widely utilized computer vision model. For this set of experiments, we modify the ResNet-18 network architecture as described above to replace typical point neurons with dendritic neurons. Since the dimensionality of our network's input and output remains fixed, the input and output layers are addressed differently, as detailed in the model architectures section.

The outcomes of this experiment are shown in Figure 3. We compare models with three levels of complexity. The light-blue dashed curves represent experimental results obtained from various models with a complexity level equal to that of the standard ResNet-18 model. The leftmost data point (①) corresponds to the standard ResNet-18 model, which serves as a baseline. Subsequent data points to the right denote dendritic models with K values of 4, 16, and 64, respectively. Concomitantly, these models' D values have been adjusted to 1/2, 1/4, and 1/8 of the original model's values, respectively. Given that the models we study here are convolutional neural networks, we change D by

Figure 2. Structural comparison of neural network layers utilizing point neurons versus dendritic neurons
Comparison of neural network layers using point neurons (top) and dendritic neurons (bottom). Only two layers from each model are depicted. The point-neuron model has $D = 8$ channels, whereas the dendritic neuron model features neurons with $K = 4$ dendritic branches each, leading to an effective $\widehat{D} = \frac{D}{\sqrt{4}} = 4$ channels. This ensures that both models have comparable parametric and computational complexities. Note: tensor dimensions are symbolized by a mesh of patches; however, patch sizes do not reflect actual scale. Bias terms have been excluded for simplicity.

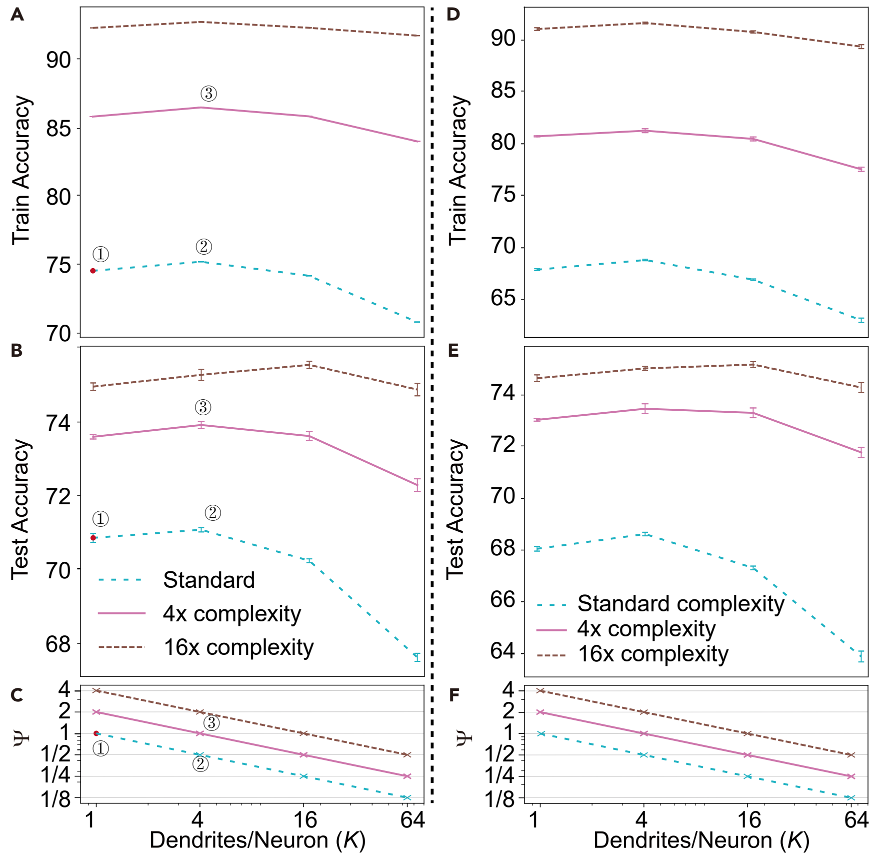

Figure 3. Performance analysis of dense and sparse ResNet-18 models on ImageNet using point versus dendritic neurons

(A–C) Experiment on dense models (5 trials; SD shown). The red dot (①) marks the baseline ResNet-18 with standard point neurons ($K = 1$). The x axis indicates the number of dendrites per neuron (K). Three complexity levels are evaluated: standard complexity (light-blue dashed curves), models with the same complexity as baseline ResNet-18. 4× complexity (solid magenta curves), and 16× complexity (brown dashed curves). Within each curve, models share the same total parametric budget but differ in K (number of dendrites per neuron). For example, model ② is configured with a Ψ ratio of 0.5 to match the complexity of the baseline. Model ③, with $\Psi = 1$ and $K = 4$, has 4× the complexity of the baseline model ①.

(A) Training set accuracy.

(B) Test set accuracy.

(C) Ψ ratio relative to the baseline ResNet-18.

(D–F) Same layout and analysis as in (A)–(C) but for sparse models (3 trials; SD shown).

scaling up/down the number of channels in network layers. By maintaining this configuration, four models on the same curve have Ψ values of 1, 0.5, 0.25, and 0.125 from left to right, as shown in Figure 3C, all while preserving equivalent parametric and computational complexities (see Note S2 for a detailed complexity comparison between models).

The solid magenta curves represent data from models where D is doubled compared to the experiments shown by the light-blue curve. Similarly, the brown dashed curves illustrate models where D values are quadrupled. For the brown dashed curve, the point-neuron-based model at the left end of the curve has 4 times the number of neurons ($\Psi = 4$ in Figure 3C) for each layer compared to the standard ResNet-18 model (①). At the right end of the brown curve, we can see the dendritic model, which is equipped with $K = 64$ dendrites per neuron, thus having just one-eighth of neurons ($\Psi = 0.125$).

Our analysis yields a particularly intriguing result concerning the communication cost of dendritic neuron models compared to point-neuron-based models. Specifically, we find that for models of equivalent computing complexity, a dendritic neuron model achieves comparable performance to a point-neuron-based model when Ψ is greater than or equal to 0.25.

This reduction in D offered by adding dendrites can significantly reduce the communication cost between neurons in ANNs.

To obtain a more complete picture, we also compare models with the same number of neurons. The results are illustrated in Figure S7.

Sparse models on ImageNet

Thus far, we developed dendritic-neuron-based models with significantly reduced communication costs, as measured by D. These dendritic-neuron-based models can also achieve similar (or slightly better) performance compared to corresponding point-neuron-based models of the same computing complexity, in terms of both model expressivity and generalization performance.

This may seem contradictory to earlier works,[13,18] where dendritic-neuron-based models showed higher capacity/expressivity than point-neuron-based models of the same parametric complexity. One might argue that the models we evaluated thus far are non-sparse, which is not biological and differs from the models evaluated in earlier studies. As such, it is essential to also investigate the influence of sparsity on model behavior.

As illustrated on the right side of Figure 3, we study sparse models with 85% of parameters pruned. Although sparsity generally reduces performance, we observe the same trend as in the non-sparse case: models with different dendritic numbers K but equal computing complexity show little performance difference between dendritic and point-neuron architectures as long as Ψ is above 0.25. This reinforces our earlier findings and highlights communication efficiency as the key advantage of dendritic neuron models.

Additional empirical verification

To further substantiate our findings, we conducted additional experiments using a diverse array of model architectures and datasets. This analysis included an assortment of models, including those lacking residual connections and those that leverage transformer-based architectures and one-dimensional (1D) convolutional networks for speech recognition. For the sake of clarity, we have included these additional results in Note S5.

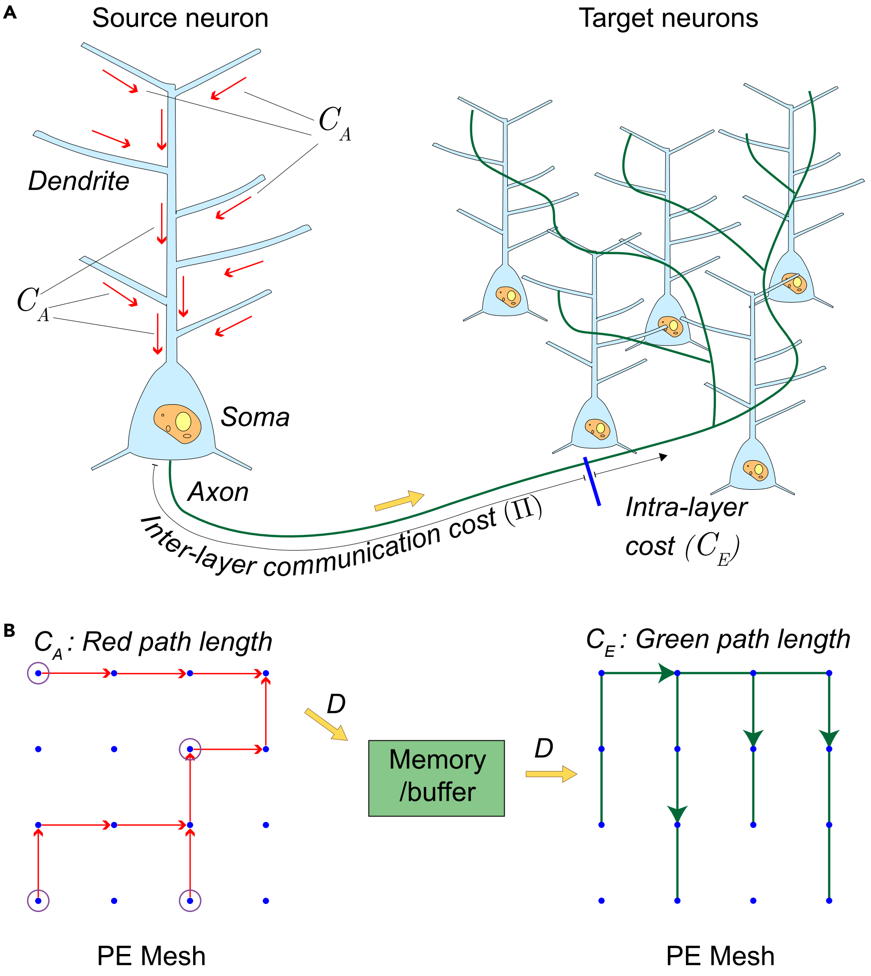

Figure 4. Illustration of communication cost metrics (C_A, D, and C_E) in biological and artificial neural networks

(A) Biological neural networks.

(B) ANNs.

In this context, C_A represents the communication cost associated with aggregating synaptic inputs, D denotes the inter-layer communication cost, and C_E signifies the expense related to signal propagation to each synapse (weight). We measure C_A and C_E by the total path length over which the signal traverses. For the ANN models, we assume model inference is performed on a mesh of processing elements (PEs). Each blue dot represents one PE unit in the mesh. It is important to note that the division into these three metrics is not intended to be exact.

Similar conclusions are drawn from these supplementary experiments.

Local communication cost analysis

Our analysis demonstrates that incorporating dendritic structures into neural networks significantly reduces the required neuron count (D) without sacrificing performance, provided the D is sufficiently large. In biological brains, fewer neurons correspond to reduced volume and connectivity, potentially decreasing both neuronal soma volume and the white matter, which consists predominantly of long-range axons and constitutes approximately 60% of the brain's total volume.[27] For ANNs running on hardware such as GPUs, reducing D cuts costs by limiting data transfers to off-chip memory and decreasing memory usage for hidden-layer activations during inference. We define this neuron-count-related cost D as the inter-layer communication cost.

Although dendritic architectures lower D, Figure 2 shows that dendritic neurons require more synaptic connections per input neuron to match point-neuron model complexity/performance. Thus, communication costs must also account for connecting these additional synapses (weights).

Figure 4 provides a breakdown of communication costs in both biological networks and ANNs, dividing costs into three parts: intra-neuron aggregation cost (C_A), inter-layer communication cost (D), and intra-layer signal propagation cost (C_E). Costs C_A and C_E are measured by the signal's travel distance, while D reflects neuron count.

The top image of Figure 4 depicts the communication costs in biological neural networks. Here, C_A corresponds to the cost of aggregating outputs from the dendritic tree and sending them to the cell body. Due to the challenge of separating C_A from computation costs, this is not intended to be accurately defined. D represents the long-range communication cost, while C_E denotes the cost of axonal activation reaching the target synapses.

The bottom image in Figure 4 illustrates the same metrics for ANNs, assuming inference is performed on a mesh of processing elements (PEs). The left section shows aggregation costs (C_A), the middle represents inter-layer costs (D, including memory storage considerations), and the right indicates intra-layer costs (C_E), measured by path length between PEs.

Returning to Figure 2, we emphasize the importance of considering communication costs beyond inter-layer interactions. The point-neuron model receives and outputs data of dimension D. In contrast, the dendritic neuron model preserves complexity by using fewer neurons ($\hat{D} = D/\sqrt{K}$) but distributing inputs across more dendrites ($D\sqrt{K}$). In this case, D and $\hat{D}$ correspond to the number of neurons for the network layer. Although both model architectures process the same total number of inputs (D^2), differences in neuron and dendrite configurations significantly influence communication costs—captured by C_A, C_E, and D—as detailed in the following analysis.

Cost estimation for a biological neuronal network

We quantified the wiring (communication) costs, C_E, required to connect $\hat{D} = D/\sqrt{K}$ input neurons to $D \cdot \sqrt{K}$ synapses each, resulting in a total of D^2 synapses in biological neuronal networks. These costs were evaluated across various dendritic counts per neuron, $K = \{1,4,16,64\}$, assuming synapses are spatially

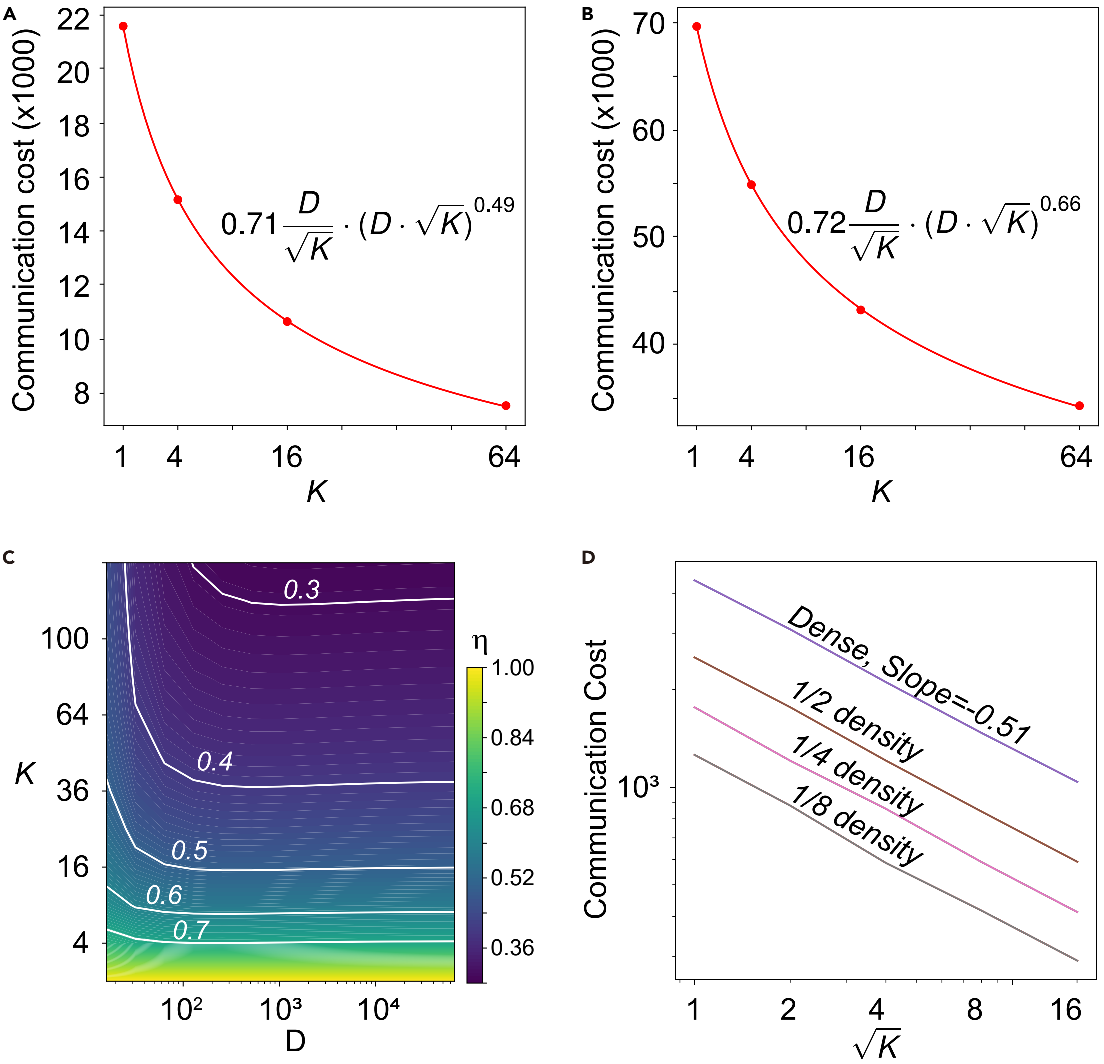

Figure 5. Estimation of signal propagation costs in biological and artificial neural network layers
(A and B) Estimation of signal propagation costs C_E for a biological network layer with a varying number of dendrites per neuron (K) and a baseline network of dimension $D = 1{,}024$. Post-synaptic targets were sampled from (A) a unit square and (B) a unit cube. In each image, the curve and its corresponding equation are fitted to the data points. In the biological model (A/B), C_E is estimated via Euclidean minimal spanning trees (green path in A), while C_A (red path) is not estimated.
(C and D) Estimation of signal propagation costs for an ANN layer. (C) Topographic representation of the ratio $\eta = (\widehat{C}_A + \widehat{C}_E)/(C_A + C_E)$: the visualization highlights the influence of the variations in D and K on the η. (D) demonstrates the variations in $\widehat{C}_E$ as a function of $\sqrt{K}$ and levels of connection sparsity. The axes are depicted on a logarithmic scale. When $K = 1$, the models are based on point neurons. For this experiment, a D value of 256 was utilized. The slope is obtained from fitting a line to the logarithm of C_E against the logarithm of $\sqrt{K}$.

distributed either in a two-dimensional (2D) plane or a three-dimensional (3D) volume. Both empirical measurements and fitting with theoretical predictions are presented in Figures 5A (2D case) and 5B (3D case). The special case $K = 1$ corresponds to the point-neuron model. Clearly, the results demonstrate a significant advantage in adopting dendritic neurons. Further methodological details are provided in the communication cost analysis portion of the methods section.

We do not attempt to estimate the impact of having dendritic neurons on C_A for biological neuronal networks due to the scarcity of biological data and difficulty in separating the computing cost from the aggregating communication cost. However, we speculate that the cost of signal aggregation in biological neurons will be mostly dependent on the number of inputs, and thus adopting a nonlinear or linear dendrite would not significantly affect this type of cost.

Cost estimation for an ANN

We analyzed the communication cost of ANNs using a simplified parallel architecture model (see Note S3 for the detailed mathematical derivation, including the PE grid definition and minimal rectilinear spanning tree [MRST] calculations). Our results show that incorporating dendritic neuron structures in ANN models can significantly reduce on-chip communication cost compared to traditional point-neuron models. Specifically, for

fixed computational complexity, dendritic models consistently exhibit lower communication costs as the number of dendrites per neuron K increases.

Figure 5C shows that the ratio of communication costs η between dendritic-neuron- and point-neuron-based models decreases with increasing K. Moreover, as shown in Note S3, in most configurations, the communication cost for dendritic models is dominated by $\widehat{C}_E$, especially for large input dimensions D, which is common in practice.

Further analysis reveals that $\widehat{C}_E$ decreases with increasing model sparsity and increasing K, following a negative power-law relationship with $\sqrt{K}$, specifically $\widehat{C}_E \propto K^{-0.51}$, as shown in Figure 5D. This closely matches the simplified theoretical form of $\widehat{C}_E$ derived in Equation S9, which includes a $K^{1/4}$ scaling factor. These findings suggest that dendritic neurons can effectively reduce communication costs in sparse ANNs.

Reducing memory access cost during training and inference on a GPU

Model inference

We also extended our analysis to modern GPU-based architectures. Theoretical estimates indicate that dendritic neurons can reduce global memory access and improve efficiency by a factor of $\sqrt{K}$, highlighting their potential for improving performance in realistic parallel hardware settings. We also verify the theoretical result with empirical experiments. Further details are provided in Note S4.

Model training

We have demonstrated that dendritic architectures can reduce communication costs during model inference. Can they similarly decrease memory communication costs during training?

At first glance, dendritic models require storing more intermediate activations during training compared to standard models. For example, in Figure 2, a dendritic-neuron-based layer stores 16 + 4 = 20 intermediate activations, while a point-neuron-based layer only stores 8 activations. Counting each activation value once (as obtaining post-activation function values from pre-activation function values incurs minimal cost), this translates to 320 (dendritic neuron) versus 128 (point neuron) bits per layer using 16-bit floats.

However, memory costs can be significantly reduced by leveraging gradient properties of commonly used activation functions (rectified linear unit [ReLU], leaky ReLU, GELU [Gaussian error linear unit], etc.). Naively, backpropagation through dendritic neurons requires storing intermediate activations h and dendritic pre-activations $\widehat{d}_j$ (see Equations 2 and 3). Examining the gradient computations for a dendritic network layer with input $\mathbf{x}$, dendritic pre-activation $\widehat{d}$, and neuron-layer output h,

$$\frac{\partial h}{\partial \mathbf{w}_j} = \sigma'\left(\widehat{d}_j\right) \cdot \mathbf{x}, \frac{\partial h}{\partial \mathbf{x}} = \sum_{j=1}^{K} \sigma'\left(\widehat{d}_j\right) \cdot \mathbf{w}_j. \quad \text{(Equation 4)}$$

We observe that the gradient calculation here does not depend on $\widehat{d}$ or d but on $\sigma'\left(\widehat{d}_j\right)$. For ReLU, this derivative is binary (0 or 1), allowing representation with a single bit per dendrite. Since only h (not $\widehat{d}_j$) is needed for gradient computations of the subsequent layer (in the forward direction), it suffices to store just one bit per dendrite. In the example shown in Figure 2, this reduces memory from 320 bits (16-bit floats) to only 16 + 4 $\times$ 16 = 80 bits, even less than the point-neuron-based model (128 bits). Similar reductions are achievable for related piecewise activation functions (e.g., Leaky ReLU, parametric ReLU, and ReLU6).

Non-piecewise activation functions (e.g., GELU, ELU [exponential linear unit], and SELU [scaled exponential linear unit]) may require slightly higher precision. However, their gradient values typically span limited ranges, possibly enabling efficient storage using only a few bits per dendrite (e.g., two bits). Given the robustness of neural network training to gradient noise,[28] this approximation may be acceptable in practice. A detailed investigation is left for future work.

DISCUSSION

This work was inspired by biological neurons, which aggregate signals from dendrites into a single output at the soma. Dendrites integrate inputs nonlinearly through voltage-gated channels and receptors, so we designed neural network units that mimic these characteristics.

Treating dendrites as individual point neurons (as previously proposed[10]), the idea of aggregating neuronal outputs is common in deep learning, such as spatial pooling in convolutional networks[29] and Maxout networks,[30] to enhance performance and reduce computational complexity. Prior studies employing dendrite-inspired pooling strategies reported superior computational and discriminatory capabilities compared to linear integration methods.[13,18,21]

Also related is the mixture of expert architecture, which is widely used in current mainstream large language models,[31] where information from multiple experts is combined. However, in such systems, the experts are typically not located in close proximity—sometimes they are even distributed across different machines. Unlike the inherently local nature of dendritic architectures, this setup requires costly long-distance communication for information integration. Could a dendritic architecture serve as a more efficient alternative? Further exploration is warranted.

Several related studies include Naud's sparse neuron ensembles,[32] Sezener's dendritic gated network (DGN),[33] and Iyer et al.'s incorporation of active dendrites into ANNs.[34] Naud showed neuron ensembles efficiently communicate combined signals from multiple sources, albeit through a different mechanism. Sezener emphasized performance without exploring how dendrite count affects efficacy. Unlike Sezener, our study evaluates dendritic efficiency, communication costs, and complexity. Iyer et al. focused on shallow ANNs and dendrites' role in continual learning, contrasting with our emphasis on dendritic efficiency in deeper networks.

We demonstrate that dendritic neurons substantially improve communication efficiency as network size scales. Typically, increasing network width scales neuron counts (D) with the square root of parameters, while depth requires linear scaling. Our results demonstrate dendritic architectures can significantly increase parametric complexity without increasing D. This substantially reduces inter-layer communication, lowering data-transfer costs within computing chips, and may have analogous biological benefits,[20] although further exploration of biological wiring costs remains necessary.

Our analysis of dendritic architecture's communication advantages considered only wire length, excluding wiring volume. Earlier research suggests dendrites confer significant volume savings.[9] Accounting for wiring volume would likely enhance our architecture's benefits, but due to limited biological data, this aspect is left for future research.

Also relevant to our study is the concept of small-world networks, which achieve communication efficiency through specific connectivity patterns.[35,36] We instead focus on local dendritic nonlinearities to minimize communication costs.

Our findings carry theoretical and practical implications. Theoretically, dendritic architectures suggest widening networks by enhancing feature complexity rather than solely increasing inter-layer communication. Practically, dendritic models outperform point-neuron models under equal communication budgets, substantially reducing memory access, especially beneficial for large-batch inference. Our results predict that dendritic designs can reduce on-chip communication costs, potentially informing neural accelerator design.

CONCLUSION AND FUTURE WORKS

Finally, our findings parallel evolutionary patterns in biological brains, where complex dendritic structures emerge in larger neural systems due to increased computational demands.[7] Integrating dendritic neurons into artificial networks may thus reflect fundamental biological principles, offering insights for efficient and scalable neural network design.

Several avenues remain for future research. Our analysis of reduced memory costs from dendritic neural network training is limited to ANNs. Whether this benefit translates to biological dendritic neurons remains open, given the limited understanding of biological learning mechanisms, is deferred to future research.

We also lack a complete understanding of why dendritic channel sharing matches or exceeds conventional model performance. One plausible explanation involves interpreting dendritic pooling as low-rank approximations of large weight matrices. Further investigation is necessary.

Notably, our dendritic models apply a single nonlinear layer solely at dendrites. Preliminary results indicated minor performance gains when adding additional somatic nonlinearities, particularly with a large number of dendrites. However, this is not included here, as the primary focus of this study is efficiency rather than incremental performance improvements. Exploring diverse nonlinearities and advanced architectures in dendritic neurons remains an intriguing future work.

This study's analysis of active dendrites relied on machine learning experiments employing a rate-based model. It is crucial to acknowledge that this methodological choice may introduce limitations, particularly when comparing the findings to those derived from spike-based models.

METHODS

Datasets for machine learning experiments

The present study leverages three commonly used datasets, ImageNet, CIFAR-100, and LibriSpeech, for model training and evaluation. These datasets are commonly served as benchmarks in deep learning research.

ImageNet dataset

For this study, we use the ILSVRC 2012 subset of the ImageNet dataset, which comprises 1.2 million training images and 50,000 validation images from 1,000 categories.[37] The images vary in size and are resized to a fixed resolution of 224×224 pixels for uniformity, per the standard ResNet procedure.[26] Typical data augmentation techniques, such as random cropping, random horizontal flipping, and color jittering, were applied during training to enhance the model's generalization ability.

CIFAR-100 dataset

The dataset comprises 60,000 32×32 color images in 100 classes, with 600 images in each class. There are 50,000 training images and 10,000 test images.[38] Like the ImageNet data processing, we followed the typical data augmentation procedure.[26]

LibriSpeech dataset

The dataset is a publicly available English speech corpus for automatic speech recognition (ASR) training and evaluation from the LibriVox project's audiobooks. It consists of 1,000 h of transcribed speech, divided into training, development, and testing subsets.[39] The experiment utilizing this dataset can be found in Note S5.

Model architectures

In this study, we primarily used the ResNet-18 architecture as the baseline model. ResNet-18 is an 18-layer deep residual neural network, a seminal model proposed by He et al.[26] The baseline configuration of ResNet-18 encapsulates an initial convolutional layer, followed by four residual blocks, each of which consists of two convolutional layers. This pattern constitutes the primary structure of our working model; in contrast to the original ResNet-18 model, our adapted architecture positions the shortcut connection after the ReLU activation function. This modification is imperative to ensure the compatibility of the dendritic structure with the model architecture.

For experiments on scaling up networks, we scaled up each network layer by the same designated factor, except for the input and output of the model. For models with dendritic neurons, we replaced neurons in the standard model with dendritic neurons with K dendrites as specified by the experiment setting, except for the input and output layers of the model. To maintain the uniform model complexity scaling throughout the model, we equip the input layer and the penultimate layer of the model with neurons of $\sqrt{K}$ instead of K dendrites. The same setting is also employed in experiments designed to compare models that share identical inter-layer communication costs.

For models trained on CIFAR-100, we observed training instability. Therefore, we clipped the gradient norm to 1.0 during model training. We also added an extra batch norm to each dendrite to improve model stability. This additional batch norm can be fused with the previous layer and thus will not add extra computation burden at the inference stage.

In addition to models based on the ResNet-18 architecture, we have corroborated our findings using a model devoid of shortcut connections. This strategy ensures that the benefits observed are not strictly confined to a particular architecture. The configuration of this model is delineated in Note S5, where the corresponding experimental outcomes can also be found.

Moreover, our experimentation extended to the transformer-based model. Within this model, the standard feedforward layers are substituted with network layers based on dendritic neurons. Comprehensive details pertaining to this modification can be found in Note S5.

Model training

We trained all models with a cosine learning rate decay schedule and the SGD optimizer with a momentum of 0.9.

For ImageNet with dense ResNet models, the learning rate was initialized at 0.4 (instead of 0.1, to compensate for the batch size used for training), and models were trained for 120 epochs, including two warm-up epochs with a learning rate of 0.04. Weight decay was set to 1×10^{-4}. A batch size of 1,024 was employed, and the training was distributed across 8 GPUs.

For ImageNet with sparse ResNet models, the models were trained for 200 epochs with an initial learning rate of 0.1 and 2 warm-up epochs at a learning rate of 0.01. The weight decay parameter was set to 1×10^{-4}. To achieve a sparse ratio of 85%, we applied L1-unstructured global pruning in 5 rounds, conducted between epochs 40 and 140. Subsequently, the models were trained for an additional 60 epochs.

Finally, for CIFAR-100 models, we trained them for 200 epochs with a learning rate of 0.05, including two warm-up epochs at a learning rate of 0.005. A batch size of 64 was utilized, and the weight decay parameter was set to 5×10^{-4}.

Our investigation emphasizes the comparative analysis of the performance of various models under identical training conditions, facilitating an equitable assessment of the distinct capabilities of each model. Consequently, all models within the comparison group undergo training with the same hyperparameters, barring the requisite architecture adjustments. Further details concerning the experiments can be found in the accompanying source code.

Communication cost analysis

Biological neural network

We modeled a baseline network layer with D input and D output neurons, resulting in D^2 synapses. For each input neuron, $D\sqrt{K}$ synaptic targets were randomly distributed within either a unit square (2D) or a unit cube (3D), where K represents the number of dendrites per neuron and is varied across $K = 1, 4, 16, 64$.

To estimate the wiring length (represented as the green path in Figure 4A), we computed the Euclidean minimal spanning tree over each synapse set using the method described by Steele et al.[40] This was repeated 10 times to obtain an average total path length. The communication cost C_E was then calculated as the product of $D/\sqrt{K}$ and the mean path length. We note that we did not attempt to estimate the aggregation cost (C_A, red path) for biological networks due to the complexity of separating computation from aggregation in wetware. Finally, we fitted the results to a function of the form $\alpha \cdot D/\sqrt{K} \cdot (D\sqrt{K})^{\beta}$, allowing us to extract the scaling exponent β and compare it to theoretical expectations.

Artificial neural network

We adopted a simplified parallel explicit communication model (PECM), inspired by Dally et al.,[19] to estimate data movement costs in ANN inference hardware. The model assumes that computation occurs on a 2D grid of processing elements (PEs), interconnected via an on-chip network (NoC) within a unit square. This abstraction captures essential features of neuromorphic and parallel architectures used in real-world ANN hardware.[41–43]

Communication costs were analyzed for both point-neuron- and dendritic-neuron-based models, with derivations provided in the Note S3. Briefly, we utilized a MRST algorithm on a 2D grid to determine the optimal path for data dissemination. The focus was on two key components: aggregation cost C_A and external communication cost C_E, as well as their dendritic counterparts $\widehat{C}_A$ and $\widehat{C}_E$.

We evaluated the communication cost ratio η between dendritic neuron and point-neuron models across a range of parameters: different values of input dimensionality D for point neurons and varying numbers of dendrites per neuron K for dendritic neurons. The impact of sparsity was also assessed by analyzing $\widehat{C}_E$ under varying sparsity levels and dendrite counts.

RESOURCE AVAILABILITY

Lead contact

Requests for further information and resources should be directed to and will be fulfilled by the lead contact, Xundong Wu (wuxundong@gmail.com).

Materials availability

This study did not generate new materials.

Data and code availability

- Our source code is available at GitHub (https://github.com/motifMachine/Dendrite_communication/) and has been archived at Figshare.[44]

ACKNOWLEDGMENTS

This work was supported by the National Natural Science Foundation of China under grant no. 6207608, the NIH under grant no. 1R01MH124867-02, and the Stavros Niarchos Foundation (SNF) jointly with the Hellenic Foundation for Research and Innovation (HFRI) under the 5th Call of "Science and Society" Action "Always Strive for Excellence – Theodoros Papazoglou," grant no. 28056. The authors thank De Ma, Shuicheng Yan, Qianbo Yin, and Mingyang Zhao for their insightful discussions and for sharing their expertise during the development of this work.

AUTHOR CONTRIBUTIONS

Conceptualization, X.W., L.M., T.H., G.P., and H.T.; methodology, X.W., P.Z., Z.Y., and Y.G.; investigation, X.W., P.Z., Z.Y., L.M., K.-W.Y., P.P., T.H., G.P., and H.T.; visualization, X.W., K.-W.Y., and L.M.; writing—original draft, X.W.; writing—review & editing, X.W. and P.P.; resources, X.W., L.M., T.H., G.P., and H.T.

DECLARATION OF INTERESTS

X.W. and Y.G. are inventors on a patent related to this work issued to Zhejiang Lab. X.W., L.M., and T.H. are inventors on a patent related to this work issued to the Beijing Academy of Artificial Intelligence.

DECLARATION OF GENERATIVE AI AND AI-ASSISTED TECHNOLOGIES IN THE WRITING PROCESS

During the preparation of this work, the authors used ChatGPT in order to improve readability and language. After using this tool/service, the authors reviewed and edited the content as needed and take full responsibility for the content of the publication.

SUPPLEMENTAL INFORMATION

Supplemental information can be found online at https://doi.org/10.1016/j.patter.2026.101520.

Received: November 6, 2025
Revised: January 5, 2026
Accepted: February 26, 2026
Published: March 30, 2026

REFERENCES

1. Silver, D., Schrittwieser, J., Simonyan, K., Antonoglou, I., Huang, A., Guez, A., Hubert, T., Baker, L., Lai, M., Bolton, A., et al. (2017). Mastering the game of Go without human knowledge. Nature *550*, 354–359.
2. Achiam, J., Adler, S., Agarwal, S., Ahmad, L., Akkaya, I., Aleman, F.L., Almeida, D., Altenschmidt, J., Altman, S., Anadkat, S., et al. (2023). GPT-4 technical report. Preprint at arXiv. https://doi.org/10.48550/arXiv.2303.08774.
3. Wang, Y., and Rubel, E.W. (2012). In vivo reversible regulation of dendritic patterning by afferent input in bipolar auditory neurons. J. Neurosci. *32*, 11495–11504.
4. Benavides-Piccione, R., Regalado-Reyes, M., Fernaud-Espinosa, I., Kastanauskaite, A., Tapia-González, S., León-Espinosa, G., Rojo, C., Insausti, R., Segev, I., and DeFelipe, J. (2020). Differential structure of hippocampal ca1 pyramidal neurons in the human and mouse. Cereb. Cortex *30*, 730–752.
5. Adusei, M., Hasse, J.M., and Briggs, F. (2021). Morphological evidence for multiple distinct channels of corticogeniculate feedback originating in mid-level extrastriate visual areas of the ferret. Brain Struct. Funct. *226*, 2777–2791.
6. Ascoli, G.A., Donohue, D.E., and Halavi, M. (2007). Neuromorpho.org: a central resource for neuronal morphologies. J. Neurosci. *27*, 9247–9251.
7. Stuart, G., Spruston, N., and Häusser, M. (2016). Dendrites (Oxford University Press).
8. Chklovskii, D.B. (2000). Optimal sizes of dendritic and axonal arbors in a topographic projection. J. Neurophysiol. *83*, 2113–2119.
9. Chklovskii, D.B. (2004). Synaptic connectivity and neuronal morphology: two sides of the same coin. Neuron *43*, 609–617.
10. Poirazi, P., Brannon, T., and Mel, B.W. (2003). Pyramidal Neuron as Two-Layer Neural Network. Neuron *37*, 989–999.
11. Magee, J.C. (2000). Dendritic integration of excitatory synaptic input. Nat. Rev. Neurosci. *1*, 181–190.
12. Major, G., Larkum, M.E., and Schiller, J. (2013). Active properties of neocortical pyramidal neuron dendrites. Annu. Rev. Neurosci. *36*, 1–24.
13. Poirazi, P., and Mel, B.W. (2001). Impact of Active Dendrites and Structural Plasticity on the Memory Capacity of Neural Tissue. Neuron *29*, 779–796.
14. Jones, I.S., and Kording, K.P. (2021). Might a single neuron solve interesting machine learning problems through successive computations on its dendritic tree? Neural Comput. *33*, 1554–1571.
15. Richards, B.A., and Lillicrap, T.P. (2019). Dendritic solutions to the credit assignment problem. Curr. Opin. Neurobiol. *54*, 28–36.
16. Baronig, M., and Legenstein, R. (2023). Context association in pyramidal neurons through local synaptic plasticity in apical dendrites. Front. Neurosci. *17*, 1276706.
17. Mao, J., Zheng, H., Yin, H., Fan, H., Mei, L., Guo, H., Li, Y., Wu, J., Pei, J., and Deng, L. (2026). Adaptive dendritic plasticity in brain-inspired dynamic neural networks for enhanced multi-timescale feature extraction. Neural Netw. *194*, 108191.
18. Wu, X., Liu, X., Li, W., and Wu, Q. (2018). Improved expressivity through dendritic neural networks. In Proceedings of the 32nd International Conference on Neural Information Processing Systems (NIPS) (Curran Associates Inc.), pp. 8068–8079.
19. Dally, W. (2022). On the model of computation: point: We Must Extend Our Model of Computation to Account for Cost and Location. Commun. ACM *65*, 30–31.
20. Levy, W.B., and Calvert, V.G. (2021). Communication consumes 35 times more energy than computation in the human cortex, but both costs are needed to predict synapse number. Proc. Natl. Acad. Sci. USA *118*, e2008173118.
21. Chavlis, S., and Poirazi, P. (2025). Dendrites endow artificial neural networks with accurate, robust and parameter-efficient learning. Nat. Commun. *16*, 943.
22. Polsky, A., Mel, B.W., and Schiller, J. (2004). Computational subunits in thin dendrites of pyramidal cells. Nat. Neurosci. *7*, 621–627.
23. Mota, B., Dos Santos, S.E., Ventura-Antunes, L., Jardim-Messeder, D., Neves, K., Kazu, R.S., Noctor, S., Lambert, K., Bertelsen, M.F., Manger, P.R., et al. (2019). White matter volume and white/gray matter ratio in mammalian species as a consequence of the universal scaling of cortical folding. Proc. Natl. Acad. Sci. USA *116*, 15253–15261.
24. Attwell, D., and Laughlin, S.B. (2001). An energy budget for signaling in the grey matter of the brain. J. Cerebr. Blood Flow Metabol. *21*, 1133–1145.
25. Yu, Y., Herman, P., Rothman, D.L., Agarwal, D., and Hyder, F. (2018). Evaluating the gray and white matter energy budgets of human brain function. J. Cerebr. Blood Flow Metabol. *38*, 1339–1353.
26. He, K., Zhang, X., Ren, S., and Sun, J. (2016). Deep residual learning for image recognition. In Proceedings of the IEEE conference on computer vision and pattern recognition (CVPR) (IEEE), pp. 770–778.
27. Braitenberg, V., and Schüz, A. (2013). Cortex: Statistics and Geometry of Neuronal Connectivity (Springer Science & Business Media).
28. Chakrabarti, A., and Moseley, B. (2019). Backprop with approximate activations for memory-efficient network training. In Proceedings of the 33rd International Conference on Neural Information Processing Systems (CVPR) (Curran Associates Inc.), pp. 2429–2438.
29. LeCun, Y., Bottou, L., Bengio, Y., and Haffner, P. (1998). Gradient-based learning applied to document recognition. Proc. IEEE *86*, 2278–2324.
30. Goodfellow, I., Warde-Farley, D., Mirza, M., Courville, A., and Bengio, Y. (2013). Maxout networks. In International conference on machine learning (ICML) (PMLR), pp. 1319–1327.
31. Shazeer, N., Mirhoseini, A., Maziarz, K., Davis, A., Le, Q., Hinton, G., and Dean, J. (2017). Outrageously large neural networks: The sparsely-gated mixture-of-experts layer. Preprint at arXiv. https://doi.org/10.48550/arXiv.1701.06538.
32. Naud, R., and Sprekeler, H. (2018). Sparse bursts optimize information transmission in a multiplexed neural code. Proc. Natl. Acad. Sci. USA *115*, E6329–E6338.
33. Sezener, E., Grabska-Barwińska, A., Kostadinov, D., Beau, M., Krishnagopal, S., Budden, D., Hutter, M., Veness, J., Botvinick, M., Clopath, C., et al. (2021). A rapid and efficient learning rule for biological neural circuits. Preprint at bioRxiv. https://doi.org/10.1101/2021.03.10.434756.
34. Iyer, A., Grewal, K., Velu, A., Souza, L.O., Forest, J., and Ahmad, S. (2022). Avoiding catastrophe: Active dendrites enable multi-task learning in dynamic environments. Front. Neurorob. *16*, 846219.
35. Watts, D.J., and Strogatz, S.H. (1998). Collective dynamics of 'small-world'networks. Nature *393*, 440–442.
36. Latora, V., and Marchiori, M. (2001). Efficient behavior of small-world networks. Phys. Rev. Lett. *87*, 198701.
37. Deng, J., Dong, W., Socher, R., Li, L.-J., Li, K., and Fei-Fei, L. (2009). Imagenet: A large-scale hierarchical image database. In 2009 IEEE conference on computer vision and pattern recognition (CVPR) (IEEE), pp. 248–255.
38. Krizhevsky, A., and Hinton, G. (2009). Learning multiple layers of features from tiny images. Tech. Rep.
39. Panayotov, V., Chen, G., Povey, D., and Khudanpur, S. (2015). Librispeech: an asr corpus based on public domain audio books. In

2015 IEEE international conference on acoustics, speech and signal processing (ICASSP) (IEEE), pp. 5206–5210.

40. Steele, J.M., and Snyder, T.L. (1989). Worst-case growth rates of some classical problems of combinatorial optimization. SIAM J. Comput. *18*, 278–287.
41. Akopyan, F., Sawada, J., Cassidy, A., Alvarez-Icaza, R., Arthur, J., Merolla, P., Imam, N., Nakamura, Y., Datta, P., Nam, G.-J., et al. (2015). Truenorth: Design and tool flow of a 65 mw 1 million neuron programmable neurosynaptic chip. IEEE Trans. Comput. Aided Des. Integrated Circ. Syst. *34*, 1537–1557.
42. Davies, M., Srinivasa, N., Lin, T.-H., Chinya, G., Cao, Y., Choday, S.H., Dimou, G., Joshi, P., Imam, N., Jain, S., et al. (2018). Loihi: A neuromorphic manycore processor with on-chip learning. IEEE Micro *38*, 82–99.
43. Ma, D., Jin, X., Sun, S., Li, Y., Wu, X., Hu, Y., Yang, F., Tang, H., Zhu, X., Lin, P., and Pan, G. (2024). Darwin3: a large-scale neuromorphic chip with a novel isa and on-chip learning. Natl. Sci. Rev. *11*, nwae102.
44. Wu, X., Zhao, P., Yu, Z., Ma, L., Gao, Y., Yip, K.-W., Tang, H., Pan, G., Poirazi, P., and Huang, T. Code for the paper "Dendritic Nonlinearities Mitigate Communication Costs" (2026). https://doi.org/10.6084/m9.figshare.31305517.

Patterns, Volume *7*

Supplemental information

Dendritic nonlinearities mitigate communication costs

Xundong Wu, Pengfei Zhao, Zilin Yu, Lei Ma, Yifan Gao, Ka-Wa Yip, Huajin Tang, Gang Pan, Panayiota Poirazi, and Tiejun Huang

Supplemental Notes

S1 Proof of Theorem S1

As illustrated in Eq. S1, the left-hand term—representing the information from the pooled neuron output—is bounded by the information present in the dendrites being pooled. Following shows a comprehensive proof of this theorem.

Theorem S1. *The entropy of the sum (neuron output) of random variables (dendritic outputs)* $\mathsf{d}_1, \mathsf{d}_2, \ldots, \mathsf{d}_K$ *is less than or equal to the joint entropy of these random variables. The relation between the two is given as:*

$$H\left(\sum_{j=1}^{K} \mathsf{d}_j\right) = H(\mathsf{d}_1, \ldots, \mathsf{d}_K) - H\left(\mathsf{d}_1, \ldots, \mathsf{d}_K \mid \sum_{j=1}^{K} \mathsf{d}_j\right) . \tag{S1}$$

In order to prove Theorem S1, we first prove the following lemma.

Lemma S1. *The conditional entropy of* $\sum_{j=1}^{K} \mathsf{d}_j$ *given* $\mathsf{d}_1, \mathsf{d}_2, \ldots, \mathsf{d}_K$ *is zero, i.e.,*

$$H\left(\sum_{j=1}^{K} \mathsf{d}_j \mid \mathsf{d}_1, \mathsf{d}_2, \ldots, \mathsf{d}_K\right) = 0 . \tag{S2}$$

Proof. If the values of $\mathsf{d}_1, \mathsf{d}_2, \ldots, \mathsf{d}_K$ are known, then the value of $\sum_{j=1}^{K} \mathsf{d}_j$ is also known. Therefore, the statement is intuitively true. For discrete random variables d_i, a formal proof can be presented as follows.

$$\begin{aligned} & H\left(\sum_{j=1}^{K} \mathsf{d}_j \mid \mathsf{d}_1, \mathsf{d}_2, \ldots, \mathsf{d}_K\right) \\ &= \sum_{d_1, \ldots, d_K} p(d_1, \ldots, d_K) H\left(\sum_{j=1}^{K} \mathsf{d}_j \mid \mathsf{d}_1 = d_1, \ldots, \mathsf{d}_K = d_K\right) \\ &= \sum_{d} 0 = 0 . \end{aligned} \tag{S3}$$

□

We can now prove Theorem S1, by first making use of a relationship between joint entropy and conditional entropy[S1].

Proof of Theorem S1: The joint entropy of $\mathsf{d}_1, \mathsf{d}_2, \ldots, \mathsf{d}_K$ and $\sum_{j=1}^{K} \mathsf{d}_j$ is:

$$\begin{aligned} & H\left(\mathsf{d}_1, \ldots, \mathsf{d}_K, \sum_{j=1}^{K} \mathsf{d}_j\right) \\ &= H\left(\sum_{j=1}^{K} \mathsf{d}_j\right) + H\left(\mathsf{d}_1, \ldots, \mathsf{d}_K \mid \sum_{j=1}^{K} \mathsf{d}_j\right) \\ &= H(\mathsf{d}_1, \ldots, \mathsf{d}_K) + H\left(\sum_{j=1}^{K} \mathsf{d}_j \mid \mathsf{d}_1, \ldots, \mathsf{d}_K\right) . \end{aligned} \tag{S4}$$

Therefore,

$$\begin{aligned} & H\left(\sum_{j=1}^{K} \mathsf{d}_j\right) \\ &= H(\mathsf{d}_1, \ldots, \mathsf{d}_K) + H\left(\sum_{j=1}^{K} \mathsf{d}_j \mid \mathsf{d}_1, \ldots, \mathsf{d}_K\right) - H\left(\mathsf{d}_1, \ldots, \mathsf{d}_K \mid \sum_{j=1}^{K} \mathsf{d}_j\right) \\ &= H(\mathsf{d}_1, \ldots, \mathsf{d}_K) - H\left(\mathsf{d}_1, \ldots, \mathsf{d}_K \mid \sum_{j=1}^{K} \mathsf{d}_j\right) \qquad (\textit{From Lemma S1.}) \end{aligned} \tag{S5}$$

□

Since the conditional entropy $H\left(\mathsf{d}_1, \ldots, \mathsf{d}_K \mid \sum_{j=1}^{K} \mathsf{d}_j\right)$ is non-negative, the upper bound of $H\left(\sum_{j=1}^{K} \mathsf{d}_j\right)$ is $H(\mathsf{d}_1, \mathsf{d}_2, \ldots, \mathsf{d}_K)$.

S2 Computing and parametric complexity of models

Table S1: Complexity data on typical models

Dendrites/neuron (K)	$\Psi = 1/\sqrt{K}$	Computing complexity (MMACs)	# of Parameters
1 (Resnet-18)	1	1,821.63	11,689,512
4	1/2	1,804.34	11,556,200
16	1/4	1,799.65	11,521,800
64	1/16	1,799.37	11,512,664

Table S1 shows the computing and parametric complexity comparison of models from the light-blue dashed curve of Fig. 3. We use customized THOP package to calculate the model complexity data where we count two sum operations as one MAC operation.

S3 Derivation of Communication Costs for PE Mesh Architecture

S3.1 Point neurons based model

Our analysis initiates with a model composed of point neurons. As previously mentioned, our investigation focuses on two network layers. We assume that the first layer sends an output of D dimensions to the second layer. For convenience and without loss of generality, we assume that each of the D dimensions originates from one PE on the chip.

In order to arrange D PEs on die area of size 1×1, each PE must have a height and width of $l = 1/\sqrt{D}$, resulting in an area size of $1/D$. Similarly, the second layer is also composed of D PEs of the same size. Consequently, we obtain a grid of N by N PEs with $N = \sqrt{D}$, with a distance of l between the center of each pair of neighboring PEs. See Fig. S1-A for a visual illustration.

For this arrangement we have

$$C_A = D(\sqrt{D} - 1)l = D - \sqrt{D}\,, \tag{S6}$$

as measured with Manhattan distance. Furthermore, an illustrative example of signal propagation within this context is provided in Fig. S1-B. Derivation of Eq. S6 can be found in Supplemental S3.3.

We assess C_E with minimal rectilinear spanning tree (MRST) algorithm[S2]. Given a grid of $N \times N$ PEs, the objective is to deliver every dimension of the data to each PE. The MRST algorithm enables us to determine the minimal path length required to connect all PEs, which is $(N^2 - 1) \cdot l$. An example path is illustrated in Fig. S1-C.

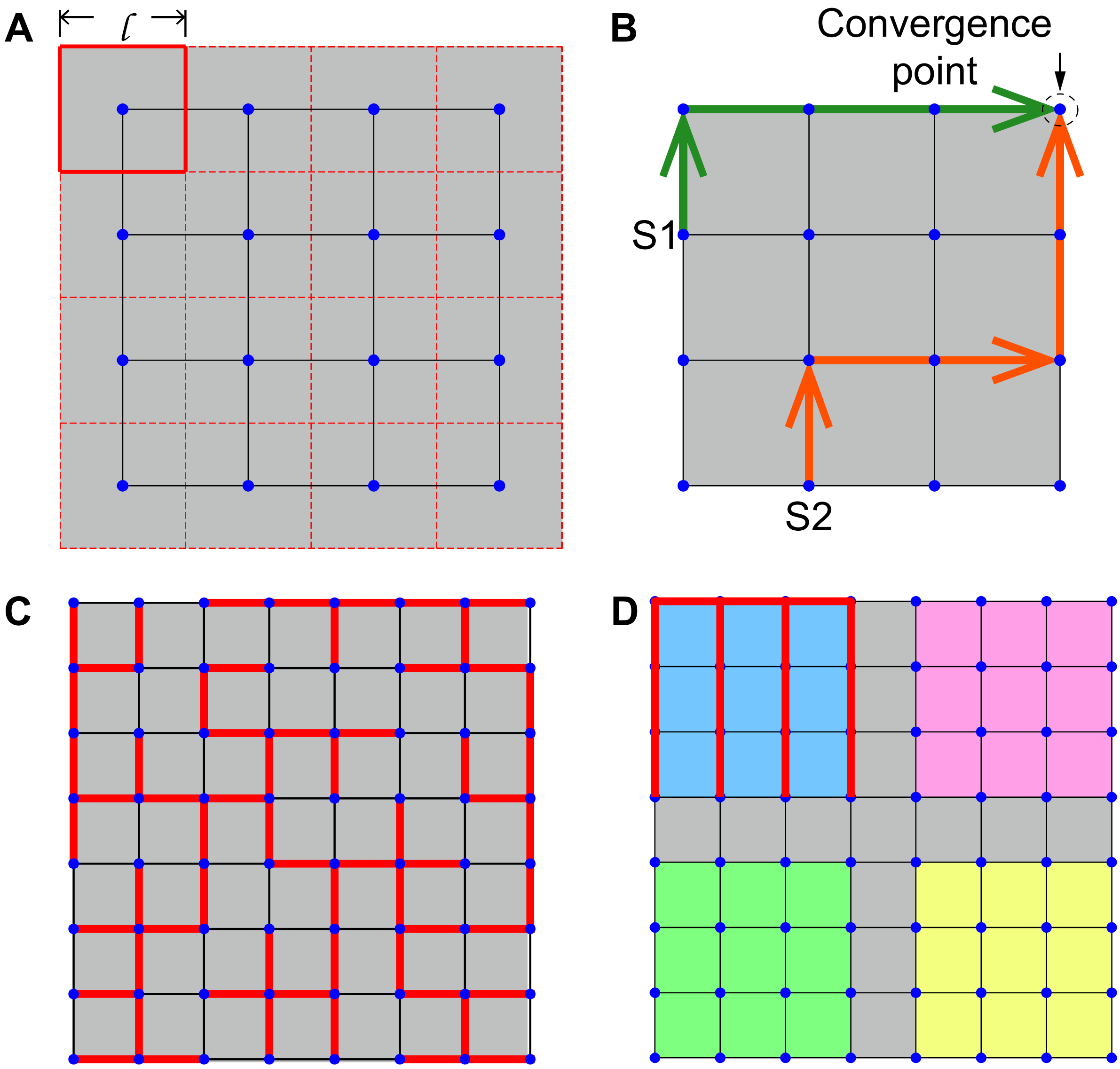

Figure S1: Illustration of the Processing Element (PE) Grid and Communication Path Models for Point and Dendritic Neurons.
(A) Showcases a 16-unit grid of processing elements (PEs), where each PE has a side length, l, computed as $l = 1/\sqrt{D}$ or $1/4$ in this example. The boundary of top-left PE is emphasized with solid red lines. For improved clarity, only the grid of central points will be displayed henceforth.(B) Depicts two city-walk paths originating from S1 (green path) and S2 (orange path) leading to a convergence point. The green path has a total length of $4l$, while the red path spans $5l$ in length. (C) Demonstrates a city-walk path with red lines, connecting all points on an 8×8 grid. This route enables data dissemination across the target set with minimal cost. (D) Illustrates four groups of PEs, each color-coded to represent a dendritic neuron with 16 dendrites. Within each group, dendritic outputs are combined to generate a single output. The aggregation path can be assessed using the MRST algorithm, with an example path displayed in the top-left block.

Consequently, we obtain the cost of delivering data as 40

$$C_E = (N^2 - 1) \cdot l \cdot D = (D - 1)\sqrt{D}\,. \quad \text{(S7)}$$

S3.2 Dendritic neuron based model

As earlier we maintain the number of parameters and floating-point operations (FLOPs) consistent with those in the point neuron model scenario. That is, given that each neuron has K dendrites, one layer of the model under examination will have a total of $M = D\sqrt{K}$ dendrites. As illustrated in Fig. S1-D, every group of K dendrites aggregates to form a single output dimension. Consequently, the first layer will produce an output with a dimensionality of $\hat{D} = D/\sqrt{K}$, which serves to maintain an equivalent computational complexity as the point neuron-based model previously described. We reiterate our assumption that those $\hat{D}$ neurons are arranged in a grid format, specifically of size $\hat{N} \times \hat{N}$, with $\hat{N} = \sqrt{\hat{D}}$.

We postulate that the computation of each dendrite is processed by one PE. In this scenario, the die area is divided into M units, with each unit occupying a specific area. The height and width of this area, denoted by $\hat{l}$, can be calculated as $\hat{l} = 1/\sqrt{M}$. Through this, we arrive at the size of a PE for processing each dendrite being $\frac{1}{D\sqrt{K}}$, which is $1/\sqrt{K}$ of the point neuron-based model PE die size. This corresponds to the assumption that a dendrite in this analysis receives a proportion of $1/\sqrt{K}$ of the inputs that a point neuron receives.

In light of the aforementioned derivation, we note that the signal transfer cost, denoted as $\hat{C}_A$, consists of two components. The first component, $\hat{C}_{AG}$, refers to the cost of aggregating dendritic outputs for each neuron. The second component, $\hat{C}_{AA}$, represents the cost of transmitting the aggregated data of all neurons off the die. Their expressions are as follows.

$$\hat{C}_A = \hat{C}_{AG} + \hat{C}_{AA} < \sqrt{D}K^{1/4} + \frac{D}{\sqrt{K}}\,. \tag{S8}$$

Please see S3.4 for the derivation.

In congruence with the approach adopted for dense models, we also employ the MRST algorithm to estimate the communication cost when dealing with sparse models. Considering the variability in the communication cost due to different sparse connection patterns, we sample a set of 100 random connection patterns for each setting to provide a robust estimate of the average cost. Akin to the point neuron models, we will not attempt to derive $\hat{C}_I$, although we have the relationship of $D = \sqrt{K} \cdot \hat{C}_I$ under the assumptions of the equivalent parameter/FLOPs count setting.

As for the $\hat{C}_E$ component, note that the second layer receives $\frac{D}{\sqrt{K}}$ inputs and consists of M units. Utilizing the MRST method, the cost associated with one-dimensional input connecting to M units can be computed as $(M-1)\cdot\hat{l}$. We arrive at

$$\hat{C}_E = \frac{D}{\sqrt{K}}(D\sqrt{K} - 1) \cdot \hat{l} \approx D^{\frac{3}{2}}/K^{\frac{1}{4}}\,. \tag{S9}$$

S3.3 Derivation of Eq. S6

For simplicity, we place the inter-chip communication junction point at the top-right corner, in the 0-th row and column. It starts with ID 0, counting from right to left and top to bottom. Therefore, the total cost of propagating outputs from every PE to the junction point is:

$$\begin{aligned}
C_A &= \left(\sum_{x,y=0}^{N-1}(x+y)\right) l \\
&= \left(\sum_{x=0}^{N-1} x \sum_{y=0}^{N-1} 1 + \sum_{x=0}^{N-1} 1 \sum_{y=0}^{N-1} y\right) l \\
&= \left(\frac{(N-1)N}{2}N + N\frac{(N-1)N}{2}\right) l \\
&= N^2(N-1)l \\
&= D(\sqrt{D}-1)\frac{1}{\sqrt{D}} \\
&= D - \sqrt{D}\,.
\end{aligned} \tag{S10}$$

S3.4 Derivation of Eq. S8

$$\begin{aligned}\hat{C}_{AG} &= (K-1)\cdot\hat{D}\cdot\hat{l}\\ &= \sqrt{D}(K^{1/4}-K^{-3/4}) < \sqrt{D}K^{1/4}\,, \end{aligned} \tag{S11}$$

$$\hat{C}_{AA} = \hat{N}\hat{N}(\hat{N}-1)\hat{l}(\sqrt{K}) < \frac{D}{\sqrt{K}}\,, \tag{S12}$$

$$\hat{C}_{A} = \hat{C}_{AG} + \hat{C}_{AA} < \sqrt{D}K^{1/4} + \frac{D}{\sqrt{K}}\,. \tag{S13}$$

S4 Communication cost Analysis for block-wise GEMM computation on GPU

S4.1 Theoretical analysis

In this section, we analyze how the adoption of the proposed dendritic structure affects communication costs during neural network inference when compared to a point neuron-based structure on typical GPU-like architectures.

For this part of analysis we follow the notation used by the GPU community as in CUTLASS[S3], which differs from the notation used in the rest of the manuscript.

First, we delineate our setting, assuming a feed-forward network layer. For the standard model, the computation of the layer can be expressed as

$$\boldsymbol{C}^f = \sigma(\boldsymbol{A}\cdot\boldsymbol{B}),$$

where σ represents the element-wise nonlinear output function. For clarity, we omit the bias term.

The computational complexity of the nonlinear function σ is relatively small compared to that of the matrix multiplication. Therefore, our focus will be on the matrix multiplication

$$\boldsymbol{C} = \boldsymbol{A}\cdot\boldsymbol{B}.$$

And we have $\boldsymbol{A}\in\mathbb{R}^{M\times L}$, $\boldsymbol{B}\in\mathbb{R}^{L\times N}$, $\boldsymbol{C}\in\mathbb{R}^{M\times N}$.

Similarly, for the dendritic model, we have

$$\hat{\boldsymbol{C}}^f = \sigma(\hat{\boldsymbol{A}}\cdot\hat{\boldsymbol{B}})$$

before the dendritic aggregation process. The dendritic layer output $\hat{\boldsymbol{C}}^o$ is then computed as

$$\hat{C}^o_{i,j} = \sum_{s=1}^{K}\hat{C}_{i,(j-1)K+s}$$

, where K is the number of dendrites per neuron. We have $\hat{\boldsymbol{A}}\in\mathbb{R}^{M\times L/\sqrt{K}}$, $\hat{\boldsymbol{B}}\in\mathbb{R}^{L\times N\sqrt{K}}$, $\hat{\boldsymbol{C}}\in\mathbb{R}^{M\times N\sqrt{K}}$. As described above, we reduce every K neighboring elements along N dimensions in $\hat{\boldsymbol{C}}^f$ into one element, namely the output of the dendritic layer $\hat{\boldsymbol{C}}^o$. Given that dendritic aggregation can be performed locally with low cost and the computing complexity of nonlinear functions is small. We again focus on the core matrix multiplication part described as $\hat{\boldsymbol{C}} = \hat{\boldsymbol{A}}\cdot\hat{\boldsymbol{B}}$. Though the aggregation process is important for reducing output communication cost.

To understand the communication cost we need to get some idea about the memory hierarchy of a GPU. The architecture depicted in the Fig. S2 represents a simplified illustration of a typical GPU processor such as Nvidia A100/H100. In this architecture, the global memory is a central storage resource accessible to all processing elements (PEs) within the processor. The PE units here correspond to Streaming Multiprocessors (SMs) of GPUs. To reduce the communication cost of accessing the high latency global memory, there is also an on-chip L2 cache that accelerates data I/O of PEs. Each PE is equipped with its own private shared memory, which is utilized by the tensor cores housed within that PE. Each tensor core within a PE is further equipped with its own private registers for localized computing, ensuring rapid access to frequently used data and further optimizing computational efficiency.

To avoid diving too deep into GEMM optimization, we refer readers to the CUTLASS documentation for introduction on block-wise general matrix multiply (GEMM)[S3].

The pseudo code for the baseline GEMM is shown in Alg. 1. We first calculate communication complexity of the baseline models without considering L2 cache.

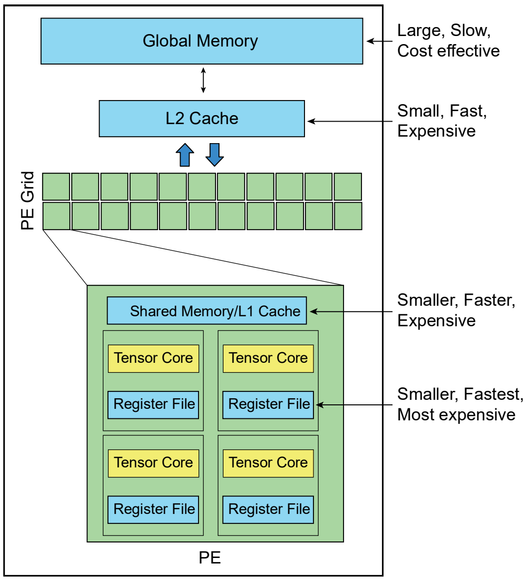

Figure S2: Simplified architecture of a GPU processor. The global memory, characterized by being large, slower, and cost-effective, is accessible by all processing elements (PEs) in the processor[S3,S4]. On chip L2 cache enable much faster access to data accessed by PE units. Each PE contains its own private shared memory, which is smaller, faster, and more costly, shared by the tensor cores within that PE. Each tensor core has its own private register file, which is the smallest, fastest, and associated with low communication cost.

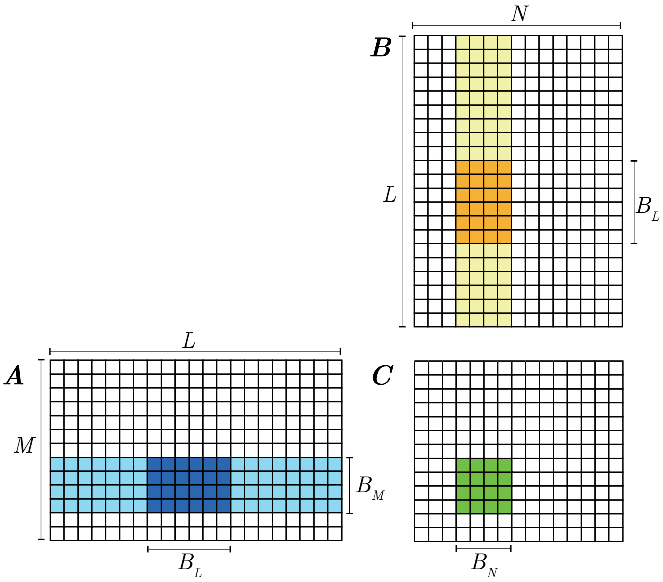

Figure S3: Block-wise General Matrix-Matrix Multiplication (GEMM) illustration.
This figure demonstrates the multiplication of matrices $\boldsymbol{C} = \boldsymbol{A} \cdot \boldsymbol{B}$, where $\boldsymbol{A} \in \mathbb{R}^{M \times L}$, $\boldsymbol{B} \in \mathbb{R}^{L \times N}$, and $\boldsymbol{C} \in \mathbb{R}^{M \times N}$. The computation is divided into blocks to optimize performance and efficiency.

From Fig. S3, for each block in $\boldsymbol{C}$ of size B_M by B_N, we need to read $L(B_M + B_N)$ units of data from global memory. Therefore for computation of the whole $\boldsymbol{C}$ we need to read

$$(B_M + B_N) \cdot L \cdot \frac{M}{B_M} \cdot \frac{N}{B_N}$$

units of data. To write the result matrix $\boldsymbol{C}$ to the global memory, the write cost is

$$M \cdot N.$$

For a dendritic model with K dendrites per neuron, we have $\hat{\boldsymbol{A}} \in \mathbb{R}^{M\times L/\sqrt{K}}$, $\hat{\boldsymbol{B}} \in \mathbb{R}^{L\times N\sqrt{K}}$, $\hat{\boldsymbol{C}} \in \mathbb{R}^{M\times N\sqrt{K}}$. With this new matrix dimensionality we have the following memory read cost for the dendritic model:

$$(B_M + B_N) \cdot \frac{L}{\sqrt{K}} \cdot \frac{M}{B_M} \cdot \frac{N \cdot \sqrt{K}}{B_N} = (B_M + B_N) \cdot L \cdot \frac{M}{B_M} \cdot \frac{N}{B_N}.$$

This is the same as the point neuron based model. As for the write cost, because we reduce elements in $\hat{\boldsymbol{C}}$ by group of K, we have a writing cost of :

$$\frac{M \cdot N}{\sqrt{K}}.$$

From the above analysis, it is evident that with the same B_M, B_N combinations for equivalent point neuron and dendritic neuron based network layers, there is no difference in the total memory read cost. The memory write cost can be reduced by $\sqrt{K}$. This analysis may suggest that adopting dendritic structure can only lead to minor reduction in communication cost given memory read is much larger than memory write.

The key is to coordinate block processing properly to take advantage of the L2 cache. For this part we refer the reader to the "L2 cache optimization" section of the Triton GEMM tutorial[S5] and CUTLASS documentation[S3] for background information.

To improve computational efficiency, it is beneficial to take advantage of the sharing of data among neighboring blocks of the matrix $\boldsymbol{C}$. This is achieved by computing several adjacent rows of $\boldsymbol{C}$, which correspond to the same rows in the matrix $\boldsymbol{A}$, as a group. This grouped computation strategy allows for the reuse of input blocks from matrices $\boldsymbol{A}$ and $\boldsymbol{B}$, minimizing data reloading and maximizing cache utilization. After completing the computation for one group, the process then transitions to another group. This approach not only streamlines data access patterns but also significantly reduces memory overhead and improves overall performance of GEMM computation.

Here we provide a simplified analysis on how dendritic architecture can help improve L2 cache hit rate therefore reduce communication cost on global memory access. Due to the complexity of the hierarchy cache mechanism, this analysis is not intended to be precise, but rather to help provide a theoretical understanding.

Assume that we form a block group of G rows of blocks from $\boldsymbol{A}$ according to the standard approach to improve the efficiency of L2 cache[S3,S5]. For this to work, we need to fit the block of $G \cdot B_M$ rows and a single B_N column into the L2 cache. We denote the capacity of the L2 cache as Q. That is, we have

$$(G \cdot B_M + B_N) \cdot L = Q.$$

It is possible to put multiple columns from the $\boldsymbol{B}$ matrix in the cache, but we can consider that it is absorbed in B_N. To utilize the cache efficiently, $G \cdot B_M$ need to stay in the cache while computations are performed along the N axis[S5] where each step of computation requires read B_N columns from the memory. In this way we can calculate that the memory read cost on matrix $\boldsymbol{B}$ part is

$$\frac{N \cdot L \cdot M}{B_M \cdot G}.$$

And the read cost on $\boldsymbol{A}$ part is $M \cdot L$, the total read cost would be

$$\frac{N \cdot L \cdot M}{Q/L - B_N} + M \cdot L.$$

It is desirable to set B_N to a small value. Therefore the read cost will roughly be equal to

$$\frac{N \cdot L^2 \cdot M}{Q} + M \cdot L.$$

For models with dendritic neurons of K dendrites, we will have a read cost of

$$\frac{N \cdot L^2 \cdot M}{(Q\sqrt{K})} + M \cdot L/\sqrt{K} = (\frac{N \cdot L^2 \cdot M}{Q} + M \cdot L)/\sqrt{K}.$$

Therefore, we can significantly reduce global memory read access through adopting dendritic structure.

Algorithm 1 Block-wise GEMM (Modified from the original algorithm from[S5])

Input: Matrices $\boldsymbol{A} \in \mathbb{R}^{M\times L}$, $\boldsymbol{B} \in \mathbb{R}^{L\times N}$, $\boldsymbol{C} \in \mathbb{R}^{M\times N}$
Output: Matrix $\boldsymbol{C}$ containing the result of $\boldsymbol{C} = \boldsymbol{A} \times \boldsymbol{B}$
Define: Block sizes B_M, B_N, B_L
for each m in 0 to M by B_M **do** ▷ Parallel execution over blocks of C
    for each n in 0 to N by B_N **do** ▷ Parallel execution over blocks of C
        Initialize $acc \leftarrow$ zeros(B_M, B_N)
        for each l in 0 to L by B_L **do** ▷ Iterate over blocks of A and B
            $a\_block \leftarrow \boldsymbol{A}[m : m + B_M, l : l + B_L]$
            $b\_block \leftarrow \boldsymbol{B}[l : l + B_L, n : n + B_N]$
            $acc \leftarrow acc + (a\_block \times b\_block)$
        end for
        $\boldsymbol{C}[m : m + B_M, n : n + B_N] \leftarrow acc$
    end for
end for

S4.2 Empirical analysis

The theoretical analysis presented above incorporates certain assumptions, such as a two-layer memory structure and explicit cache control, that do not fully align with the architecture of real-world hardware. To validate and extend this analysis, we conducted an empirical study of memory access costs during the inference process of typical neural network layers on an Nvidia A40 GPU. The results demonstrate that adopting a dendritic structure can significantly reduce communication costs, in alignment with the predictions of our theoretical framework. Due to the infeasibility of exploring the entire configuration space, this study does not aim to identify the optimal configuration for a specific setting. Instead, it aims to demonstrate clear advantages over well-established baselines that achieve the theoretical lower bound[S5–S7].

We measured the global memory access costs of dendritic neural network layers at three levels of computational complexity. Each baseline configuration used $K = 1$ (one dendrite per neuron), corresponding to a standard feedforward neural network with ReLU nonlinearity. The baseline implementation was built using the Triton standard framework[S3,S5], designed to optimize memory access costs while achieving performance comparable to CuBLAS. In this scenario, computation is represented as $C = \sigma(A \times B)$, where $A \in \mathbb{R}^{M\times L}$, $B \in \mathbb{R}^{L\times N}$, and $C \in \mathbb{R}^{M\times N}$, with σ denoting an element-wise nonlinearity. For all baseline experiments, $M = N = L$ was set to values from $\{1024, 2048, 4096, 8192\}$, representing the four complexity levels. We compared configurations with $K = 1, 4, 16, 64$ dendrites per neuron at each complexity level, ensuring that computational complexity remained consistent across baselines. For instance, at a complexity level of $M = 1024$ and $K = 4$, we set $M = 1024$, $L = 512$, and $N = 2048$. In this case, the final output matrix would have dimensions $\mathbb{R}^{1024\times 512}$ after aggregating the outputs from every four dendrites.

We analyze global memory access patterns using Nvidia Nsight Compute and report the optimal results for each tested configuration. The best results were obtained by searching over $B_M, B_N, B_L \in 16, 32, 64, 128$ (values compatible with the Tensor Core architecture) and $G \in 1, 2, 4, 8, 16, 32, 64, 128, 256$.

Our findings indicate that the dendritic structure offers notable advantages. For smaller layers ($M = 1024$, Fig. S4A), the operator matrices can fit within the L2 cache, leading to lower performance gains from the dendritic structure compared to theoretical predictions (red dashed lines). However, as the matrix size surpasses the L2 cache capacity ($M = 4096$, Fig. S4B), memory access patterns begin to align with the expected $1/\sqrt{K}$ scaling. For reference, the A40 GPU used in these experiments has an L2 cache size of 6,144 KB, as documented in Nvidia Ampere GA102 GPU Architecture white paper. For the largest matrices ($M = 8192$, Fig. S4D), cache evictions lead to memory reads exceeding theoretical predictions.

Similar trends are observed for the write costs, as shown in the middle row of Fig. S4. Note that, in the case of small output matrices, the result matrices may remain in the L2 cache without being transferred to global memory. This can lead to observed write costs smaller than the size of the output matrix. To show a complete picture we also show the optimal total global memory access cost (read+write) measured across different settings shown at the bottom row of Fig. S4.

The observations outlined above highlight a pathway to significantly reduce model inference energy costs by mitigating global memory access complexity. This approach could also facilitate GPU designs with lower memory bandwidth requirements. While reducing communication overhead lowers energy consumption, it does not always

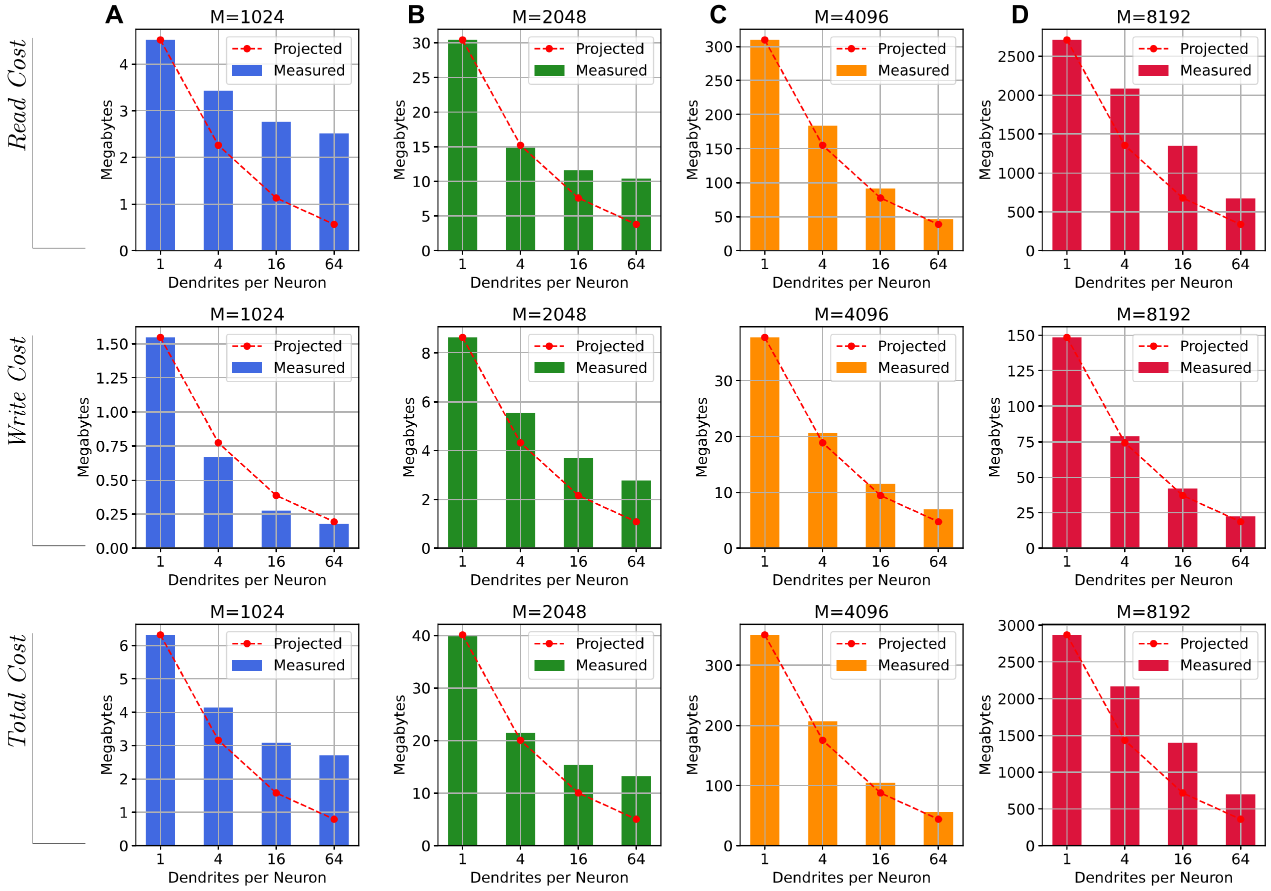

Figure S4: Communication cost analysis across neural network configurations.
Top row: Optimal read costs for networks with varying dendrites per neuron ($K = 1, 4, 16, 64$) at three complexity levels: $M = 1024$ (A), $M = 2048$ (B), $M = 4096$ (C), and $M = 8192$ (D). Bar plots show measured costs, while dotted red lines indicate theoretical scaling projections. **Middle row:** Optimal write costs for the same configurations, showing measured write costs and their theoretical scaling predictions. **Bottom row:** Optimal total communication costs, combining both read and write operations for each configuration.

translate to accelerated model inference due to potential bottlenecks elsewhere in the pipeline. Notably, however, a significant relative performance advantage in network layer inference is observed when the GPU memory is underclocked. In this scenario, where memory I/O emerges as the primary bottleneck, the dendritic model exhibits substantial runtime reductions relative to the baseline, as shown in Fig. S5.

S5 Additional machine learning experimental analysis

S5.1 CIFAR-100 dataset with ResNet-18-style models

We also applied our models to the CIFAR-100 dataset[S8], which comprises 100 distinct object categories and is commonly employed in machine learning studies. Results are shown in Fig. S6 and resemble our findings using the ImageNet dataset, where incorporating dendrites into a model with a fixed inter-layer communication budget consistently yields improved performance. Furthermore, dendritic-based models surpass point-neuron models with the same computational budget, provided that the inter-layer communication budget is above a certain threshold. As in the ImageNet dataset, we used the ResNet-18 model as the baseline architecture.

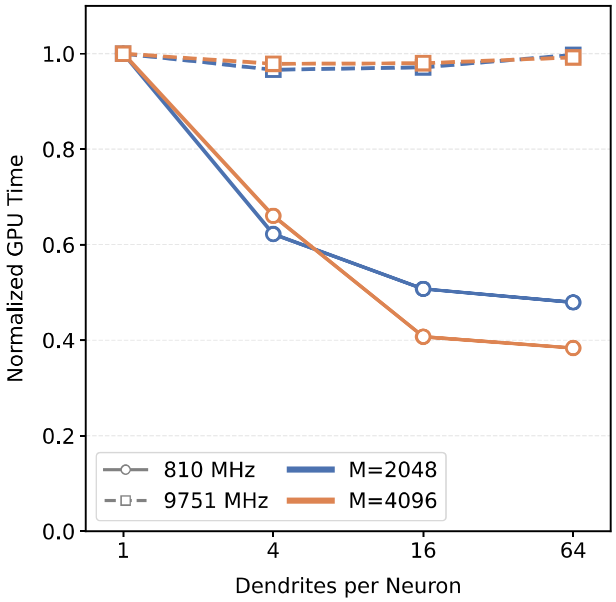

Figure S5: Normalized GPU runtime across varying computational complexities and GPU memory clock speeds. We set baseline $M = N = L$ to values of 2048 and 4096 for these experiments, with the number of dendrites per neuron K varying across $\{1, 4, 16, 64\}$. The GPU runtime is normalized relative to the baseline case for each computational complexity level and clock setting. These experiments were performed on an NVIDIA RTX 3090 GPU, which permits manual adjustment of the memory clock. For this set of experiments, we set the GPU core clock at 1935 MHz and tested memory clock speeds of 810 MHz (memory-constrained) and 9751 MHz (standard), respectively.

S5.2 Additional results on Imagenet dataset experiment

In the results section, we compare the performance of models when they are set to be of same computational complexity level. To obtain a full picture, we also compare models of same number of neurons D. The result is illustrated in Fig. S7. We can observe consistent performance improvement when more dendrites are added to the neurons.

S5.3 Non-Residual Convolutional Neural Network Performance on the ImageNet Dataset

To ensure the robustness of our findings, we also used a convolutional neural network (CNN) model devoid of residual connections[S9]. The base model for this experiment was a modified version of the original ResNet-18 network, from which we eliminated the residual connections. The original ResNet-18 model consists of four stages, each featuring two residual blocks. We removed one residual block from both the second and third stages to reduce computing costs. Fig. S8 illustrates the results from this modified, non-residual network, which are consistent with our original findings shown in Fig. 3.

S5.4 Transformer model

This section investigates the impact of replacing the feedforward block within transformer-based neural networks. The specific feedforward block in question comprises a classic bottleneck architecture, as illustrated in Fig. S9.

A bottleneck structure enhances the expressive capacity of a network module by expanding the number of channels in the middle layer. Conventionally, if the module input consists of L channels, the middle layer is expanded to comprise sL channels. Small integer values are commonly employed for s in typical transformer-based models, with common choices including 2, 3, or 4. Subsequently, the module's output is reduced back to the original L channels.

This bottleneck module confers greater expressivity power to the model than a standard two-layer network of L channels while maintaining a modest input/output channel number for the module. This is similar to what dendritic

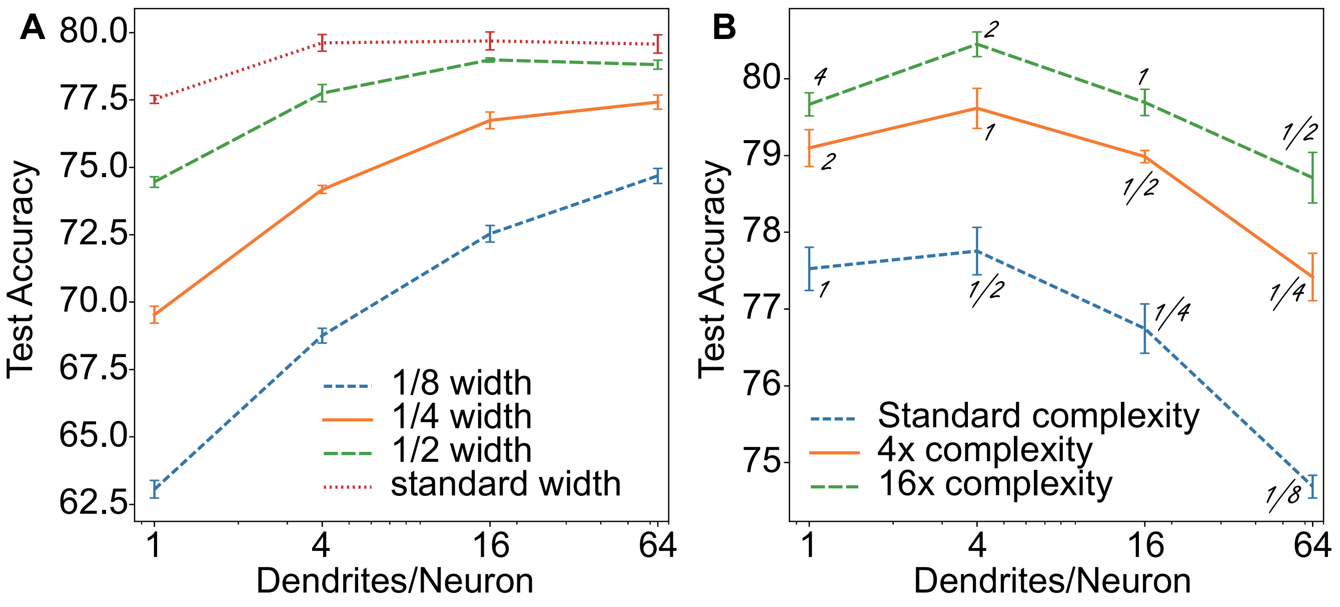

Figure S6: Results on the CIFAR-100 dataset.
Each experiment was performed 5 times, with standard deviations displayed. (A) Test accuracy for models with varying numbers of dendrites per neuron at four distinct levels of network width. (B) Comparison of models with equivalent computational complexities at three different levels. The blue dashed curves represent the baseline ResNet-18 model and subsequent dendritic models with K values of 4, 16, and 64. The orange curve corresponds to models with twice the number of neurons (channels), and the green dashed curve represents models with four times the number of channels. The channel scale factors relative to the standard model are labeled on the curves in (B).

structures try to achieve.

However, the bottleneck structure has an expanded middle layer, necessitating high communication bandwidth. Thus, the question arises: can a dendritic structure supplant the bottleneck structure while conferring additional benefits?

The naive substitution of a bottleneck structure with two dendritic layers is ineffective because the second layer comprises linear neurons. The pooling of linear neuron outputs does not confer inherent advantages to a nonlinear dendritic structure. Consequently, our design only employs a dendritic structure exclusively for the first layer of the block while retaining a linear layer for the second.

More precisely, for a bottleneck structure accepting an input dimension of L and an expansion ratio of s, the corresponding first layer is assigned the dendritic branches equal to $2s - 1$. This configuration maintains the input channel number for both layers at L, preserving the computational and parametric complexity at levels comparable to the original model.

An empirical examination involving a compact transformer model, as proposed by Hassani et al.[S10], demonstrates that this modification incurs only a marginal performance decline. Specifically, test accuracy on the ImageNet dataset decreased from 80.9% to 80.6%, a negligible reduction considering the substantial decrease in peak activation output I/O within the block threefold less than before.

Considering the highly tuned nature of the transformer architecture, we posit that additional refinements to the model—particularly adjustments favoring the dendritic structure may unlock further potential for performance enhancement.

S5.5 Speech recognition task

In addition, we substantiate our theory with a speech recognition task. We employ models trained on the LibriSpeech dataset, which consists of approximately 1,000 hours of spoken English[S11]. Owing to computing resource constraints, we utilize the train-clean-100 and train-clean-360 subsets for model training and the dev-clean subset for model evaluation. The models used in this portion of the experiment are derived from the Jasper model[S12], a 1D convolutional neural network. To lessen the computational burden during model training, we modified the original

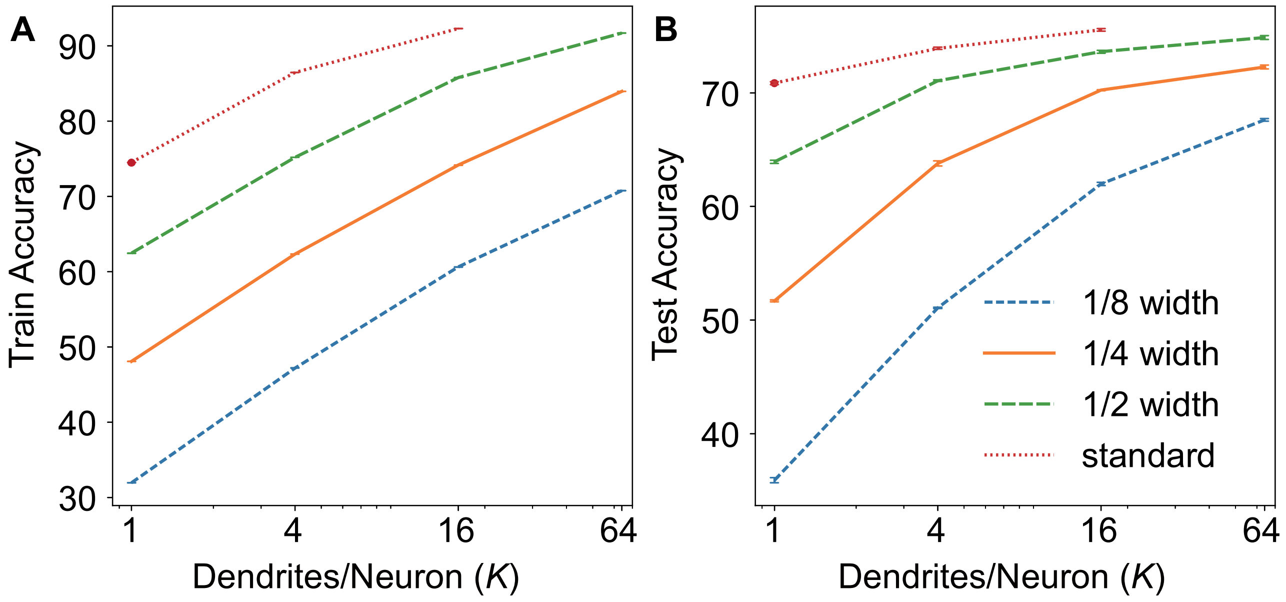

Figure S7: Comparison of Resnet-18-style models composed of point and dendritic neurons trained on the ImageNet dataset.
Each experiment was performed 5 times, with standard deviations displayed. (A) Training accuracy, and (B) Test accuracy for models with varying numbers of dendrites per neuron at four distinct levels of network width. x-axis indicates the number of dendrites per neuron; models with one dendrite per neuron are point neuron-based models.

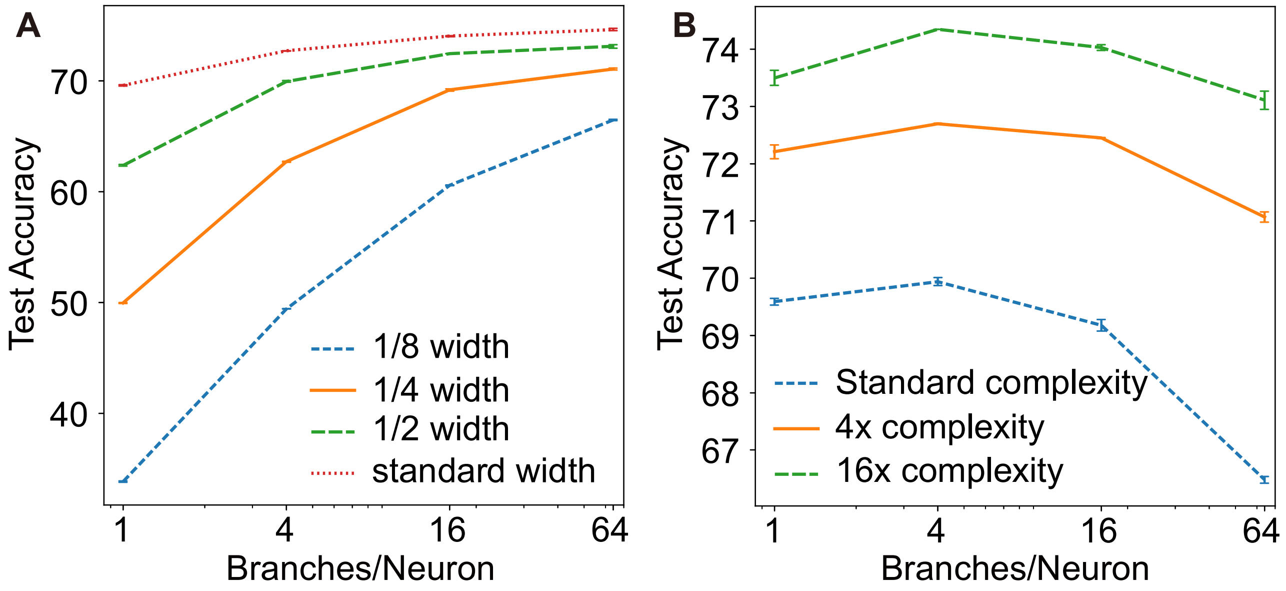

Figure S8: Results on ImageNet dataset using neural network models without residual connections.
Each experiment are performed 3 times, with standard deviations displayed. (A) Test accuracy for models with varying numbers of dendrites per neuron at four distinct levels of network width. (B) Comparison of models with equivalent computational complexities at three different levels. The blue dashed curve represents the baseline and subsequent dendritic models with K values of 4, 16, and 64. The orange curve corresponds to models with twice the number of channels, and the green dashed curve represents four times the number of channels.

model by eliminating the dense residual connections and significantly reducing the number of blocks in the model to arrive at a baseline point neuron based model. Further details regarding the modifications to the models can be found in the accompanying code.

For this part, we carry out two distinct sets of experiments. The first set focuses on models of equivalent computational complexity, and the second emphasizes models sharing the same inter-layer communication cost.

In the first set of experiments, we evaluated models of two distinct computational complexity levels, varying the neuron configurations. Specifically, the configurations encompassed point neurons and dendritic neurons with varying numbers of dendrites. The results for this segment of experiments are displayed in Table S2. Analogous to previous experiments, we observed that models utilizing dendritic neurons were able to achieve comparable performance relative to the point neuron-based models with equivalent computational complexity if they are equipped with efficient inter-layer communication bandwidth.

The second set of experiments is conducted employing models that retain the same inter-layer communication cost. Our experimental procedure begins with a point neuron-based model, which possesses one-fourth of the inter-layer communication complexity compared to the baseline model. This point neuron model is subsequently replaced with dendritic neuron models that contain 4 and 16 dendrites respectively. The corresponding results are systematically presented in Table S3. Upon analyzing these results, it becomes apparent that the performance of the model progressively enhances as we incorporate neurons with an increased number of dendrites.

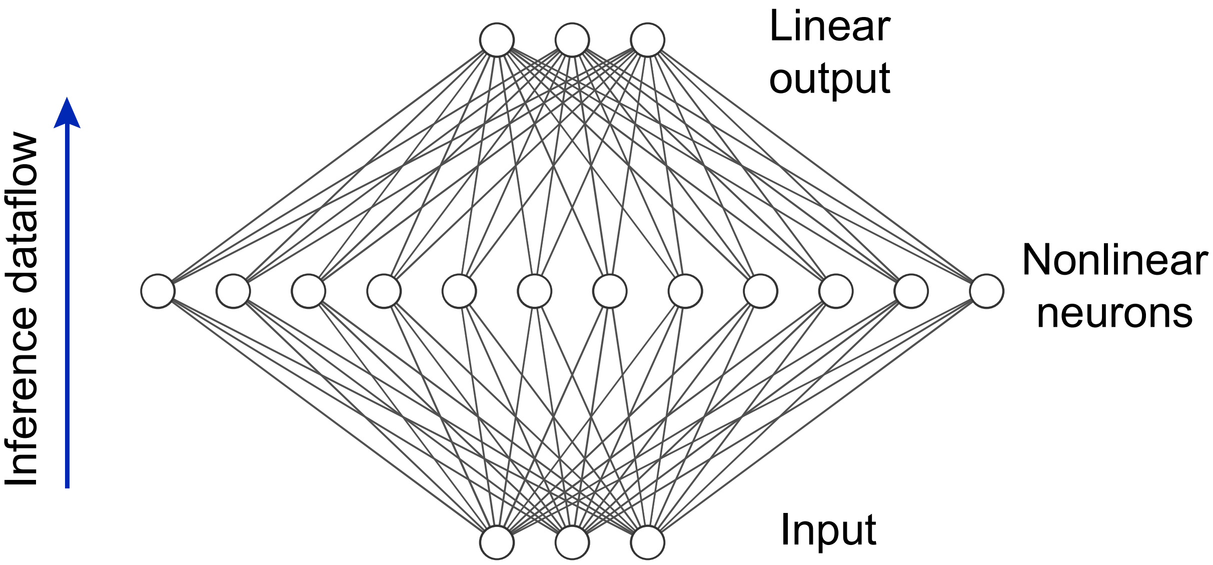

Figure S9: Schematic representation of a bottleneck neural network module comprising two interconnected layers.

Table S2: Comparison of the performance of dendritic models with varying numbers of dendrites per neuron on the LibriSpeech dataset.
The table presents models with two levels of computational complexity. To maintain equivalent computational complexity when increasing the number of dendrites in a neuron, the number of inter-layer channels is proportionally reduced, as indicated in the table.

**# of Dendrites**	**Channel scaling factor**	**Test error**
1 st complexity level(baseline)		
1 (baseline)	1	7.72
4	1/2	7.92
16	1/4	8.28
2nd Complexity level		
1	2	6.69
4	1	6.69
16	1/2	6.89

Table S3: Performance Evaluation of Dendritic Models with varying dendritic counts per neuron evaluated on the LibriSpeech Dataset. The models in this comparison have the same inter-layer communication cost.

# of Dendrites	Channel scaling factor	Test error
1	1/4	15.39
4	1/4	10.49
16	1/4	8.23

References

S1. Cover, T. M., and Thomas, J. A. Elements of Information Theory (Wiley Series in Telecommunications and Signal Processing). Wiley-Interscience (2006).

S2. Murty, U., and Bondy, A. Graph Theory (graduate texts in mathematics 244). Springer (2008).

S3. Kerr, A., Merrill, D., Demouth, J., and Tran, J. Cutlass: Fast linear algebra in cuda c++ (2017). https://developer.nvidia.com/blog/cutlass-linear-algebra-cuda/.

S4. Choquette, J., Gandhi, W., Giroux, O., Stam, N., and Krashinsky, R. (2021). Nvidia a100 tensor core gpu: Performance and innovation. IEEE Micro *41*, 29–35.

S5. Tillet, P. Matrix multiplication; triton documentation (2020). https://triton-lang.org/main/index.html.

S6. Smith, T. M. Theory and practice of classical matrix-matrix multiplication for hierarchical memory architectures. Ph.D. thesis The University of Texas at Austin (2018).

S7. Olivry, A. Automatic derivation of i/o complexity bounds for affine programs. Ph.D. thesis Université Grenoble Alpes (2022).

S8. Krizhevsky, A., Hinton, G. et al. Learning multiple layers of features from tiny images. Tech. Rep. Toronto, ON, Canada (2009).

S9. He, K., Zhang, X., Ren, S., and Sun, J. (2016). Deep residual learning for image recognition. In: Proceedings of the IEEE conference on computer vision and pattern recognition (CVPR). IEEE (770–778).

S10. Hassani, A., Walton, S., Shah, N., Abuduweili, A., Li, J., and Shi, H. (2021). Escaping the big data paradigm with compact transformers. Preprint at arXiv. https://doi.org/10.48550/arXiv.2104.05704.

S11. Panayotov, V., Chen, G., Povey, D., and Khudanpur, S. (2015). Librispeech: an asr corpus based on public domain audio books. In: 2015 IEEE international conference on acoustics, speech and signal processing (ICASSP). IEEE (5206–5210).

S12. Li, J., Lavrukhin, V., Ginsburg, B., Leary, R., Kuchaiev, O., Cohen, J. M., Nguyen, H., and Gadde, R. T. (2019). Jasper: An end-to-end convolutional neural acoustic model. Preprint at arXiv. https://doi.org/10.48550/arXiv.1904.03288.
